# Disturbance regimes drive widespread plant range disequilibrium in Europe alongside climate change

**DOI:** 10.1038/s41559-026-03119-w

**Published:** 2026-07-07

**Authors:** Sean E. H. Pang, Robert Buitenwerf, Oliver Baines, Svetlana Aćić, Markus Bernhardt-Römermann, Idoia Biurrun, Gianmaria Bonari, Hans Henrik Bruun, Chaeho Byun, Eduardo Chacón-Madrigal, Alessandro Chiarucci, Anh Tuan Dang-Le, Pieter De Frenne, Arildo Dias, Jiri Dolezal, Emmanuel Garbolino, Behlül Güler, Nate Hough-Snee, Jens Kattge, Jonathan Lenoir, Adam R. Martin, Sean T. Michaletz, Vanessa Minden, Akira S. Mori, Ülo Niinemets, Wolfgang Schmidt, Mária Šibíková, Grzegorz Swacha, Koenraad Van Meerbeek, Jens-Christian Svenning

**Affiliations:** 1 Center for Ecological Dynamics in a Novel Biosphere (ECONOVO), Department of Biology, Aarhus University, Aarhus, Denmark; 2 Section for Ecoinformatics & Biodiversity, Department of Biology, Aarhus University, Aarhus, Denmark; 3 Department of Biology, University of Oxford, Oxford, UK; 4 Faculty of Agriculture, University of Belgrade, Belgrade, Serbia; 5 Institute of Biodiversity, Ecology and Evolution, Friedrich Schiller University Jena, Jena, Germany; 6 German Centre for Integrative Biodiversity Research Halle-Jena-Leipzig – iDiv, Leipzig, Germany; 7 Senckenberg Institute for Plant Form and Function (SIP) Jena, Jena, Germany; 8 Department of Plant Biology and Ecology, University of the Basque Country UPV/EHU, Bilbao, Spain; 9 Department of Life Sciences, University of Siena, Siena, Italy; 10 National Biodiversity Future Center, Palermo, Italy; 11 Department of Biology, University of Copenhagen, Copenhagen, Denmark; 12 Department of Biological Sciences, Gyeongkuk National University, Andong, Republic of Korea; 13 Herbario Nacional, Museo Nacional de Costa Rica & Centro de Investigación en Biodiversidad y Ecología Tropical, Universidad de Costa Rica, San José, Costa Rica; 14 BIOME Lab, Department of Biological, Geological & Environmental Sciences, Alma Mater Studiorum—University of Bologna, Bologna, Italy; 15 Department of Ecology—Evolutionary Biology, Faculty of Biology—Biotechnology, VNUHCM, University of Science, Ho Chi Minh City, Vietnam; 16 Vietnam National University, Ho Chi Minh City, Vietnam; 17 Forest & Nature Lab, Ghent University, Gontrode, Belgium; 18 FSC International, Bonn, Germany; 19 Institute of Botany, Czech Academy of Sciences, Pruhonice, Czech Republic; 20 Faculty of Science, University of South Bohemia, České Budějovice, Czech Republic; 21 Institut Supérieur d’Ingénierie et de Gestion de l’Environnement, Fontainebleau, France; 22 Biology Education, Dokuz Eylül University, Izmir, Turkey; 23 Meadow Run Environmental, Leavenworth, WA USA; 24 Research Group Functional Biogeography, Max Planck Institute for Biogeochemistry, Jena, Germany; 25 UMR CNRS Ecologie et Dynamique des Systèmes Anthropisés (EDYSAN), Université de Picardie Jules Verne, Amiens, France; 26 Department of Physical and Environmental Sciences, University of Toronto Scarborough, Toronto, Ontario Canada; 27 Department of Botany and Biodiversity Research Centre, The University of British Columbia, Vancouver, British Colombia Canada; 28 Research Group WILD, Department of Biology, Vrije Universiteit Brussel (VUB), Brussels, Belgium; 29 Research Center for Advanced Science and Technology, University of Tokyo, Tokyo, Japan; 30 Chair of Crop Science and Plant Biology, Estonian University of Life Sciences, Tartu, Estonia; 31 Department of Silviculture and Forest Ecology of the Temperate Zones, Georg-August-University of Göttingen, Göttingen, Germany; 32 Institute of Botany, Plant Science and Biodiversity Center, Slovak Academy of Sciences, Bratislava, Slovakia; 33 Botanical Garden, University of Wrocław, Wrocław, Poland; 34 Department of Earth and Environmental Sciences, KU Leuven, Leuven, Belgium; 35 KU Leuven Plant Institute, KU Leuven, Leuven, Belgium

**Keywords:** Plant ecology, Environmental impact, Ecology

## Abstract

Species distributions often fail to match climatically suitable areas, resulting in range disequilibrium. Although recent studies have focused on mismatches arising from lags in biotic responses to climate change (for example, extinction and colonization lags), disturbance processes may also determine whether species occupy otherwise climatically suitable areas, for example, by altering vegetation structure and light regimes. Here we assess climate-change and disturbance-driven disequilibrium for 3,047 vascular plant species across Europe using species distribution models and >1.1 million vegetation plots. Almost all species (99%) show disturbance-driven disequilibrium, revealing mismatches that are strongly structured along disturbance gradients, consistent with alternative vegetation states. We identified disequilibrium associated with recent climate change in 52% of species, with ranges best predicted by climates 10–18 years before sampling and most pronounced among closed-canopy species, probably reflecting under-canopy microclimatic buffering. These findings demonstrate that climate change and disturbance jointly shape widespread disequilibrium in European plant distributions, with disturbance regimes strongly structuring the realization of climatic suitability across contemporary landscapes. Accounting for both disturbance regimes and temporal lags in climate response is therefore essential for improving biodiversity forecasts and guiding conservation strategies.

## Abstract (web summary)

Ecological constraints may prevent species from occupying potentially suitable areas. Here the authors show that disturbance is a widespread predictor of range disequilibrium in the vascular flora of Europe and determines whether plant species can occupy landscapes with suitable climates.

## Main

Understanding the factors driving species distributions across space is key for assessing extinction risks, guiding restoration and rewilding efforts and projecting biodiversity shifts in a rapidly changing world^1^. Species distribution models (SDMs) are frequently used in this context, where such models—and the ecological concepts that underlie their application—typically assume that species distributions are at equilibrium with the prevailing climate conditions. However, species distributions are often at disequilibrium, where ranges of species do not match the prevailing macroclimatic conditions that could sustain long-term persistence^2–6^.

Climate change disequilibrium arises from delays in biotic responses of species to ongoing climatic shifts, including dispersal, establishment and extinction lags—so-called extinction debts and colonization credits^2,7^. Numerous studies have documented these lags, especially along elevation gradients^8–11^. Climate-change disequilibrium is increasingly recognized as widespread, and attention is growing around its implications for conservation planning and long-term persistence of species^2,4,6,7^. However, broad-scale assessments remain limited, particularly across environmental gradients and diverse biogeographic contexts.

Although climate remains central to most distributional studies, disturbance regimes and their effects on local environments are increasingly recognized as critical components shaping realized distributions of species^12–15^. One study^12^ challenged the closed-canopy paradigm, proposing that the prehuman landscapes of Europe were open or semi-open mosaics shaped by large herbivores. Others^16^ similarly highlighted regions exhibiting ‘ecosystem uncertainty’, where both closed and open vegetation are climatically viable, and where disturbance determines the realized state. These perspectives motivate what we term disturbance-driven disequilibrium: a conceptually distinct mechanism contributing to range disequilibrium in which species fail to occupy climatically suitable areas because disturbance regimes maintain alternative stables states of vegetation structure or light regimes that limit or facilitate their occurrence^3,16,17^. Disturbances can both facilitate and restrict species—providing conditions for establishment or persistence but also excluding them when absent or excessively intense^15,18,19^. Unlike the temporal lags underlying climate-change disequilibrium, disturbance-driven disequilibrium reflects how the same macroclimatic conditions can yield contrasting vegetation outcomes depending on natural disturbances such as large-herbivore grazing and fire^13,15,20^, but also anthropogenically altered or replaced disturbances, including defaunation, fire suppression, agriculture and landscape management^21,22^. Although climate change shifts the spatial distribution of climatically suitable conditions, disturbance regimes mediate whether species are able to realize these conditions, thereby potentially shaping how climate-change disequilibrium is expressed in contemporary landscapes. Despite growing research on disturbance–climate interactions at the biome scale, for example, forest–savanna bistability^13,23^, disturbance-driven contributions to range disequilibrium have rarely been quantified at the species level, particularly alongside climate-change-related lags.

Here we investigated climate-change and disturbance-driven disequilibrium across Europe using >1.1 million vegetation plots from the European Vegetation Archive^24^. We quantified disequilibrium by testing whether SDMs accounting for either or both disequilibrium types predicted species distributions better than SDMs that did not. We used ‘standard’ static climate-only SDMs as a common baseline, representing typical modelling practices and providing a reference for identifying model-improvement signals attributable to disequilibrium while controlling for model-inherent errors between models. Our framework captures short-term, plot-scale disequilibrium—reflecting decadal lags and disturbance effects detectable within contemporary vegetation plots—rather than the long-term, postglacial-scale range disequilibrium that arises from historical migration constraints.

To capture climate-change disequilibrium, we developed dynamic SDMs with 5-year moving-window climate averages spanning 0–18 years before the sampling date of each plot, testing whether recent versus past climates better predicted distributions. To capture disturbance-driven disequilibrium, we developed disturbance-informed SDMs that incorporated community-averaged indicator values of light and disturbance severity, the latter at both the whole-community level (‘disturbance severity’ henceforth) and for the herbaceous layer (‘herb-layer disturbance severity’ henceforth)^25,26^. These indicators describe the regimes typical of habitats a species inhabits rather than direct measurements of light or disturbances. Finally, we combined dynamic and disturbance-informed SDMs to test whether accounting for one form of disequilibrium influenced the apparent effect of the other. All models were trained on 1981–2003 plot data, validated on independent 2004–2018 plot data and replicated ten times per species; performance was evaluated using area under the curve (AUC) and logit-transformed for downstream analysis.

We compared model performance using species-specific pairwise *t*-tests on logit-AUC values, applying the Benjamini–Hochberg correction to control false discovery rate. Comparisons were made within each modelling framework, for example dynamic- versus static-climate models to test for climate-change disequilibrium, and disturbance-informed versus climate-only models to test for disturbance-driven disequilibrium. Species were classified as exhibiting significant climate-change or disturbance-driven disequilibrium when the relevant model significantly outperformed the baseline. We hypothesize that range disequilibrium is widespread across the flora of Europe, with disturbance-related constraints contributing strongly to its prevalence due to the continent’s long history altered disturbance regimes and mosaic, disturbance-maintained vegetation structures^14,18,19,21,22,27^. We further hypothesize that accounting for disturbance regimes will reveal substantial disequilibrium not captured by climate-only models and will modify the apparent magnitude and detectability of climate-change disequilibrium.

Next, we tested whether disequilibrium severity was associated with species attributes hypothesized to influence it. Sampling size (number of presence plots) and year (median across training or testing plots) were included to check for data biases, the latter also used to test for expected increases in climate-change disequilibrium under accelerating climate change. For disturbance-driven disequilibrium, we predicted greater severity among species associated with the mid-latitudes and the lowlands of Europe, following the geographical expectation of semi-open, herbivore-maintained mosaics of ref. ^12^ and among species typical of open habitats given the growing evidence for a historically more open and disturbance-maintained Europe^14,18,19,22,27^. Such species are characterized by high light and low disturbance severity (for example, grazing and browsing) indicator values, lower plant height, smaller seeds, greater specific leaf area, smaller leaf area, lower stem density and higher leaf nitrogen (N)^28–30^. For climate-change disequilibrium, we predicted greater severity and longer lags among lowland species, following evidence of greater climate-change disequilibrium at lower elevations in France^8,9^, and among species with slower life histories due to slower demographic turnover^31,32^, characterized by greater plant height, larger seed mass, higher stem specific density and greater leaf area^28,33^.

Finally, to examine the spatial structuring of disequilibrium, we compared the median values of key environmental variables between plots where species were observed as present and where disequilibrium occurred (that is, where the baseline SDM incorrectly predicted presence or absence, but the SDM that accounted for either disequilibrium predicted correctly).

## Results

### Extent and severity of disequilibrium

**Fig. 1:**
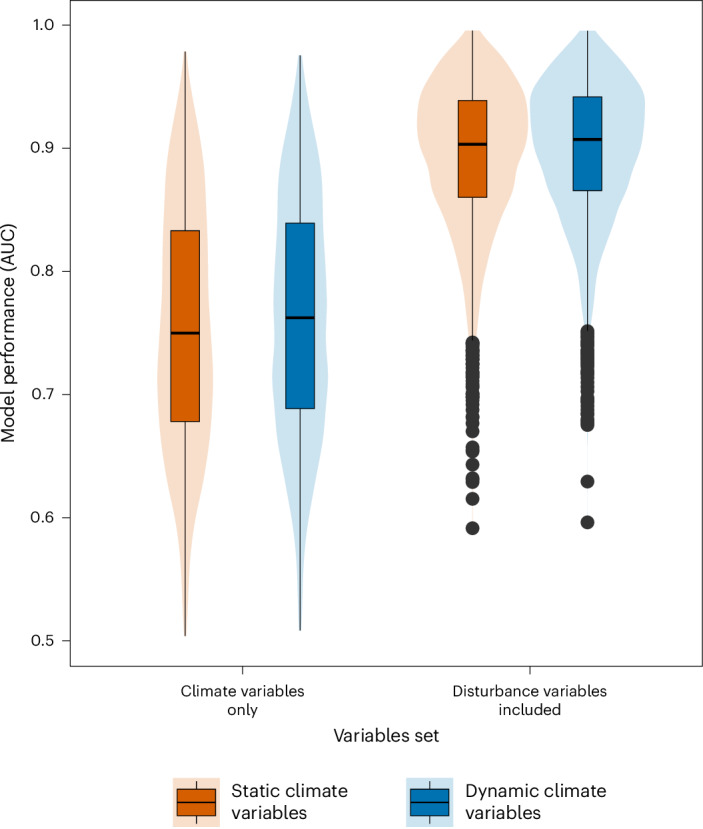
Dynamic climate- and disturbance-related predictors improve the distribution model performance for European plants. Model performance, assessed using AUC, is shown for 3,047 European plant species across models differing in their use of climate variables (static versus dynamic; indicated by colours as shown) and the inclusion of disturbance variables (only climate variables versus including disturbance variables; *x* axis). Box and whisker plots show the median (centre line), interquartile range (IQR) (box boundaries) and the largest and smallest values within 1.5× IQR (whiskers). Static models used long-term climate averages; dynamic models incorporated 5-year moving windows to capture lagged responses. Models including disturbance predictors used community-averaged indicators of light availability, disturbance severity and herb-layer disturbance severity. Between-model differences were tested using LMM with species as a random effect, followed by two-sided Tukey-adjusted pairwise comparisons; all comparisons were significant (*P* ≪ 0.001).

We used differences in model performance as an inferential test for whether species distributions were better explained by climate-only equilibrium assumptions or by models accounting for climate-change or disturbance-driven disequilibrium or both. A total of 3,047 plant species were modelled. Using a linear mixed-effect model (LMM) followed by a Tukey post hoc adjustment, model comparisons revealed that accounting for climate change, disturbance or both significantly improved predictive performance (Fig. 1). Standard SDMs averaged an AUC of 0.755 (±0.100 s.d.). Dynamic climate variables significantly improved AUC for 49.3% of species compared with standard SDMs, although the average gain was modest (+0.008 AUC; *P* ≪ 0.001). Incorporating disturbance variables also substantially improved model performance, significantly increasing AUC for 98.9% of species compared with standard SDMs (+0.140 AUC; *P* ≪ 0.001) (Fig. 1).

Adding dynamic climate variables to models that already incorporated disturbance variables significantly improved AUC for 52.1% of species, but again, the average gain was small (+0.004 AUC; *P* ≪ 0.001). Conversely, incorporating disturbance variables into models that already included dynamic climate variables significantly improved AUC for 98.7% of species (+0.136 AUC; *P* ≪ 0.001). The general trend in model performance was consistent across alternative performance metrics and statistical tests (Supplementary Fig. 1 and Supplementary Table 1).

### Distribution of disequilibrium across species

LMMs related species-level disequilibrium (change in logit-transformed AUC) to species attributes for 2,606 species with complete trait data (Methods).

**Fig. 2:**
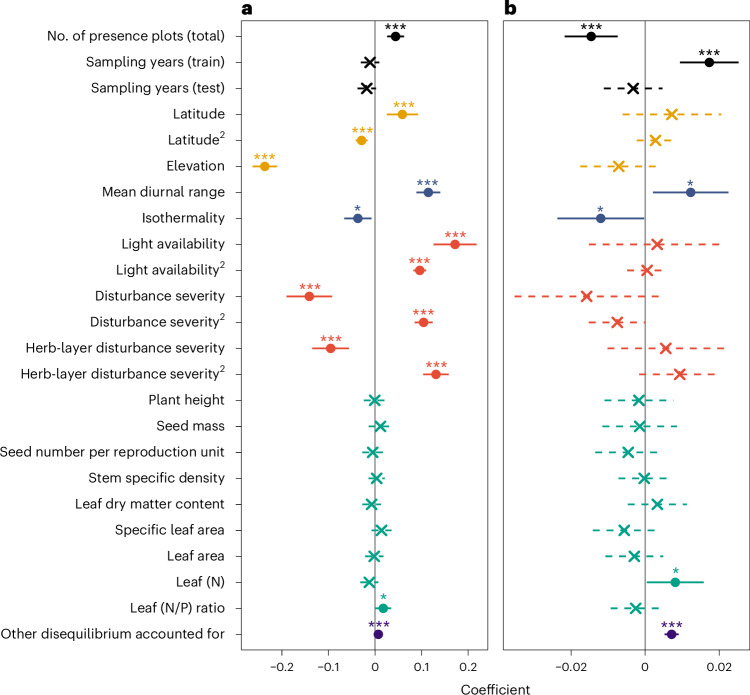
The relationship between disturbance and climate-change disequilibrium severity and various species attributes. **a**,**b**, LMMs were used to assess how various species attributes relate to disturbance-driven (**a**) and climate-change disequilibrium (**b**), measured as differences in logit-transformed AUC, for 2,606 European plant species with complete trait data. Species attributes assessed—from top to bottom—were data characteristics (black), geographic variables (gold), bioclimatic conditions (blue), disturbance-related indicators (orange) and plant functional traits (green), and if the other disequilibrium was accounted for when assessing the focal disequilibrium (purple). For all non-trait attributes, species-level median across presence plots was used. Points indicate model coefficients (solid for significant; cross for non-significant), 95% confidence intervals shown (solid lines for significant effects; dashed for non-significant). Significance levels: *P < 0.05, **P < 0.01 and ***P < 0.001.

Disturbance-driven disequilibrium increased with the number of presence plots, whereas climate-change disequilibrium showed the opposite trend (Fig. 2a,b). Because the number of presence plots was positively correlated with estimated range size (*r* = 0.86), these opposing relationships probably reflect contrasting range-size effects—greater disturbance-driven disequilibrium among widespread species and greater climate-change disequilibrium among range-restricted species. Climate change disequilibrium also increased with more recent training plot data (Fig. 2b), consistent with the acceleration of climate change in more recent years.

**Extended Data Fig. 1:**
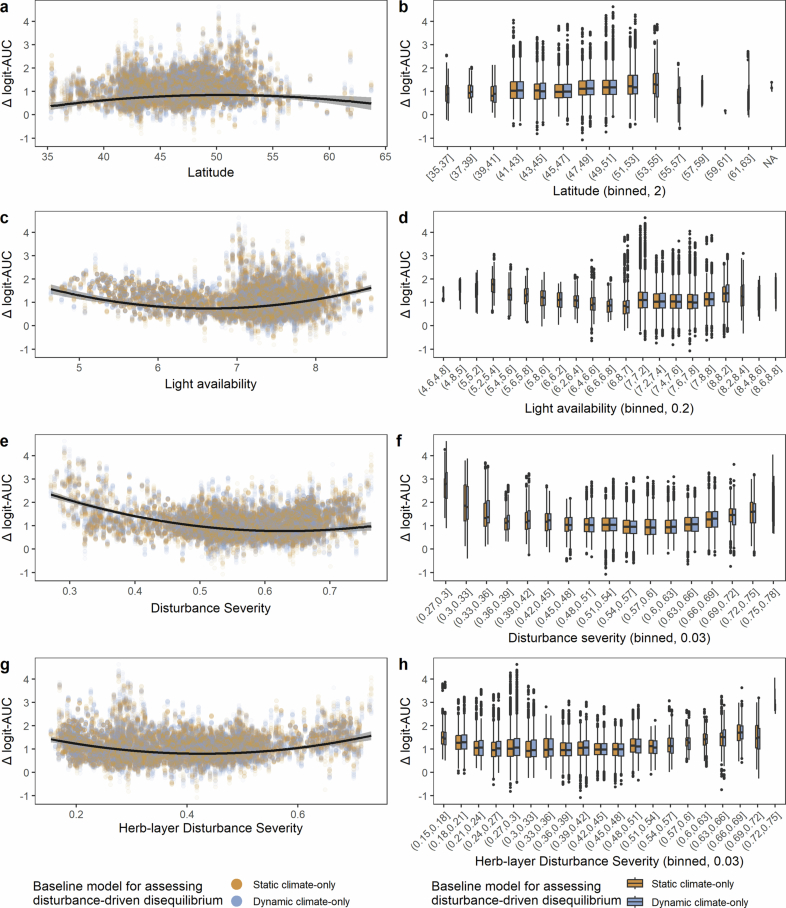
Visualisation of the quadratic relationship between disturbance-driven disequilibrium and four species characteristics for 2,606 species with complete trait data. The distribution of disturbance-driven disequilibrium—quantified as Δ logit-AUC, species-level model comparisons between models that did not account for disturbance-driven disequilibrium and models that did—across four variables: **(a, b)** latitude, **(c, d)** light availability, **(e, f)** disturbance severity, and **(g, h)** herb-layer disturbance severity. Colours represent the baseline model used to assess disturbance-driven disequilibrium—standard static climate-only SDM (light orange) and dynamic climate-only SDM (dark green)—for 10 replicates per species (total 20 data points per species—x-axis values were species constants, that is, a species-level characteristic). **(a, c, e, g)** The modelled quadratic relationships (solid line) as extracted from the linear mixed model used to assess disturbance-driven disequilibrium, with the shaded ribbon representing the 95% confidence interval. Quadratic relationships were significant for all four variables**. (b, d, f, h)** Distribution of disturbance-driven disequilibrium along each species characteristic variable, binned and represented as boxplots. Boxplots show the median (centre line), interquartile range (box boundaries), and the largest and smallest values within 1.5 × the interquartile range (whiskers); points beyond the whiskers are shown as outliers. Boxplot widths reflect sample size (number of species) for each bin. Sample sizes for the boxplots were as follows: **(b)** 240, 470, 560, 3700, 2920, 6120, 5010, 4130, 2040, 560, 210, 30, 10, 50; **(d)** 30, 80, 130, 320, 430, 460, 650, 670, 690, 680, 740, 1110, 2790, 4810, 4110, 3430, 3250, 1260, 290, 110, 20; **(f)** 150, 380, 570, 410, 460, 630, 980, 1860, 4330, 3620, 2240, 2430, 3730, 2760, 880, 570, 60; **(h)** 360, 2090, 1410, 1570, 2700, 1860, 1540, 2120, 2180, 2690, 2180, 1850, 1090, 480, 330, 270, 460, 550, 310, 20.

Geographically, disturbance-driven disequilibrium decreased with elevation and showed a positive, unimodal relationship with latitude (Fig. 2a), peaking at mid-latitudes (~50.5°) (Extended Data Fig. 1). By contrast, climate-change disequilibrium exhibited no significant relationship with either elevation or latitude. Both disequilibrium types were positively associated with mean diurnal range but negatively with isothermality (Fig. 2a,b), patterns partly reflecting continentality; substituting isothermality with longitude (Methods) yielded a positive effect for climate-change disequilibrium (*P* = 0.0336) but not for disturbance-driven disequilibrium (*P* = 0.103) (Supplementary Fig. 3).

Disturbance-driven disequilibrium increased with light availability but declined with both disturbance and herb-layer disturbance severity (Fig. 2a). Positive quadratic relationships indicated more severe disequilibrium at the extremes of each gradient (Fig. 2a), with troughs at intermediate light (~7), disturbance (~0.6) and herb-layer disturbance (~0.4) values (Extended Data Fig. 1).

Among plant functional traits, higher leaf N:P ratio was linked to greater disturbance-driven disequilibrium whereas higher leaf N was associated with greater climate-change disequilibrium (Fig. 2a,b). Most critically, accounting for one disequilibrium generally improved the detection of the other, suggesting partial overlap or interaction between the two processes.

When light availability and disturbance severity were excluded following stricter multicollinearity rules (Methods), additional associations emerged (Supplementary Fig. 4). Greater disturbance-driven disequilibrium was associated with older training plots (*P* = 0.001), shorter plant height (*P* = 0.014), smaller leaf area (*P* = 0.027) and lower leaf N (*P* = 0.005). However, significant relationships with isothermality and the quadratic term for herb-layer disturbance severity were lost. Comparatively, greater climate-change disequilibrium was associated with higher latitudes (*P* = 0.027), while previous associations with isothermality and leaf N were lost.

### Disequilibrium across space

To explore spatial patterns of disequilibrium, we compared the environmental conditions of occupied plots to those where standard SDMs incorrectly predicted presence or absence but were correctly predicted by dynamic or disturbance-informed models (that is, where disequilibrium occurred). We restricted our comparisons to species exhibiting significant disequilibrium and where disequilibrium occurred for at least five plots.

**Fig. 3:**
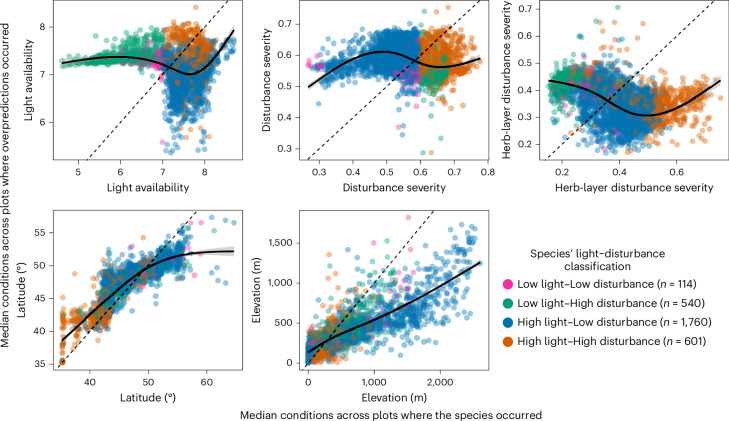
Distribution of species-specific disturbance disequilibrium (overpredictions) along key environmental gradients. Light availability is scored on a 1–9 ordinal scale where 1 represents deep shade and 9 represents full sun or a fully open habitat; disturbance severity and herb-layer disturbance severity represent the proportion of biomass removed during a disturbance event at the community level and at the herb-layer only, respectively. Overpredictions represent plots where standard SDMs predicted presences, but disturbance-informed SDMs correctly predicted absences, indicating potential exclusion from climatically suitable sites due to unsuitable disturbance conditions. For each species, the median value of a given environmental variable across overpredicted plots (*y* axis) was plotted against the median value across occupied plots (*x* axis); each point represents one species. The dashed 1:1 line indicates no difference between overpredicted and occupied conditions. Points above this line reflect higher values among overpredicted plots (that is, the species was predicted in environments with higher values than where it occurs), whereas points below reflect lower values. Generalized additive model (GAM) curves show across-species trends and departures from the 1:1 line, with the shaded ribbon representing the 95% confidence interval. Species were heuristically classified on the basis of thresholds in light availability (7) and disturbance severity (0.6), where GAM lines crossed the 1:1 dashed line. These groups exhibit clear differences in where overpredictions occurred, except for the low-light, low-disturbance group, which was small (*n* = 114). Analyses included species with significant disturbance disequilibrium and at least five overpredicted plots (*n* = 3,015). Because climate-change and disturbance-driven disequilibrium varied depending on whether the other was accounted for, significance estimates and plot predictions were taken from models that included the other effect when it was significant. Variables shown are those with clear deviations from the 1:1 line or of relevance to the hypotheses of this study; see Extended Data Fig. 3 for underpredictions and additional variables.

**Extended Data Fig. 2:**
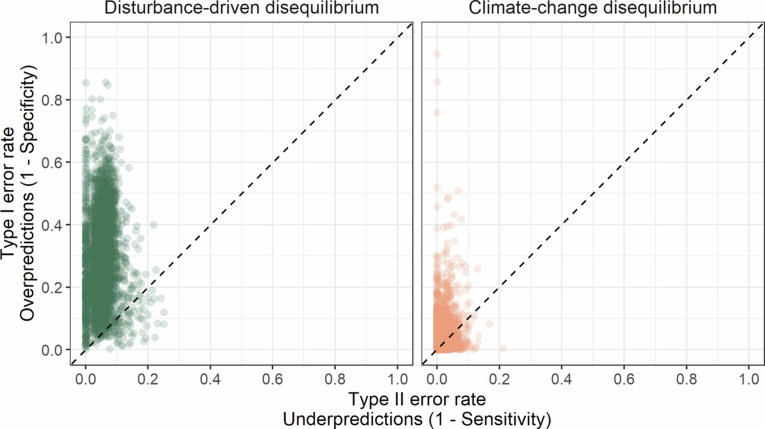
Distribution of over- and underpredictions (Type I and II errors, respectively) across 3,047 European plant species. Distributions are shown separately for disturbance-driven and climate-change disequilibrium. Overpredictions represent plots where standard SDMs predicted presences, but where models incorporating either disturbance-related variables (disturbance-driven disequilibrium) or dynamic climate variables (climate-change disequilibrium) correctly predicted absences, and vice versa for underpredictions. Both disturbance-driven and climate change disequilibrium manifested primarily as Type I errors or overpredictions. Reverse model comparisons—where standard SDMs correctly predicted presence or absence but where disequilibrium-accounted models predicted incorrectly—further confirmed that changes in model behaviour between model that did and did not account for either disequilibrium were predominantly related to the Type I error or overpredictions described above (Supplementary Fig. 5).

**Extended Data Fig. 3:**
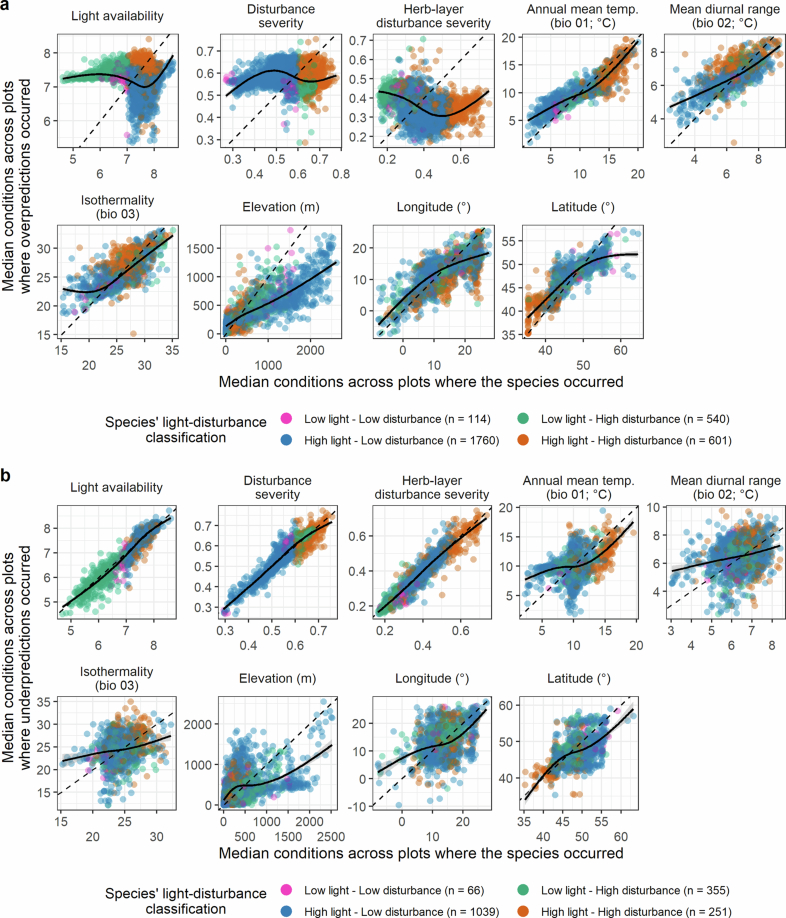
Distribution of species-specific disturbance disequilibrium across 10 environmental gradients for (a) overpredictions and (b) underpredictions. **(a)** Overpredictions are plots where standard SDMs predicted presences but disturbance-informed SDMs correctly predicted absences—indicating likely exclusion due to unsuitable disturbance conditions despite climatic suitability. **(b)** Underpredictions are plots where standard SDMs predicted absences but disturbance-informed SDMs correctly predicted presences—suggesting truncated climatic niches that became detectable when disturbance conditions were included. For each species, the median value of a given environmental variable across over-/underpredicted plots (y-axis) is plotted against the median across occupied plots (x-axis). The dashed 1:1 line indicates no difference in conditions; points above or below show whether over-/underpredictions occur in environments with higher or lower values, respectively. Generalized additive model (GAM) curves show across-species trends (shaded ribbon representing the 95% confidence interval). Species were heuristically classified based on thresholds in light availability (7) and disturbance severity (0.6), determined where GAM lines crossed the 1:1 dashed line for analyses of overpredictions (a). Analyses included species with significant disturbance disequilibrium and at least five overpredicted (*n* = 3,015) or underpredicted (*n* = 1,711) plots. Because climate-change and disturbance-driven disequilibrium varied depending on whether the other was accounted for, significance estimates and plot predictions were taken from models that included the other effect when it was significant.

Disturbance-driven disequilibrium were predominantly overpredictions (30% overprediction versus 5% underprediction on average) (Extended Data Fig. 2 and Supplementary Fig. 5), where standard SDMs predicted presence but disturbance-informed SDMs correctly predicted absence. This represents climatically suitable but unoccupied sites probably constrained by local disturbance conditions at both ends of disturbance-related gradients, as reflected by the bidirectional deviation in overpredictions along those gradients (Fig. 3 and Extended Data Fig. 3). For example, species that typically occurred in low-light environments were frequently overpredicted in high-light plots, whereas those associated with high light were often overpredicted in shaded plots. This gradient-dependent deviation also occurred along disturbance and herb-layer disturbance severity gradients; although high-light, high-disturbance species exhibited this deviation primarily along the herb-layer disturbance gradient. These deviations reversed near intermediate values, roughly corresponding to the troughs in disturbance-driven disequilibrium for those disturbance-related gradients (Fig. 2).

Overpredicted plots were slightly skewed towards central Europe (40°–52°) (Fig. 3). Along elevation, high-elevation species tended to be overpredicted at progressively lower sites, indicating that downslope habitats were probably climatically suitable but unoccupied due to disturbance-related processes.

**Extended Data Fig. 4:**
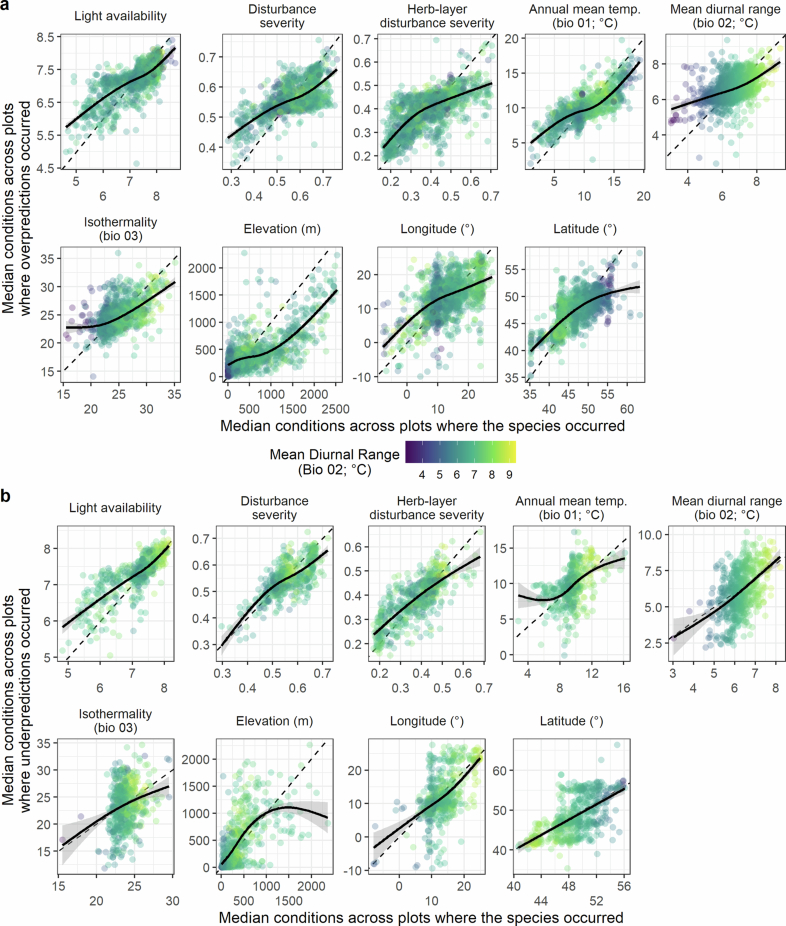
Distribution of species-specific climate-change disequilibrium across 10 environmental gradients for (a) overpredictions and (b) underpredictions. **(a)** Overpredictions are plots where standard SDMs predicted presences but dynamic SDMs correctly predicted absences—indicating where static variables lead to overestimations of suitability. **(b)** Underpredictions are plots where standard SDMs predicted absences but dynamic SDMs correctly predicted presences—indicating where excluding climate dynamics led to a truncation of the estimated climate niche. For each species, the median value of a given environmental variable across over-/underpredicted plots (y-axis) is plotted against the median across occupied plots (x-axis). The dashed 1:1 line indicates no difference in conditions; points above or below show whether over-/underpredictions occur in environments with higher or lower values, respectively. Generalized additive model (GAM) curves show across-species trends (shaded ribbon representing the 95% confidence interval). Species were coloured based on the most important predictor of climate-change disequilibrium when analysing severity-attribute associations: temperature annual range. Analyses included species with significant climate-change disequilibrium and at least five overpredicted (*n* = 1,540) or underpredicted (*n* = 685) plots. Because climate-change and disturbance-driven disequilibrium varied depending on whether the other was accounted for, significance estimates and plot predictions were taken from models that included the other effect when it was significant.

Climate change disequilibrium comprised slightly more overpredictions (4% overprediction versus 1.7% underprediction on average) (Extended Data Fig. 2 and Supplementary Fig. 5), suggesting that static climate variables tended to incorrectly predict presence in plots where dynamic climate variables correctly predicted absence. Only elevation revealed a clear spatial signal: overpredicted and true presence plots diverged beyond 500 m with overpredictions consistently occurring at lower sites (Extended Data Fig. 4).

### Lags in plant range responses

**Fig. 4:**
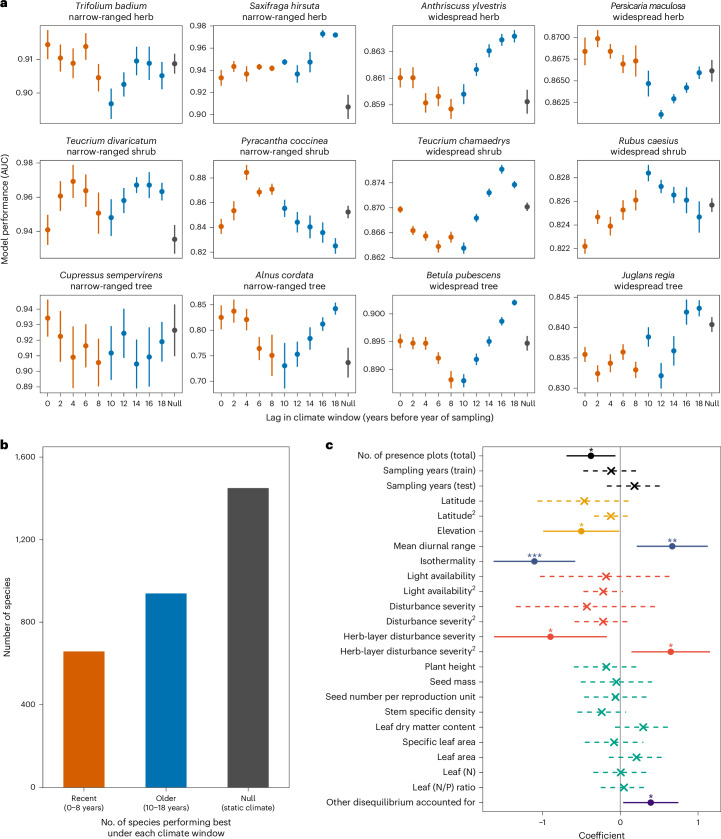
Temporal lags in distributional responses of species to climate change. **a**, Climate-window performance across 12 species showing significant climate-change disequilibrium. Species were randomly selected to represent combinations of range size (narrow-ranged and widespread) and growth form (herb, shrub and tree). Models tested 5-year moving-window climate averages spanning 0 to 18 years before the sampling year (in 2-year increments); points show mean performance (AUC) across ten replicates, with error bars indicating standard deviation. Colours indicate the binning of the window as recent (orange), older (blue) or null (grey). ‘Null’ indicates the performance of static-climate, standard SDMs. **b**, Distribution of the best-performing climate windows across species, summarized into recent (0–8 years; orange) and older (10–18 years; blue) windows. Species without significant climate-change disequilibrium were assigned to the ‘null’ bin (static climate; grey). In **b** and **c**, because climate-change lags varied depending on whether disturbance-driven disequilibrium was already accounted for, we used estimates from comparing models that included disturbance effects when disturbance-driven disequilibrium was significant (98.7% of species); otherwise, we used estimates from comparing climate-only SDMs. **c**, Species attributes associated with greater temporal lags in climate responses, based on the best-performing window for 1,735 European plant species with complete trait data and significant climate-change disequilibrium (829 species were significant for only one comparison—either between static-climate-only and dynamic-climate-only SDMs or between disturbance-informed static and dynamic SDMs). An LMM related lag length to species-level attributes, including data characteristics (black), geographic variables (gold), bioclimatic conditions (blue), disturbance indicators (red) and plant functional traits (green), and indicated if the other disequilibrium was accounted for when assessing the focal disequilibrium (purple). For all non-trait attributes, species-level median across presence plots was used. Points indicate model coefficients (solid for significant and cross for non-significant), 95% confidence intervals shown (solid lines for significant effects, dashed for non-significant). Significance levels: *P < 0.05, **P < 0.01 and ***P < 0.001.

**Extended Data Fig. 5:**
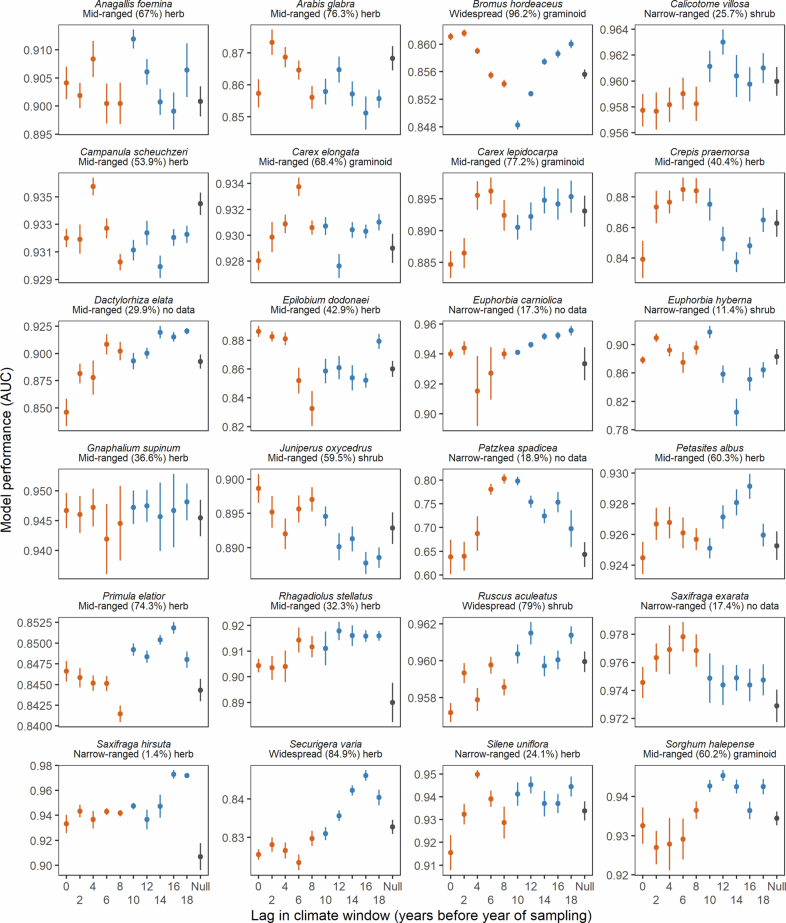
Climate window performance across 24 randomly selected species with significant climate-change disequilibrium. Each panel title contains the species’ name, range size label, range size’s percentile, and its growth form (labelled “no data” when unavailable). Models tested 5-year moving-window climate averages spanning 0 to 18 years before the sampling year (in 2-year increments); points show mean performance (AUC) across 10 replicates, with error bars indicating standard deviation. Colors indicate the window’s binning as recent (orange), older (blue), or null (grey). “Null” indicates the performance of standard static-climate-only SDMs. Because climate-change disequilibrium varied depending on whether disturbance-driven disequilibrium was accounted for, significance estimates and window performances were taken from models that incorporated disturbance predictors when disturbance-driven disequilibrium was significant.

**Extended Data Fig. 6:**
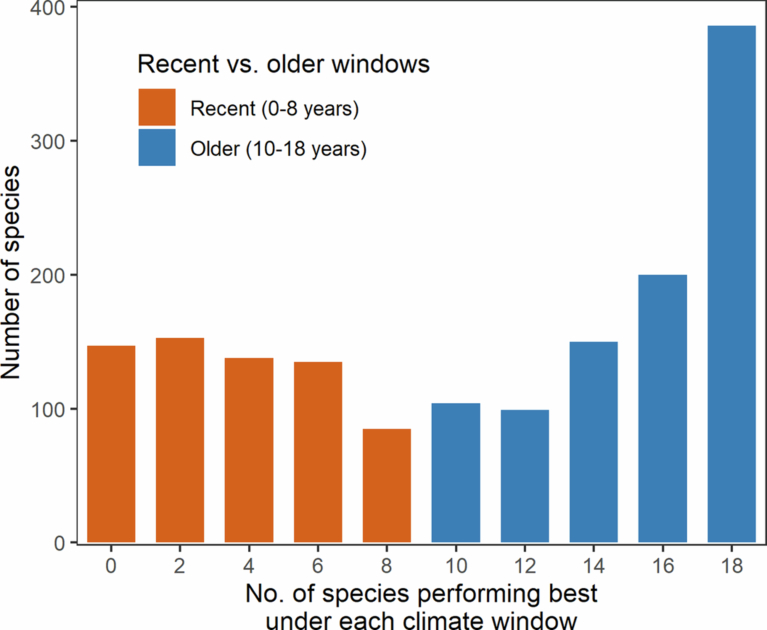
Distribution of the best-performing climate windows across species. Windows are coloured as recent (0 – 8 years; orange) and older (10 – 18 years; blue). Plot shows a total of 1,597 species. Species without significant climate-change disequilibrium were excluded. Because climate-change disequilibrium varied depending on whether disturbance-driven disequilibrium was accounted for, significance estimates and selected best windows were taken from models that incorporated disturbance predictors when disturbance-driven disequilibrium was significant.

Species responses to climate windows exhibited distinct but informative patterns. Across 12 representative species spanning growth forms and range sizes (Fig. 4a), responses included unimodal increases in performance up to an older (for example, *Rubus caesius* and *Juglans regia*) or more recent optimal window (for example, *Pyracantha coccinea*), sharp performance peaks (for example, *Saxifraga hirsuta*) and in some cases exhibiting a less distinct trend across windows (for example, *Cupressus sempervirens*). The most common pattern, however, was bimodality, with both older and more recent performance peaks. In some cases, both peaks outperformed the null model (static climate SDM) (for example, *Anthriscus sylvestris*, *Teucrium divaricatum* and *Alnus cordata*), whereas in others the second peak did not (for example, *Trifolium badium*, *Persicaria maculosa*, *Teucrium chamaedrys* and *Betula pubescens*). Importantly, these patterns were not limited to selected growth forms or range sizes, as shown through an additional randomly selected set of 24 species (Extended Data Fig. 5). Despite variation among species, a general tendency emerged: best-performing windows were predominantly older (10–18 years), occurring ~30% more often than recent windows (0–8 years) (Fig. 4b), with the 18-year window most frequently being optimal (Extended Data Fig. 6).

We next explored whether variation in lags of species was associated with attributes of species (Fig. 4c). Longer lags were associated with fewer presence plots and lower-elevation species. Lags were also associated with higher mean diurnal range and lower isothermality; substituting isothermality with longitude (Methods) yielded a positive effect (*P* = 0.001), suggesting bioclimatic patterns to partly reflect continentality. Lags exhibited a nonlinear relationship with herb-layer disturbance severity: a negative linear and positive quadratic term indicated that lags were longest for species associated with low herb-layer disturbance environments, shortest at intermediate values (~0.47) and increased again at higher values. No relationship emerged for functional traits. Interestingly, accounting for disturbance-driven disequilibrium resulted in longer lags, suggesting that disturbance effects otherwise mask lag signals.

When herb-layer disturbance severity was excluded because of multicollinearity, a negative association with light availability emerged (*P* = 2.054 × 10^−3^), indicating greater lags among species typical of shaded, closed-canopy habitats. When light availability and disturbance severity were excluded instead, the negative relationship with elevation was lost and a negative association with stem specific density emerged (*P* = 0.061; Supplementary Fig. 4).

## Discussion

Our study provides a, to our knowledge, the first pan-European assessment of disturbance-driven and climate-change disequilibria in plant distributions, revealing marked differences in their prevalence, spatial organization and underlying mechanisms. Disturbance-driven disequilibrium was widespread and strongly structured along disturbance-related gradients, reflecting persistent constraints on the realization of climatic suitability across contemporary landscapes. Disequilibrium associated with recent climate change was also evident in about half of all studied species but was less strongly expressed at present and less spatially structured. Together, these contrasting patterns highlight disturbance as a key, yet often underappreciated, contributor to range disequilibrium, while highlighting the distinct and emerging imprint of climate change on plant distributions. These findings stress the need to incorporate both processes into ecological models and conservation strategies^13,15,16,20,34^.

Disturbance-driven disequilibrium was stronger among widespread species, reflecting greater exposure to variation in disturbance regimes and anthropogenic modification. The signal may also partly reflect temporal variation, because species with older training data better capture historical disturbance–climate relationships^5,35^, potentially linked to the strong twentieth-century shift towards closed-canopy landscapes across Europe^36,37^. However, severity did not solely peak among species typical of open habitats, that is, those associated with higher light and lower disturbance severity values. Instead, severity was greatest at both ends of these gradients, indicating that both open- and closed-habitat species were most affected and that much of Europe supports bistable vegetation dynamics^16,23^. The intermediate troughs in severity thus probably marked transition zones where disturbance regimes tip between open- versus closed-canopy vegetation. Spatial mismatches mirrored this pattern: species associated with more open or closed habitats showed the most pronounced overpredictions, whereas those from intermediate environments exhibited weaker spatial patterns. Species at the extremes are more sensitive to such shifts because they depend more on disturbance regimes than on climate to maintain their preferred stable state^16,20,25^, whereas those near intermediate values are less affected, probably persisting across a broader range of disturbance conditions.

Trait associations reinforced this pattern. Greater disequilibrium was contrastingly observed among short-stature species with smaller leaf area, typically fast-growing and characteristic of open-habitat species and species with lower leaf nitrogen and higher leaf N:P ratios, traits associated with slow-growing, closed-canopy species^29,33,38^. Some of these relationships only emerged when light availability and disturbance severity were excluded, suggesting these traits to partly capture structural- or disturbance-related gradients represented by those predictors. Together, these findings most likely reflect a signature of Bond’s ecosystem uncertainty^16^, where both closed and open vegetation are climatically viable and the disturbance regime determines the realized state. Disequilibrium severity also peaked among mid-latitude and lower-elevation species, coinciding with the expectation of where forest–grassland mosaics occurred of ref. ^12^, suggesting that herbivore-driven disturbance, or its absence, may underlie much of the observed disequilibrium.

These findings reaffirm the importance of disturbance-mediated habitats that both supports higher biodiversity and reflect dynamic systems dependent on natural and anthropogenic disturbance regimes^19,21,22^. The current disturbance regimes of Europe are overwhelmingly anthropogenic, with natural disturbance processes suppressed in most areas as a result of megaherbivore extinctions and to optimize agriculture and silviculture, but boosted because of overgrazing in most livestock-grazed ecosystems^21,22^. Disturbance-driven disequilibrium therefore highlights where historical ecological baselines and restoration targets may need revision to account for altered disturbance dynamics^19^.

Climate-change disequilibrium remains a growing concern under accelerating climate change^6,8–11^. Despite the coarse resolution of climate data used, our results show disequilibrium due to recent climate change as a significant feature of European flora, underscoring the early and ongoing imprint of climate change on plant distributions. The correlates of climate-change disequilibrium collectively point to limited climatic tracking capacity, constrained by both environmental context and species traits. Disequilibrium severity was tied to climate change severity: greater disequilibrium severity among species occupying seasonal (or continental) and potentially higher-latitude regions that exhibit higher climate velocities^39^, and also among species with more recent plot data, consistent with accelerating climate change. Narrow-range species showed both greater disequilibrium severity and longer lags, probably reflecting higher proportional exposure to edge dynamics and more limited dispersal capacity further slowing migration rates at the leading edge^40,41^. Similarly, our finding of greater lags among low-elevation species aligns with previous elevation studies, and potentially stemming from greater habitat fragmentation and geographic distances when tracking equivalent climatic shifts at lower elevations^8,42^. Longer lags were also observed among species typical of closed-canopy environments, where there is lower light availability and herb-layer disturbance severity, representing potential evidence of microclimatic buffering delaying species responses to macroclimatic changes^43,44^. Species associated with high herb-layer disturbance severity also exhibited longer lags, potentially reflecting disturbance-adapted traits such as being short-lived or reliant on episodic canopy openings—strategies that support persistence under frequent disturbance but may hinder longer-term climate tracking where disturbance regimes are spatially fragmented or suppressed.

Our results show that climate-change disequilibrium is less strongly expressed at present but increasingly detectable, underscoring its growing biological relevance. This emerging signal aligns with broader expectations that biotic responses—and their lags—to ongoing climate change will continue to intensify^2,4,7,39^. Accounting for disturbance regimes proved important, since unmeasured disturbance effects can mask or distort climate-change disequilibrium, and controlling for climate lags similarly improved the detection of disturbance-driven disequilibrium. Notably, both processes contributed to upward skews in present-day elevation ranges, reflecting disturbance-related exclusion from climatically suitable low-elevation sites^45,46^ and upward range shifts that may go undetected when distribution models use static climate variables^6,41^. Supporting this, ref. ^47^ showed that alpine species can expand downslope along disturbed mountain roads, where relaxed competition from lowland vegetation permits persistence beyond typical lower elevational limits. Combined with heightened severities of both disequilibria among lower-elevation species, these results highlight strong potential for interactions and confounding effects between disturbance-driven and climate-change dynamics, emphasizing the need to examine them jointly.

A notable result was the frequent occurrence of bimodal climate-window responses, where species distributions were simultaneously associated with both relatively recent and substantially older climate windows. This pattern suggests that climate-change disequilibrium may often reflect several temporally distinct processes rather than a single characteristic lag, potentially arising from differing responses among demographic processes, climatic drivers or spatial dynamics between leading and trailing range edges^2,5–7,41,48^. Although our framework was not designed to partition these mechanisms explicitly, the prevalence of bimodality suggests that temporally heterogeneous climate tracking may be an additional feature shaping European plant distributions.

Climate change may interact with disturbance regimes^49^, while land management or active rewilding may buffer or amplify climate change impacts^34^. Yet, most modelling approaches continue to treat climate change as the primary—often sole—axis of disequilibrium, whereas conservation strategies more commonly target disturbance-linked disequilibrium. Our results demonstrate that disturbance dynamics impose strong and pervasive constraints on the current distributions of species—particularly in historically modified regions like Europe—echoing findings that human land use is comparable to climate in shaping plant occurrence and abundance patterns^50^. Although broad anthropogenic layers (for example, land use and land cover) are increasingly incorporated into SDMs, disturbance-related predictors within seminatural habitats remain underused, despite their demonstrable effects on model performance and recent call to integrate local habitat conditions^44^. Recognizing disturbance disequilibrium as a distinct and measurable phenomenon can improve the realism of SDMs, reduce overpredictions, refine vulnerability assessments and support restoration targets that better reflect ecological history and dynamic^5,17,35^.

Although our findings offer robust evidence of widespread distributional mismatches across the flora of Europe, several limitations highlight directions for future research. Our disturbance-related predictors captured resulting vegetation structure and composition rather than direct measurements of disturbance processes^25,26^, limiting insights into specific drivers and temporal dynamics^5,7^. Likewise, our climate-window approach identifies lagged responses to macroclimatic trends but necessarily constrains detection to a single dominant window, which assumes that short-term climate-change disequilibrium can be adequately summarized in this way—an assumption unlikely to entirely hold and one that implies that our estimates of climate-change disequilibrium were conservative. Temporal biases, species lacking more recent plot data, further contribute to this under-detection. Residual spatial biases and potential niche truncation could also influence reported severities, although the continental scope and data thinning (Methods) probably minimize their impact. These limitations emphasize the need for approaches that integrate finer-scale disturbance data, or link SDMs with demographic or mechanistic frameworks to better disentangle the processes underpinning disequilibrium^4,6,48,51^. Our moving-window approach nonetheless detected widespread lagged responses even within the 18-year period analysed. The prevalence of static or extreme-window optima suggests that response lags for many species may extend beyond the available climate record, particularly for long-lived or slow-responding species^31,32,43^, indicating that the lags identified here probably represent only a short-term component of broader climatic disequilibria documented at longer timescales (for example, postglacial^52^). Most critically, the frequent occurrence of bimodal window responses—potentially reflecting varying lags among different demographic rates or climate variables—further highlights the need for more flexible approaches that can accommodate several or variable-specific lags while remaining tractable within data-efficient modelling frameworks^2,4,5,48^. Finally, the disturbance indicators used here are well-calibrated for European vegetation but may be less transferable to regions or taxa where disturbance operates differently, such as monsoonal or drought-dominated systems. Nonetheless, emerging global remote sensing data on vegetation structure, fire and land-use intensity offer clear opportunities to extend this framework beyond Europe. Applying this framework to other continents will test whether the prominence of disturbance-driven disequilibrium reflects a general ecological principle or Europe-specific legacies^14,15,18,21,22^. We expect similar dynamics to be particularly relevant in other temperate, dry tropical and savanna landscapes with medium-productivity climate conditions, and where herbivory and fire maintain open–closed vegetation mosaics^3,12,16,23^.

Our pan-European analysis reveals that distributional disequilibrium is widespread across the flora of Europe, reflecting the combined influence of disturbance regimes and ongoing climate change. Disturbance-related constraints currently leave a strong imprint on realized plant distributions, and the growing signal of short-term climate-change disequilibrium highlights the urgency of anticipating future shifts and response lags that will intensify as climate change accelerates. Accounting for both disturbance regimes and temporal lags in climate response will be essential for building more realistic models and for guiding conservation strategies based on realized ecological constraints in a rapidly changing and increasingly human-shaped world.

## Methods

### Plot and environmental data

We retrieved 1,903,668 vegetation plots and 40,468,042 observation records from the European Vegetation Archive (EVA; Project 189; accessed 24 August 2023; ref. ^24^), including plots from the ReSurveyEurope initiative^53^ and associated LOTVS, GLORIA and forestREplot databases^54–56^; this included single-sampled and resampled plots, which were not treated differently. We selected for plots within Europe, but excluding Ukraine, Russia, Belarus and Turkey. Plots were further filtered to exclude those with location uncertainty exceeding 5 km, noted as experimentally manipulated, lacking date of recording or sampled outside our study period (1981–2018) (Supplementary Section 1). The lower bound of 1981 ensured that all plots had at least 20 years of preceding climate data (from 1961) for lag analyses. After filtering, 1,138,587 plots remained (Supplementary Fig. 6); 91,260 did not contain coordinate uncertainty information but were retained.

A total of 22,083,578 observation records of vascular plants were obtained from these plots. We removed observations with taxonomic identification above the species level (including section, series and subgenus), hybrids, uncertain identities and cultivated individuals. To standardize species identities for modelling, we consolidated infraspecific taxa and taxonomic qualifiers under their corresponding species names, grouping subspecies, varieties, forma and other infraspecific entities that are often not distinguished or misidentified^57^. Although this approach simplifies the data and facilitates modelling, we acknowledge that it may not fully capture the ecological variability within some species complexes. A total of 21,639,385 observations remained. All subsequent analyses were performed at the scale of individual vegetation plots.

All climate data were sourced from the UERRA regional reanalysis for Europe dataset (retrieved 18 October 2023; Copernicus Climate Change Service 2019 (ref. ^58^)). Daily near-surface (2 m above ground) temperature and precipitation records from 1961 to 2018 were obtained from the MESCAN-SURFEX system (0.5° by 0.5°), representing macroclimatic conditions. Although older palaeoclimatic datasets exist, major anthropogenic climate shifts began around the 1960s, making data before this period less relevant for capturing biotic response to recent climate change (last century). Accordingly, we limited our analysis to the post-1960 period to enhance detectability of biologically meaningful lags in response to modern climate trends. Daily climate data were aggregated into 21 annual climate variables for each year: 15 bioclimatic variables (excluding precipitation variables based on temperature-defined periods and vice versa), three growing degree day variables (0 °C, 5 °C and 10 °C thresholds), tree growth season length, snow cover days and frost change frequency (Supplementary Section 1 and Supplementary Table 2). This resulted in annual layers for all 21 variables across 1961–2018, providing temporally explicit climate data for dynamic modelling. After excluding highly correlated climate variables (*r* ≥ 0.7), seven predictors were retained: annual mean temperature, mean diurnal range, isothermality, temperature seasonality, annual precipitation, precipitation seasonality and frost change frequency. These variables were used in both static and dynamic SDMs.

Light indicator values were sourced from a harmonized dataset of Ellenberg-type values at the European scale^26^, using species aggregate values when available (that is, values averaged across closely related or difficult-to-distinguish taxa within a species complex). These values reflect the typical light conditions, or light availability, under which species are found, ranging from deep shade to full sunlight (1–9). Disturbance indicator values were sourced from a European database^25^, including disturbance severity, herb-layer disturbance severity, disturbance frequency and herb-layer disturbance frequency. For our final species list, herb-layer disturbance severity was highly correlated with both disturbance frequency (*r* = 0.70) and herb-layer disturbance frequency (*r* = 0.76) and thus, frequency indicators were excluded. Disturbance severity represents the expected proportion of biomass removed or destroyed at the whole-community level during a disturbance event; herb-layer disturbance severity reflects the same process but restricted to the herbaceous field layer, and is therefore always equal to or lower than disturbance severity^25^. These disturbance indicators do not record disturbance events or biomass loss directly; instead, they reflect the disturbance conditions characteristic of the habitats in which a species typically occurs, and therefore describe ecological associations of species with disturbance rather than disturbance per se. In total, three disturbance-related predictors were used: light availability, disturbance severity and herb-layer disturbance severity.

Following ref. ^26^, a minimum of five species per plot was required for reliable community-averaged indicator value calculation. To avoid circularity—where presence of a species would influence its own disturbance predictor—we excluded the focal species when calculating plot-level indicators for that species (that is, plot-level indicators were calculated separately for each species)^17^. Thus, plots were required to contain at least six species, ensuring that five remained even after focal species exclusion. This approach kept the set of eligible plots consistent across all species. Applying this criterion excluded 136,580 plots.

To calculate plot-level community-averaged indicator values, we used the unweighted average across species within a plot. When testing weights, we found a high correlation (*r* = 0.95) between unweighted and weighted values (based on percentage cover of the plot by species), supporting the use of the simpler unweighted approach. We focused on light availability and disturbance regimes because of their strong, known links to disturbance processes^25^.

To assess whether the disturbance and light indicators reflected independently observable forest structural conditions, we compared a subset of plots against remote sensing-derived European tree extent and tree height products^59^. Herb-layer disturbance severity and light availability showed stronger relationships with local-scale remotely sensed forest structure than disturbance severity, with relationships generally weakening at larger spatial buffers (Supplementary Table 3).

### Species distribution modelling

We developed SDMs to investigate climate-change and disturbance disequilibrium across European plants (Supplementary Section 2). Given the focus on detecting disequilibrium and lagged responses, we designed the modelling framework to ensure temporal independence between model training and evaluation. Plots were separated into two temporal bins: a training bin (1981–2003) for model training and a testing bin (2004–2018) for climate-window selection and model validation. This separation served three purposes. First, it ensured temporal independence between model calibration and evaluation and allowed genuine forward projection across a period of known climate change. Second, it reduced temporal heterogeneity within each bin, limiting noise from short-term variability or uneven sampling that could obscure longer-term trends. Third, it maintained sufficient sample size and spatial coverage in both periods while providing a conservative test for temporal disequilibrium—models trained on earlier data and evaluated on later data perform worse when species–environment relationships have shifted. This two-bin structure therefore provides a robust yet data-efficient framework for quantifying disequilibrium under recent environmental change.

Our modelling framework explicitly tests the assumption of climatic equilibrium inherent to conventional SDMs. Static climate models represent the equilibrium expectation, assuming that occurrences of species reflect current climatic suitability averaged over multidecadal periods. By contrast, dynamic models allow quasi-equilibrium states to shift between temporal bins by incorporating time-varying climate predictors. The difference in predictive performance between these two model types quantifies the degree to which observed occurrences of species deviate from static climatic expectations under ongoing climate change. In this context, occurrence data are interpreted not as perfect reflections of present-day climate suitability but as outcomes of past or lagged climatic conditions, enabling our framework to detect both the strength and temporal depth of climate-change disequilibrium.

Species presence data were spatially thinned with a 10-km buffer to reduce spatial autocorrelation and the over-representation of densely sampled regions; that is, presences occurring within 10 km of another were removed, prioritizing more recent records (Supplementary Section 2). Thinning was applied separately for each temporal bin to ensure comparable spatial distributions between training and testing datasets. This procedure substantially reduces clustering bias in the underlying vegetation plot data, improving the spatial independence of observations and preventing inflated model performance due to pseudoreplication. By thinning within bins rather than across them, we also minimize confounding spatial structure with temporal turnover, ensuring that modelled changes in species–environment relationships reflect true temporal dynamics rather than uneven spatial sampling. Species with ≥35 training and ≥10 testing presences were retained, yielding 3,047 species for modelling. Absence data comprised plots without the target species and within 300 km of its presence plots to better represent true absences due to climatic- or disturbance-related factors rather than dispersal limitations. This accessible-area constraint helps minimize the inclusion of absences that were probably dispersal-limited—particularly those arising from environmental changes that predate our study period—while retaining broad climatic and biogeographic representativeness across Europe. Absences were binned and thinned identically to presence plots to maintain consistent spatial independence and sampling density across both temporal periods.

Four SDM types were fitted to the presence–absence plot data of each species, differentiated by their predictor set. (1) Standard SDMs used static climate variables averaged over the temporal bins (1981–2003 for training and 2004–2018 for projecting and validation). Standard SDMs served as baselines and were deliberately kept simple to avoid embedding disturbance or temporal dynamics within the reference model, ensuring that any improvement in performance directly reflected the added ecological processes rather than differences in model complexity. (2) Dynamic SDMs used 5-year moving-window climate averages, calculated over ten lag intervals spanning 0–18 years (in 2-year increments) before the date of recording or year of sampling for a plot; 5-year climate windows spanned ±2 years from the selected lag year. For zero-lag windows, climate data after the sampling year were ignored, resulting in a 3-year average. Because sampling years varied across plots, the climate years included in a given lag window also varied between plots. As a result, lag effects were captured at the species or distributional scale (reflecting overall shifts in climatic associations of species) rather than at the level of local population- or plot-specific responses. Only one lag period was considered at a time because testing different lag periods among climate variables would result in an unmanageable number of models (from 10 to 10^7^ dynamic models per species). The best-performing window was selected by evaluating resulting model projections on a subset of presence–absence data from the testing bin (see below); models with different windows were assessed and compared using AUC. (3) Disturbance-informed SDMs used static climate variables (as done for standard SDMs) and disturbance-related predictors (as calculated above, unweighted plot-level community-averaged indicator values of light availability, disturbance severity and herb-layer disturbance severity). Target species were excluded from indicator calculations for presence plots to prevent circularity, so calculations were performed separately for each species^17^. (4) Dynamic, disturbance-informed SDMs combined both lagged climatic predictors and disturbance indicators.

Models were trained using a randomly selected 90% subset of presence–absence data from the training bin (1981–2003). We used GAMs, fitted using the mgcv R package (v.1.9.0) with a binomial family, logit link function and smoothing parameter estimation via restricted maximum likelihood; other settings were left as defaults. The basis dimension (*k*) for smooth terms was limited to four to constrain model flexibility, improving biological plausibility by preventing overly complex response shapes and reducing overfitting to noise by limiting the number of allowable inflection points independent of smoothing parameter optimization^60^. To keep predictor numbers constant across modelling protocols, disturbance-uninformed SDMs used the same disturbance-related predictors but with values that were randomly permutated (for both training and testing data bins).

Testing data (2004–2018) were split into two equal subsets: one used to select the best-performing climate window for SDMs using dynamic climate variables; and the other reserved for independent model validation. This separation avoided information leakage between calibration (window selection) and validation steps, ensuring that model evaluation remained unbiased. Using the same data for both window selection and model validation would have artificially inflated model performance and exaggerated the apparent significance of climate-change disequilibrium by overfitting—or, more specifically, over-calibrating—the models to the specific windowed climate conditions. Model performance was assessed using AUC, a popular discriminatory metric that evaluates probabilistic accuracy (or continuous habitat suitability predictions). Model performance was assessed using plots reserved for independent model validation; all downstream analyses relied on AUC scores as assessed based on this independent data. For analyses requiring binary classification of suitability predictions (for example, to assess spatial patterns of overpredictions and underpredictions across plots), we applied the tenth-percentile minimum training presence threshold.

To account for model-inherent errors (false presences or absences arising from statistical noise or sampling limitations), we used a paired-model framework in which each disequilibrium-informed SDM was directly compared with a corresponding non-disequilibrium-informed SDM built using identical data and parameters; only the relevant predictors were different. Because both models share the same structural assumptions and data limitations, model-inherent errors are effectively held constant, allowing changes in performance (ΔAUC) to reflect ecological mismatches rather than artefacts of model fit. Note, AUC values were logit-transformed before calculating deltas (Δlogit-AUC), which were used as measures of disequilibrium in downstream analyses.

To quantify the severity of distributional disequilibrium at the species level, we ran ten replicates for each modelling protocol and performed species-specific pairwise *t*-tests comparing the performance of standard SDMs against dynamic SDMs, disturbance-informed SDMs and dynamic, disturbance-informed SDMs (Δlogit-AUC). We applied the Benjamini–Hochberg correction to control for false discovery rate for each set of comparisons (*n* = 3,047) (for example, standard SDMs against dynamic SDMs). Species were classified as exhibiting significant climate change- or disturbance-driven disequilibrium when the relevant model significantly outperformed the baseline.

Between-protocol differences in model performance were tested using an LMM with logit-AUC as the response, modelling protocol as the fixed effect and species as the random intercept (ten replicates per species). Estimated marginal means were then used to obtain pairwise comparisons among modelling protocols with Tukey adjustment for multiple testing.

### Relationship between severity and attributes of species

To explore species-level correlates of disequilibrium severity, we modelled Δlogit-AUC values using LMMs with species attributes as predictors (Supplementary Section 3) and species as the random effect. Twenty attributes were selected after removing highly correlated variables (*r* ≥ 0.7) (Supplementary Table 4). These included: three data characteristics (total number of presence plots and median sampling year of training and testing presence plots), two geographic variables (latitude and elevation), three bioclimatic variables (annual mean temperature, mean diurnal range and isothermality), three disturbance-related predictors (community-averaged indicator values of light availability, disturbance severity and herb-layer disturbance severity) and nine plant functional traits (plant height, seed mass, seed number per reproduction unit, stem specific density, leaf dry matter content, specific leaf area, leaf area, leaf nitrogen and leaf nitrogen to phosphorus ratio). We also included a binary categorical predictor indicating if the other disequilibrium type was accounted for in the model; this tests if accounting for the other disequilibrium influenced the detectability of the focal disequilibrium type.

Data characteristics were included to assess potential data-related and temporal influences on disequilibrium detection. The number of presence plots, which was strongly correlated with estimated range size (*r* = 0.86), tested for possible data-dependence or range-size effects on disequilibrium detection. Median sampling years (training and testing) captured potential temporal biases in the plot data. These variables also allowed evaluation of whether species with more recent observations exhibited greater disequilibrium, which would reflect accelerating impacts of contemporary climate change.

Except for data characteristics and functional traits, predictor values were calculated as the median across presence plots for each species. Elevation data were extracted from EuroDEM 2023 (2 arcsec; retrieved 5 March 2024; www.mapsforeurope.org/datasets/euro-dem). Community-averaged indicator values were used over original species-level indicators because of broader data coverage (>250 species lacked original values). Where available, original and community-averaged values were highly correlated (light, *r* = 0.85; disturbance severity, *r* = 0.94; herb-layer disturbance severity, *r* = 0.96), supporting their use. Traits data were obtained from a gap-filled dataset compiled by the TRY initiative^61,62^. All predictors were standardized before analysis.

Quadratic terms were included for latitude, light availability, disturbance severity and herb-layer disturbance severity to test for potential unimodal relationships. Although we expected greater disequilibrium among species associated with open habitats, Bond’s concept of ecosystem uncertainty^16^ implies that disequilibrium should occur at both ends of the habitat spectrum—open and closed vegetation alike. Therefore, we included quadratic terms to allow for nonlinear, potentially symmetric responses across these gradients. These squared terms were uncorrelated with the other 20 predictors and each other, resulting in a final predictor set of 24 attributes.

Although we prefiltered highly correlated predictors, variance inflation factors (VIFs) remained elevated (Supplementary Section 3 and Supplementary Table 5). We therefore removed annual mean temperature (VIF = 31.1). Disturbance severity (VIF = 9.6) and light availability (VIF = 8.6) exceeded the strict threshold of 5, but we retained both because their VIFs remained below the less strict threshold of 10 and excluding them would hinder the interpretability of their quadratic terms. For completeness, we also explored alternative models with both of these variables removed (Supplementary Fig. 4). We also tested longitude to contextualize bioclimatic effects (for example, continentality). Longitude was moderately related with isothermality (VIF = 13.8), but excluding isothermality reduced VIFs to acceptable levels (longitude = 1.7) (Supplementary Table 5).

Although we used the same climate and disturbance covariates in our SDMs, we first collapsed them into independent species-level summaries (for example, the median value across all occurrence plots) and then used those summaries as predictors in LMMs for disequilibrium severity (Δlogit-AUC)—a response wholly separate from occurrence probability or habitat suitability—thus avoiding circularity.

Note, we chose not to rely solely on resampled data to analyse climate-change disequilibrium because of the unknown and highly variable spatial bias in the distribution of resurveyed plots (a smaller subset of the EVA plots used); both single-sampled and resampled plot data were used and treated similarly. Hence, we were unable to determine the type of climate-change disequilibrium occurring, that is, between extinction debts and colonization credits.

### Spatial distribution of disequilibrium

To identify where disequilibrium occurred along environmental gradients, we directly compared predictions of the test data between paired models—each dynamic and/or disturbance-informed SDM against its static climate and/or climate-only baseline. Disequilibrium plots were defined as cases where the baseline model misclassified presence or absence, but the disequilibrium-informed model predicted correctly. This approach isolates locations where accounting for disturbance or temporal climate dynamics resolves mismatches that static, climate-only assumptions cannot, thereby linking model improvement to ecological rather than statistical causes. For each species and selected environmental variables, we calculated median values across all presence plots and all disequilibrium plots. From the previously defined set of species attributes, we assessed patterns for the three disturbance-related predictors, four bioclimatic variables and three geographic variables. Cross-species patterns were visualized by plotting species-level medians (presence versus disequilibrium plots) and fitting GAMs to capture nonlinear trends. Analyses were restricted to species that exhibited significant disequilibrium (as assessed by the pairwise *t*-test and after adjusting for false discovery rate using the Benjamini–Hochberg) and at least five disequilibrium plots; because climate-change and disturbance-driven disequilibrium varied depending on whether the other was accounted for, significance tests and plot predictions were taken from models that included the other effect when it was significant. For overpredictions, this accounted for 3,015 species for disturbance-driven disequilibrium and 1,578 species for climate-change disequilibrium. For underpredictions, this accounted for 1,711 species for disturbance-driven disequilibrium and 590 species for climate-change disequilibrium. Variables shown in the main text were selected on the basis of statistical significance (severity–attribute relationship), ecological relevance or the emergence of distinct spatial patterns. Although both overpredictions and underpredictions were explored, we focused on the most relevant type for each disequilibrium; full results are presented in Extended Data Figs. 3 and 4.

### Analyses of lagged responses

We randomly selected 12 species with significant climate-change disequilibrium (*n* = 1,434) to illustrate variations in climate-window performances. Two species were selected for each combination growth form and range size. Plant growth forms were obtained from the TRY database and range size was estimated using an alpha-hull approach (Supplementary Section 3). Narrow-ranged species were defined as those below the 25th percentile (~175,782 km^2^) and widespread species as those above the 75th percentile (~1,336,012 km^2^) of alpha-hull range sizes, based on the distribution across species with significant climate-change disequilibrium. To confirm that observed patterns were not an artefact of this stratified selection, we also examined a fully random set of 24 species (Extended Data Fig. 5).

Best-performing climate windows were then grouped into three categories: recent (0–8 years before sampling), older (10–18 years) and null (static climate average over training and testing periods). For each species, the lag interval corresponding to its best-performing climate window was extracted and modelled as a response variable against species attributes, using the same modelling framework and considerations as for disequilibrium severity.

### Reporting summary

Further information on research design is available in the Nature Portfolio Reporting Summary linked to this article.

## Supplementary information

Supplementary Information Supplementary Tables 1–5, Figs. 1–13 and Methods (including Sections 1–3).

Reporting Summary

Peer Review File

## Data Availability

The vegetation plot data from the European Vegetation Archive (EVA), including plots from the ReSurveyEurope initiative that is comprised also of the LOTVS, GLORIA and forestREplot databases, belong to the owners or contributors of each vegetation dataset. The lead author has data use rights for the purpose of Project 189 ‘Rewilding’. Access to the vegetation data can be requested at http://euroveg.org/eva-database/obtaining-data. Climate data are openly available from the Climate Data Store at https://cds.climate.copernicus.eu/datasets/reanalysis-uerra-europe-single-levels?tab=download. Species indicator values are openly accessible from their respective articles^25,26^. The gap-filled dataset on plant functional traits was requested from the TRY Plant Trait Database together with the EVA data request under Project 189 ‘Rewilding’. Elevation data are openly available from EuroDEM 2023 at www.mapsforeurope.org/datasets/euro-dem.

## Code availability

Codes for developing temporally dynamic and disturbance-informed SDMs are available via GitHub at https://github.com/Pang-SEH/wildE_EU_Plant_Range_Disequilibrium, including codes used to make the figures in the main text, and via Zenodo at 10.5281/zenodo.20189873 (ref. ^63^). Additional computer code used in this study is available upon request from the corresponding author.
