# Supplementary material for: Disturbance regimes drive widespread plant range disequilibrium in Europe alongside climate change
Source: Nat Ecol Evol. 2026 Jul 7;10(8):1475–87. doi: 10.1038/s41559-026-03119-w (PMC13441915; doi:10.1038/s41559-026-03119-w)
Supplement: Supplementary file 1 [file 41559_2026_3119_MOESM1_ESM.pdf]

nature ecology & evolution

Supplementary information https://doi.org/10.1038/s41559-026-03119-w

Disturbance regimes drive widespread plant range disequilibrium in Europe alongside climate change

In the format provided by the authors and unedited

Disturbance regimes drive widespread plant range disequilibrium in Europe alongside climate change

Supplementary Information

Author list

Sean EH Pang*, Robert Buitenwerf*, Oliver Baines, Svetlana Aćić, Markus Bernhardt-Römermann, Idoia Biurrun, Gianmaria Bonari, Hans Henrik Bruun, Chaeho Byun, Eduardo Chacón-Madrigal, Alessandro Chiarucci, Anh Tuan Dang-Le, Pieter De Frenne, Arildo Dias, Jiri Dolezal, Emmanuel Garbolino, Behlül Güler, Nate Hough-Snee, Jens Kattge, Jonathan Lenoir, Adam R Martin, Sean T Michaletz, Vanessa Minden, Akira S Mori, Ülo Niinemets, Wolfgang Schmidt, Mária Šibíková, Grzegorz Swacha, Koenraad Van Meerbeek, Jens-Christian Svenning*

*Corresponding authors: SEHP (sean.pang@biology.ox.ac.uk); RB (buitenwerf@bio.au.dk); J-CS (svenning@bio.au.dk)

Table of Contents

Supplementary results 3

Supplementary Table 1. The percentage of studied species (n = 3,047) that exhibited significant disequilibrium for each combination of tests. 3

Supplementary Figure 1. Distribution of model performance assessed using TSS across the four model types 5

Supplementary Figure 2. As the single predictor in univariate models, the relationship between disturbance and climate-change disequilibrium severity and various species attributes 6

Supplementary Figure 3. In a variant model set-up with longitude replacing isothermality as a predictor, the relationship between disturbance and climate-change disequilibrium severity and various species attributes 7

Supplementary Figure 4. In a variant model set-up without light availability and disturbance severity as predictors, the relationship between disturbance and climate-change disequilibrium severity and various species attributes. 8

Supplementary Figure 5. The absolute number of (a) absence or (b) presence plots per species for which disequilibrium-informed SDMs predicted correctly but standard SDMs predicted incorrectly (y-axis) and vice-versa (x-axis). 9

Supplementary methods 10

Supplementary Section 1. Data preparation 10

Supplementary Figure 6. Distribution of the final, “usable”, post-thinning plot data across our study area of Europe (n = 525,638). 11

Supplementary Table 2. List of climate variables considered as predictors for the SDMs. 12

Supplementary Figure 7. Spatial distribution of absence plots used in this study, coloured by the plot-level mean light availability indicator value. 14
Supplementary Figure 8. Spatial distribution of absence plots used in this study, coloured by the plot-level mean disturbance severity indicator value............................15
Supplementary Figure 9. Spatial distribution of absence plots used in this study, coloured by the plot-level mean herb-layer disturbance severity indicator value. 16
Supplementary Table 3. Modelled relationships between plot-derived disturbance and light indicators and remotely sensed forest structural characteristics across spatial buffer sizes..17
Supplementary Section 2. Species distribution models 19
Supplementary Figure 10. Temporal change in the seven bioclimatic variables used as predictors for this study's SDMs...20
Supplementary Figure 11. Distribution of error rates across this study's 3,047 plant species 22
Supplementary Figure 12. Distribution of species-specific disturbance disequilibrium across 10 environmental gradients for (a) overpredictions and (b) underpredictions based on the maxSSS threshold. 23
Supplementary Figure 13. Distribution of species-specific climate-change disequilibrium across 10 environmental gradients for (a) overpredictions and (b) underpredictions based on the maxSSS threshold. 24
Supplementary Section 3. Severity and attributes 26
Supplementary Table 4. Full list of species attributes considered and those selected after excluding for highly correlated variables. 27
Supplementary Table 5. Full list of post-filtered predictors and their variance inflation factor (VIF) for various model variants. 28
References...30

Supplementary results

Supplementary Table 1. The percentage of studied species (n = 3,047) that exhibited significant disequilibrium for each combination of tests. Metric indicates the metric used to assess the difference in model performance, a proxy for disequilibrium severity, where model-inherent errors were assumed to remain constant between comparing models (same data): Area Under the Curve (AUC), True Skill Statistics (TSS), and Accuracy (Acc). Baseline SDM indicates the base SDM used for the point of comparison while the Disequi. SDM indicates the model the base SDM is being compared to (with one additional disequilibrium type accounted for): Standard SDM (SSDM); Temporally Dynamic SDM (TD-SDM); Light and Disturbance Informed SDM (LDI-SDM), Temporally Dynamic, Light and Disturbance Informed SDM (TD-LDI-SDM). Disequi. Tested indicates the type of disequilibrium being tested for in this comparison between models. Stats Test indicates the type of statistical test (paired) carried out: Student's t-test (t-test), Wilcoxon signed-rank test (Wilcoxon), and sign test (Sign test). Percentage Sig. indicates the percentage of species for which the disequilibrium tested was found significant for the given model performance metric—as the difference between stated baseline and disequilibrium SDM. The first four rows, in bold, indicate the results reported in the main text. All significance estimates were summarised after applying the Benjamini-Hochberg procedure to control for false discovery rate, applied per combination of tests (per row; *n* = 3,047). Additional performance metrics and statistical tests were included to confirm that the main results were robust to different performance measures and statistical assumptions.

Metric	Baseline SDM	Disequi. SDM	Disequi. Tested	Stats Test	Percentage Sig.
**AUC**	**SSDM**	**TD-SDM**	**Climate-change**	**t-test**	**49.29%**
**AUC**	**SSDM**	**LDI-SDM**	**Disturbance-driven**	**t-test**	**98.95%**
**AUC**	**LDI-SDM**	**TD-LDI-SDM**	**Climate-change**	**t-test**	**52.05%**
**AUC**	**TD-SDM**	**TD-LDI-SDM**	**Disturbance-driven**	**t-test**	**98.69%**
AUC	SSDM	TD-SDM	Climate-change	Wilcoxon	48.93%
AUC	SSDM	LDI-SDM	Disturbance-driven	Wilcoxon	98.92%
AUC	LDI-SDM	TD-LDI-SDM	Climate-change	Wilcoxon	51.66%
AUC	TD-SDM	TD-LDI-SDM	Disturbance-driven	Wilcoxon	98.65%
AUC	SSDM	TD-SDM	Climate-change	Sign test	47.06%
AUC	SSDM	LDI-SDM	Disturbance-driven	Sign test	98.88%
AUC	LDI-SDM	TD-LDI-SDM	Climate-change	Sign test	49.29%
AUC	TD-SDM	TD-LDI-SDM	Disturbance-driven	Sign test	98.49%
TSS	SSDM	TD-SDM	Climate-change	t-test	43.35%
TSS	SSDM	LDI-SDM	Disturbance-driven	t-test	95.44%
TSS	LDI-SDM	TD-LDI-SDM	Climate-change	t-test	41.35%
TSS	TD-SDM	TD-LDI-SDM	Disturbance-driven	t-test	94.68%
TSS	SSDM	TD-SDM	Climate-change	Wilcoxon	42.11%
TSS	SSDM	LDI-SDM	Disturbance-driven	Wilcoxon	95.11%
TSS	LDI-SDM	TD-LDI-SDM	Climate-change	Wilcoxon	40.04%
TSS	TD-SDM	TD-LDI-SDM	Disturbance-driven	Wilcoxon	94.42%
TSS	SSDM	TD-SDM	Climate-change	Sign test	39.97%
TSS	SSDM	LDI-SDM	Disturbance-driven	Sign test	94.62%
TSS	LDI-SDM	TD-LDI-SDM	Climate-change	Sign test	36.86%
TSS	TD-SDM	TD-LDI-SDM	Disturbance-driven	Sign test	93.60%
Acc	SSDM	TD-SDM	Climate-change	t-test	51.72%
Acc	SSDM	LDI-SDM	Disturbance-driven	t-test	96.06%
Acc	LDI-SDM	TD-LDI-SDM	Climate-change	t-test	54.32%
Acc	TD-SDM	TD-LDI-SDM	Disturbance-driven	t-test	95.41%
Acc	SSDM	TD-SDM	Climate-change	Wilcoxon	51.26%

Acc	SSDM	LDI-SDM	Disturbance-driven	Wilcoxon	95.77%
Acc	LDI-SDM	TD-LDI-SDM	Climate-change	Wilcoxon	52.31%
Acc	TD-SDM	TD-LDI-SDM	Disturbance-driven	Wilcoxon	95.24%
Acc	SSDM	TD-SDM	Climate-change	Sign test	47.72%
Acc	SSDM	LDI-SDM	Disturbance-driven	Sign test	95.44%
Acc	LDI-SDM	TD-LDI-SDM	Climate-change	Sign test	49.10%
Acc	TD-SDM	TD-LDI-SDM	Disturbance-driven	Sign test	94.81%

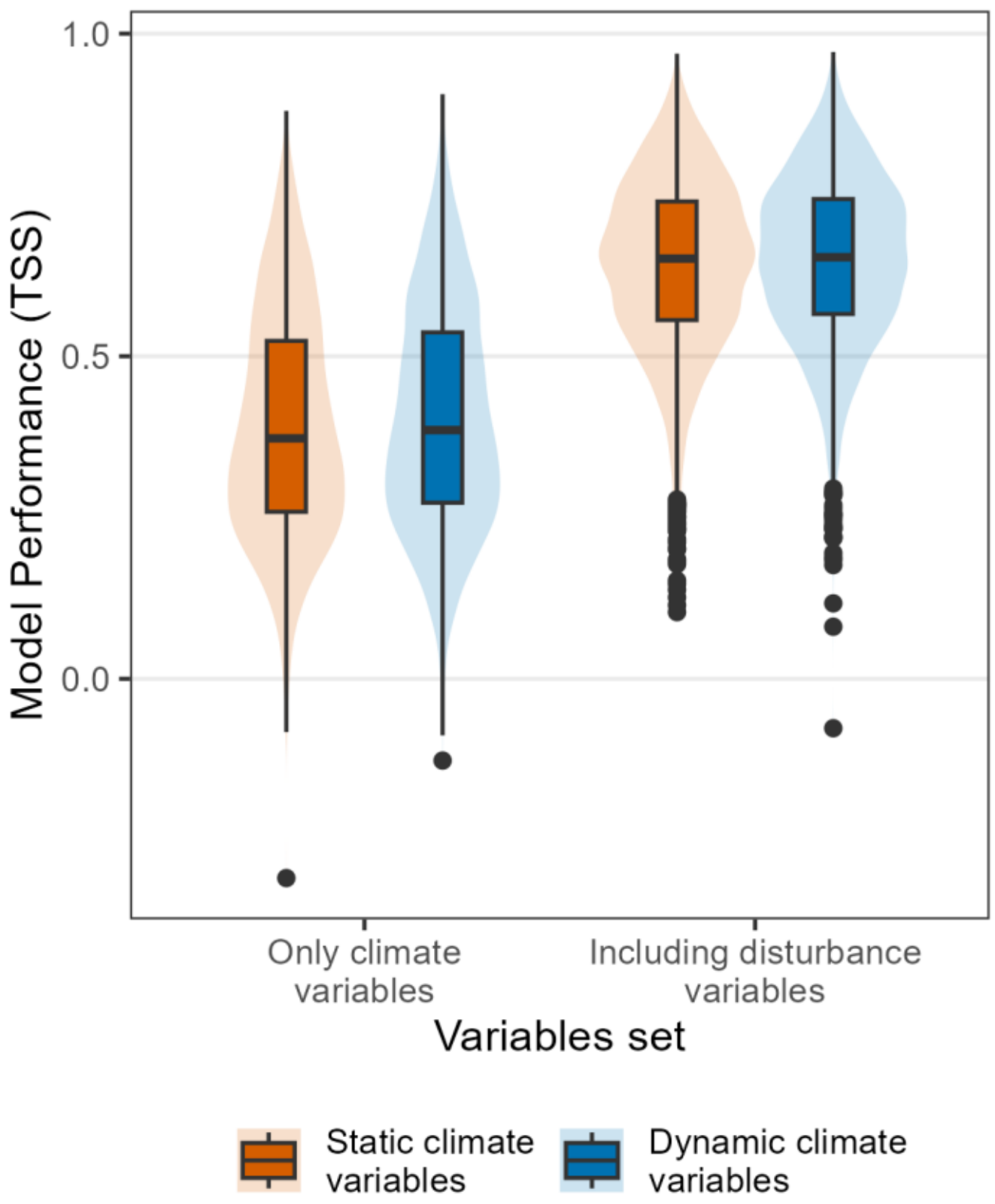

Supplementary Figure 1. Distribution of model performance assessed using TSS across the four model types. True skill statistic (TSS) is a discriminatory test of binary predictive performance, quantified as 1 minus the sum of sensitivity and specificity, and is another popular metric of SDM performance. Model performance, assessed using TSS, are shown for 3,047 European plant species across models differing in their use of climate variables (static vs. dynamic; colors) and the inclusion of disturbance variables (only climate vs. including disturbances; x-axis). Box-and-whisker plots show the median, spread, and variation in TSS across species. Static models used long-term climate averages, while dynamic models incorporated 5-year moving windows to capture lagged responses. Models including habitat predictors used community-derived indicators of light availability, disturbance severity, and herb-layer disturbance severity.

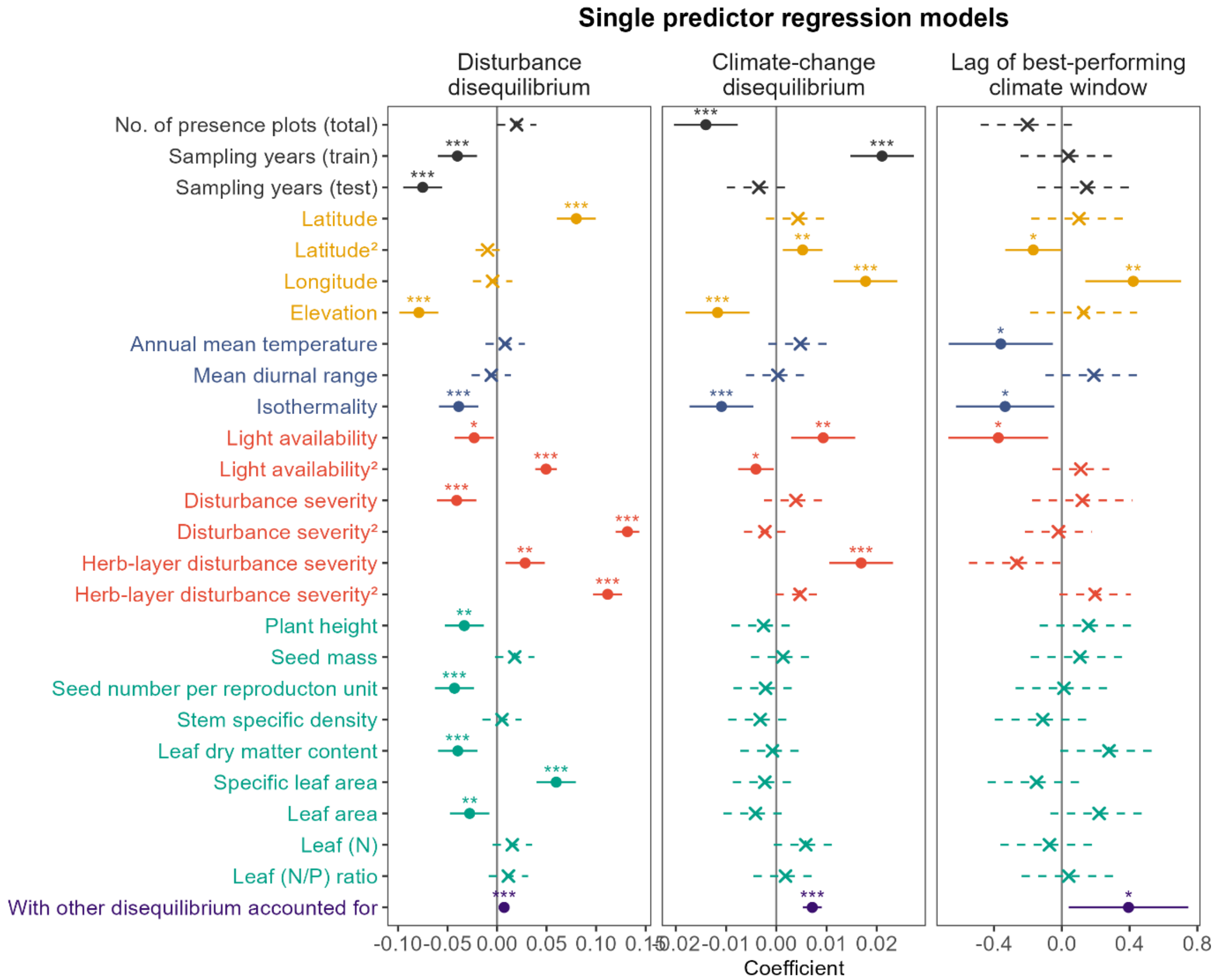

Supplementary Figure 2. As the single predictor in univariate models, the relationship between disturbance and climate-change disequilibrium severity and various species attributes. Generalized linear models (GLMs) were used to assess how various species attributes relate to disturbance (left) and climate-change (center) disequilibrium, measured as differences in logit-transformed AUC, for 2,606 European plant species with complete trait data, and to lag length (right) as defined by the best-performing window for 1,735 species with complete trait data and significant climate-change disequilibrium (829 species were significant for only one comparison—either between static and dynamic climate-only SDMs or between disturbance-informed static and dynamic SDMs). Species attributes assessed—from top to bottom—were data characteristics (black), geographic variables (gold), bioclimatic conditions (blue), disturbance-related indicators (orange), and plant functional traits (green), and if the other disequilibrium was accounted for when assessing the focal disequilibrium (purple). For all non-trait attributes, species-level median across presence plots was used. Points indicate model coefficients (solid for significant, cross for non-significant), 95% confidence intervals shown (solid lines for significant effects, dashed for non-significant). Significance levels are denoted by asterisks (* < 0.05, ** < 0.01, *** < 0.001).

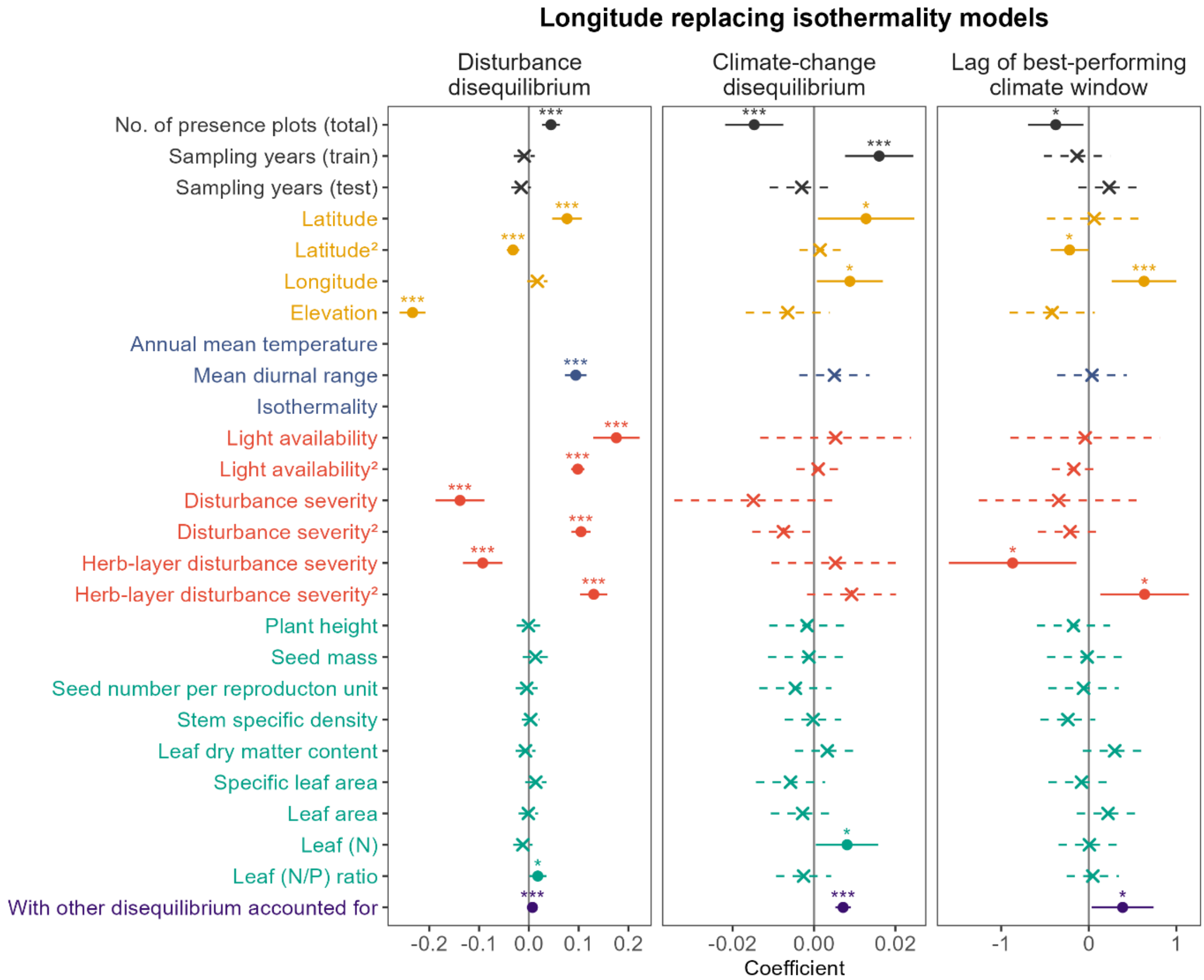

Supplementary Figure 3. In a variant model set-up with longitude replacing isothermality as a predictor, the relationship between disturbance and climate-change disequilibrium severity and various species attributes. Generalized linear models (GLMs) were used to assess how various species attributes relate to disturbance (left) and climate-change (center) disequilibrium, measured as differences in logit-transformed AUC, for 2,606 European plant species with complete trait data, and to lag length (right) as defined by the best-performing window for 1,735 species with complete trait data and significant climate-change disequilibrium (829 species were significant for only one comparison—either between static and dynamic climate-only SDMs or between disturbance-informed static and dynamic SDMs). Species attributes assessed—from top to bottom—were data characteristics (black), geographic variables (gold), bioclimatic conditions (blue), disturbance-related indicators (orange), and plant functional traits (green), and if the other disequilibrium was accounted for when assessing the focal disequilibrium (purple). For all non-trait attributes, species-level median across presence plots was used. Points indicate model coefficients (solid for significant, cross for non-significant), 95% confidence intervals shown (solid lines for significant effects, dashed for non-significant). Significance levels are denoted by asterisks (* < 0.05, ** < 0.01, *** < 0.001).

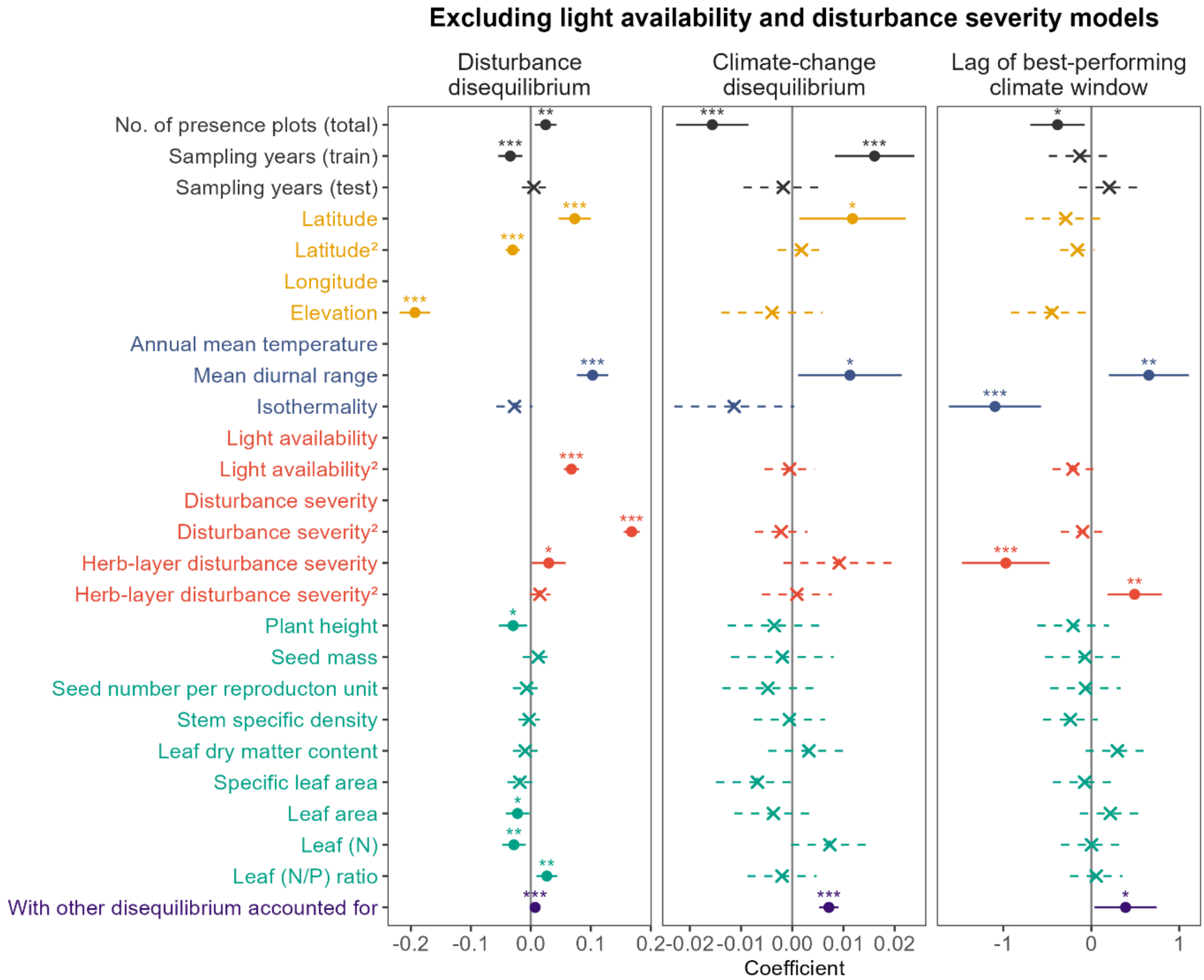

Supplementary Figure 4. In a variant model set-up without light availability and disturbance severity as predictors, the relationship between disturbance and climate-change disequilibrium severity and various species attributes. Generalized linear models (GLMs) were used to assess how various species attributes relate to disturbance (left) and climate-change (center) disequilibrium, measured as differences in logit-transformed AUC, for 2,606 European plant species with complete trait data, and to lag length (right) as defined by the best-performing window for 1,735 species with complete trait data and significant climate-change disequilibrium (829 species were significant for only one comparison—either between static and dynamic climate-only SDMs or between disturbance-informed static and dynamic SDMs). Species attributes assessed—from top to bottom—were data characteristics (black), geographic variables (gold), bioclimatic conditions (blue), disturbance-related indicators (orange), and plant functional traits (green), and if the other disequilibrium was accounted for when assessing the focal disequilibrium (purple). For all non-trait attributes, species-level median across presence plots was used. Points indicate model coefficients (solid for significant, cross for non-significant), 95% confidence intervals shown (solid lines for significant effects, dashed for non-significant). Significance levels are denoted by asterisks (* < 0.05, ** < 0.01, *** < 0.001).

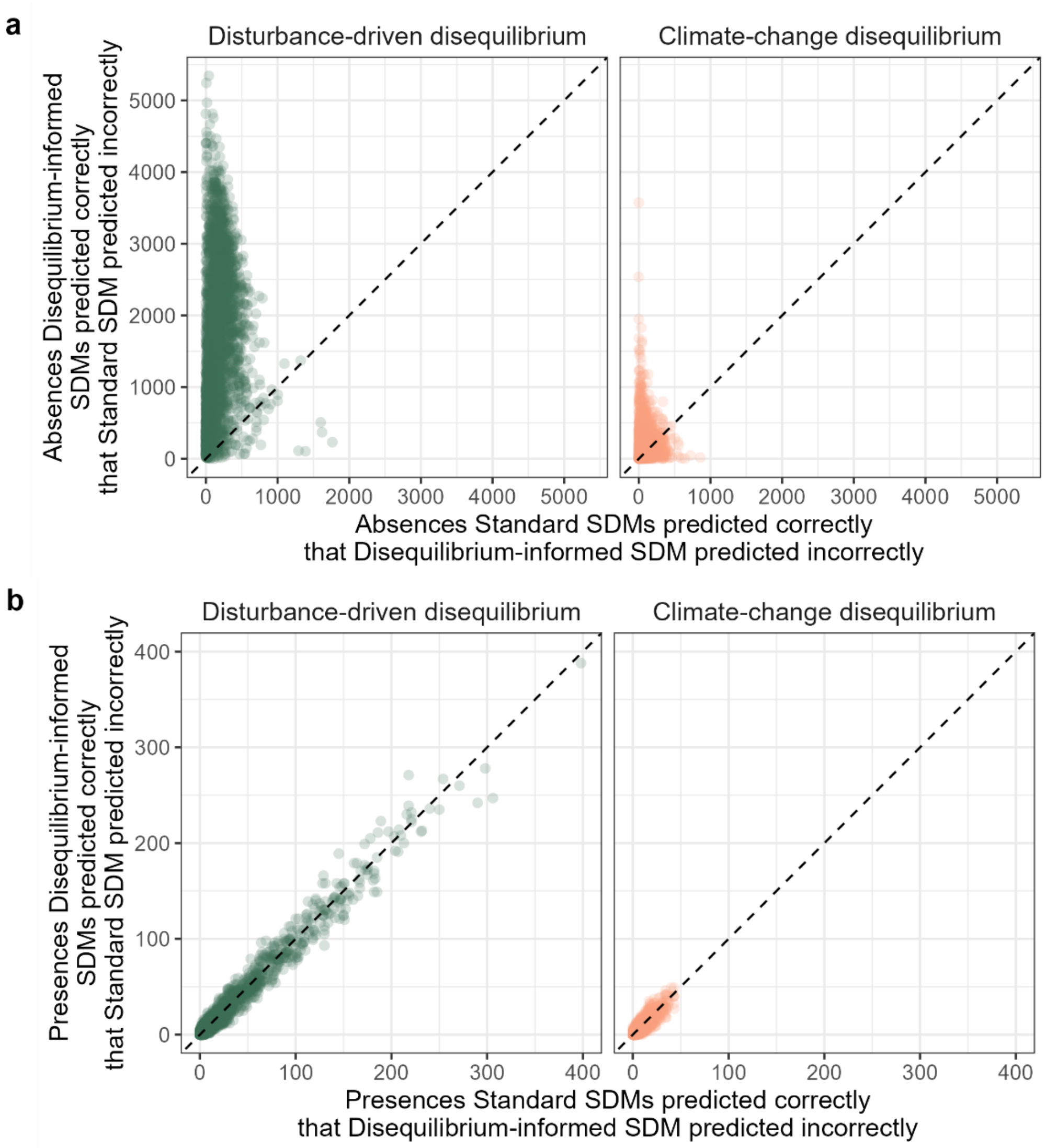

Supplementary Figure 5. The absolute number of (a) absence or (b) presence plots per species for which disequilibrium-informed SDMs predicted correctly but standard SDMs predicted incorrectly (y-axis) and vice-versa (x-axis). Because most of the points lay above the 1:1 line, disequilibrium-informed SDMs were systematically performing better than standard, non-disequilibrium SDMs, providing support that the occurrence of these resolved plots and their patterns across geographic space or environmental gradients were not stochastic or generated by random-between model variations.

Supplementary methods

Supplementary Section 1. Data preparation

After our filtering process of vegetation plots, 1,138,587 remain. Most vegetation plots were spatially aggregated or formed spatial clusters (spatially biased). To overcome spatial autocorrelation and to address these spatial biases, plots were thinned following protocols detailed in Supplementary Section 2. Over 1.1 million plots were considered, but 525,638 remained after thinning (presence plots here, absence thinning was done separately). We show the spatial distribution of the remaining 525,638 plots as they represent those that were directly used as presences to inform SDMs (Supplementary Fig. 6). Plot density was highest in Denmark, Netherlands, Czechia, the Alps including parts of Switzerland and eastern France, the Apennines in Italy, and the Pyrenees including northern Spain and southern France.

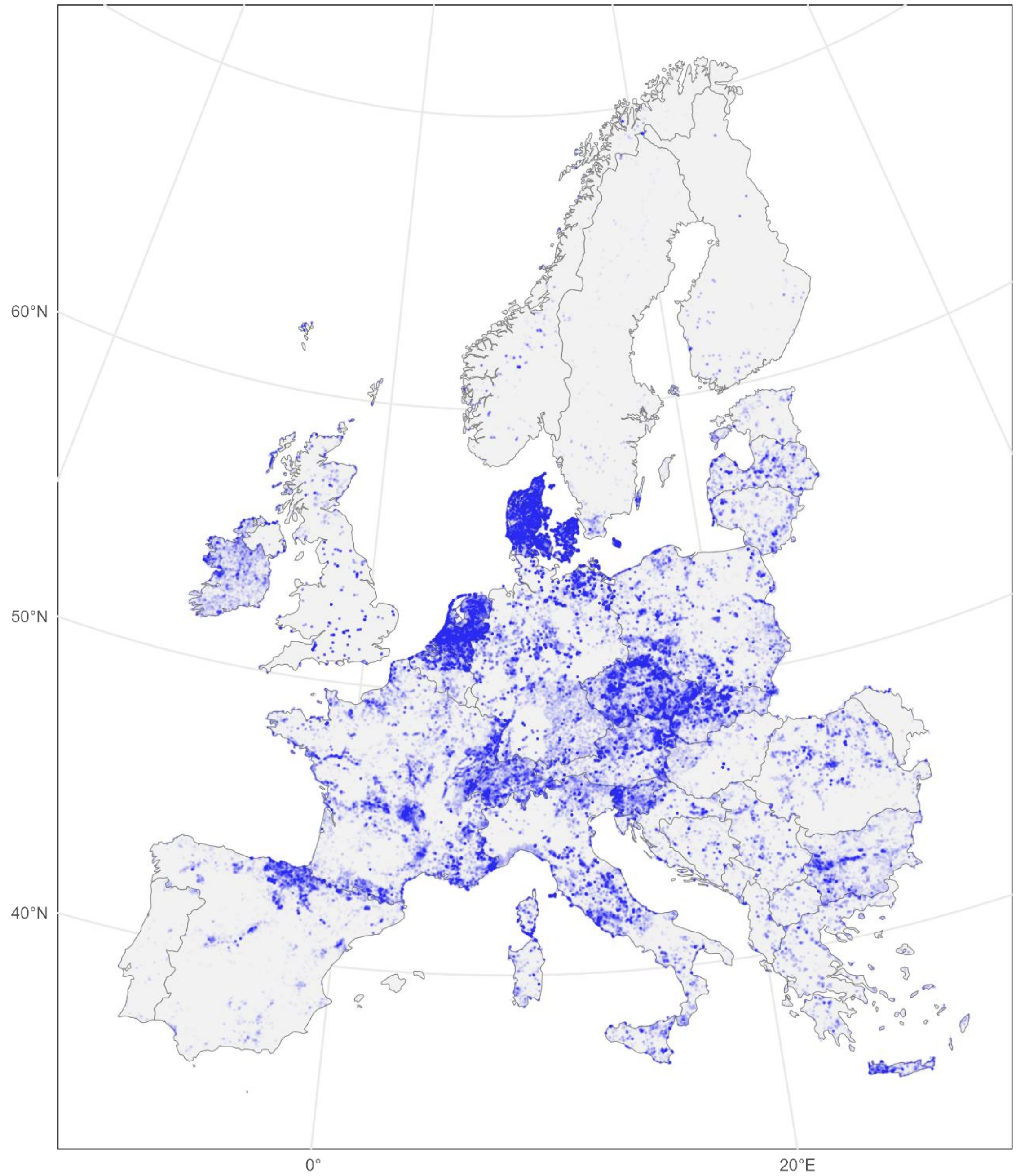

Supplementary Figure 6. Distribution of the final, "usable", post-thinning plot data across our study area of Europe (n = 525,638). Points were plotted with an opacity value of 0.01, where darker blue areas indicate a higher density of plots and light blue areas indicate a lower density of plots. Darker grey lines indicate country borders.

All climate data were sourced from the UERRA regional reanalysis for Europe dataset (retrieved 18th October 2023; Copernicus Climate Change Service 2019). Daily near-surface temperature and precipitation records from 1961 to 2018 were obtained from the MESCAN-SURFEX system (5.5 km by 5.5 km). Daily temperature data contained four measurements, at 00, 06, 12, and 18 UTC, which was used to first calculate daily mean, min, and max temperature. Daily measurements of mean, min, and max were then averaged to calculate monthly mean, min, and max temperatures. Meaning the average of those moments was calculated for the month, e.g., minimum monthly temperature was the minimum temperature observed for each day, averaged across days for a given month. Precipitation values only

contained one measurement per day, which was then summed across days within each month. These monthly variables were needed to calculate the bioclimatic variables considered. While several calculations allowed input data using daily climate variables to potentially calculate variables with greater accuracy (e.g., for calculating growing degree days), the temporal and spatial extent of our study limited our capacity to do so due to space and computational limitations.

A large selection of annual climate variables was considered. Namely, the commonly used 19 bioclimatic variables, and additional variables related to growth, snow, and frost. All variables were handled in R (R Core Team 2013) using the 'terra' (1.8-86) package (Hijmans et al. 2022). We accessed SAGA GIS (9.1.2) using the 'Rsagacmd' (0.4.3) package (Pawley 2019). The Climate and Weather Tools in SAGA GIS was used to calculate all bioclimatic variables (Conrad et al. 2015), namely: the bioclimatic variables based off the WorldClim – Global Climate Data project (Hijmans et al. 2005), growing degree days (Karger et al. 2017), tree growth season (Paulsen and Körner 2014, Karger et al. 2019), frost change frequency (Conrad et al. 2015), and snow cover (Paulsen and Körner 2014).

Precipitation variables based on temperature-defined periods, and vice versa, were excluded from the original 19 bioclimatic variables (four of these type of variables in total) due to spatial artifacts that typically arise from sudden changes in classification periods (months or quarters) resulting in jumps in values owing primarily to that change in classification period, not the actual climate conditions itself. Day specific growth-related variables were excluded as they were considered uninformative and non-applicable at continental scale development of SDMs, e.g., first growing degree day or first tree growing day; partly also because values are NA when all days of the year are growing days. The same applied to multiple snow- and frost-related variables, e.g., standard deviation of daily temperature span of frost change days. Hence, we pre-selected 21 annual climate variables, covering a wide range of climatic processes that are expected to drive plant distribution dynamics across Europe (Supplementary Table 2).

Supplementary Table 2. List of climate variables considered as predictors for the SDMs. Final predictors column indicates the final seven variables used as predictors for the SDMs after excluding highly correlated variables ($r \geq 0.7$).

**Predictors**	**Explanation**	**Final predictors (SDM)**
Annual mean temperature (bio 01)	Average temperature across the entire year	✓
Mean diurnal range (bio 02)	Average difference between monthly max and min temperatures	✓
Isothermality (bio 03)	Ratio of day–night temperature range to annual temperature range (bio 02 / bio 07) x 100	✓
Temperature seasonality (bio 04)	Variation in temperature over the year (standard deviation x 100)	✓
Max temperature of warmest month (bio 05)	Highest average monthly temperature	
Min temperature of coldest month (bio 06)	Lowest average monthly temperature	
Temperature annual range (bio 07)	Temperature range over the year (bio 05 - bio 06)	
Mean temperature of warmest quarter (bio 10)	Average temperature during the warmest 3-month period	
Mean temperature of coldest quarter (bio 11)	Average temperature during the coldest 3-month period	

Annual precipitation (bio 12)	Total rainfall (or equivalent) over the year	✓
Precipitation of wettest month (bio 13)	Amount of rainfall in the wettest month	
Precipitation of driest month (bio 14)	Amount of rainfall in the driest month	
Precipitation seasonality (bio 15)	Variation in monthly precipitation through the year (coefficient of variation)	✓
Precipitation of wettest quarter (bio 16)	Total rainfall during the wettest 3-month period	
Precipitation of driest quarter (bio 17)	Total rainfall during the driest 3-month period	
Growing degree days (>0°C)	Annual sum of daily mean temperatures above 0 °C	
Growing degree days (>5°C)	Annual sum of daily mean temperatures above 5 °C	
Growing degree days (>10°C)	Annual sum of daily mean temperatures above 10 °C	
Tree growth season length	Number of days suitable for tree growth (above growth temperature threshold); soil water capacity was not included in calculations	
Snow cover days	Number of days per year with snow cover	
Frost change frequency	Frequency of freeze–thaw transitions around 0 °C over the year	✓

Light indicator values were sourced from a harmonized dataset of Ellenberg-type values at the European scale (Tichý et al. 2023), using species aggregate values when available. These values reflect light conditions, or light availability, under which species are most typically found, ranging from deep shade to full sunlight (1–9).

Species disturbance indicator values for disturbance severity and herb-layer disturbance severity were obtained from the pan-European dataset of Midolo et al. (2023). Our definition of disturbance within the context of these indicators therefore follows Midolo et al. (2023), i.e., disturbance as the loss of plant community biomass (Grime 1979, White and Jentsch 2001). These indicators therefore quantify the mean proportion of above-ground plant biomass removed or destroyed by typical disturbance events in the habitats where each species occurs; at the herb-layer or community-level, where the community-level includes the herb-layer, such that herb-layer disturbance severity is necessarily less than or equal to (community-level) disturbance severity. These indicators indicate associated disturbance events, i.e., they do not measure specific disturbances or their impacts on above-ground plant biomass, but the severity of disturbances typically associated with the habitats that each species inhabits. In Midolo et al. (2023), habitat-level disturbance values were first estimated by experts for 236 EUNIS habitat types in the European Vegetation Archive, distinguishing between the whole community and the herb layer to capture differences in disturbance regimes among vegetation strata. Species-level indicator values were then calculated as the weighted average of these expert-based habitat values, using the number of vegetation plots in which a species occurred within each habitat as weights. Thus, a high disturbance-severity value represents species occurring most frequently in vegetation that typically experience major biomass loss (e.g., logging or bark beetle outbreak), whereas low values characterize species in habitats that typically experience low biomass loss (e.g., grazing or mowing); see disturbance habitat file from Midolo et al. (2023) (zenodo.org/records/7116957). These community- and herb-layer indicators together describe species' realized disturbance niches across Europe and were used as to calculate plot-level community-averages (as predictors for

the SDM) and species attributes in the Linear Mixed Models examining the relationship between disequilibrium severity and various species characteristics. We visualized the spatial distribution of these three disturbance-related plot variables using the final absence plots considered for all studied species (Supplementary Fig. 7, 8, 9).

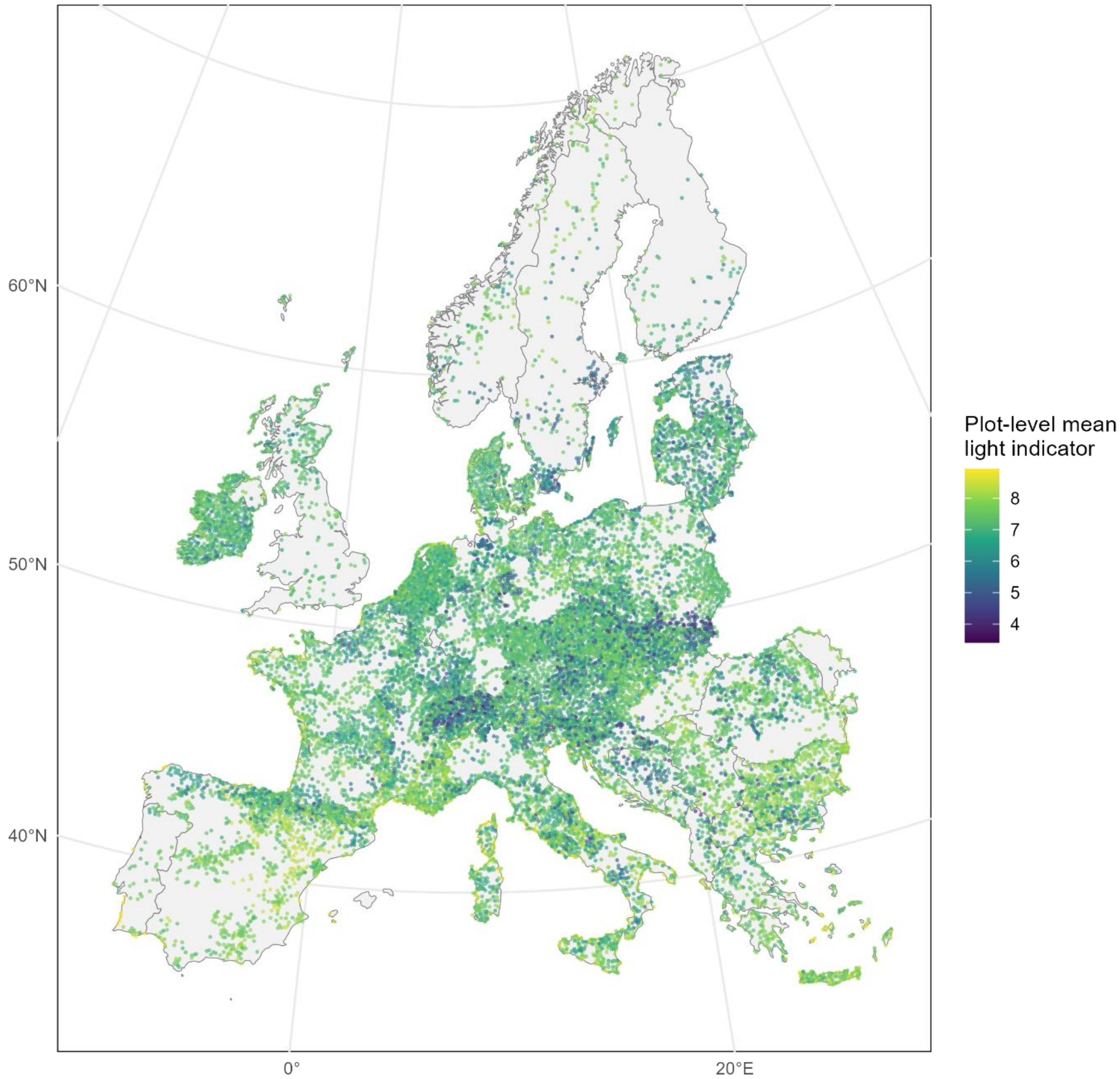

Supplementary Figure 7. Spatial distribution of absence plots used in this study, coloured by the plot-level mean light availability indicator value. Plot-level values were calculated as the unweighted mean of species' indicator values within each plot. Absence plots (*n* = 17,449) were shown because they represent the set of plots consistently used across species.

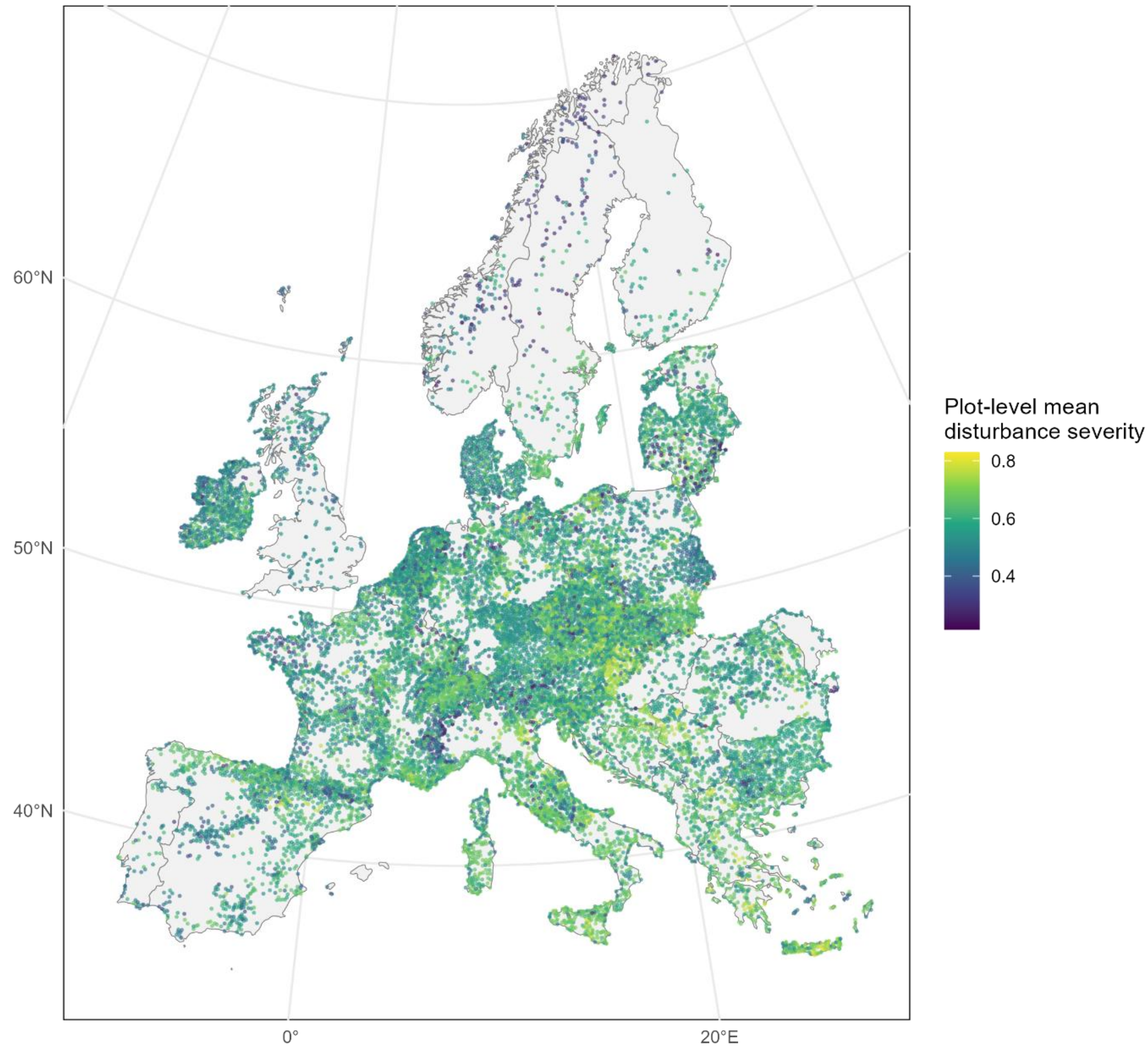

Supplementary Figure 8. Spatial distribution of absence plots used in this study, coloured by the plot-level mean disturbance severity indicator value. Plot-level values were calculated as the unweighted mean of species' indicator values within each plot. Absence plots (*n* = 17,449) were shown because they represent the set of plots consistently used across species.

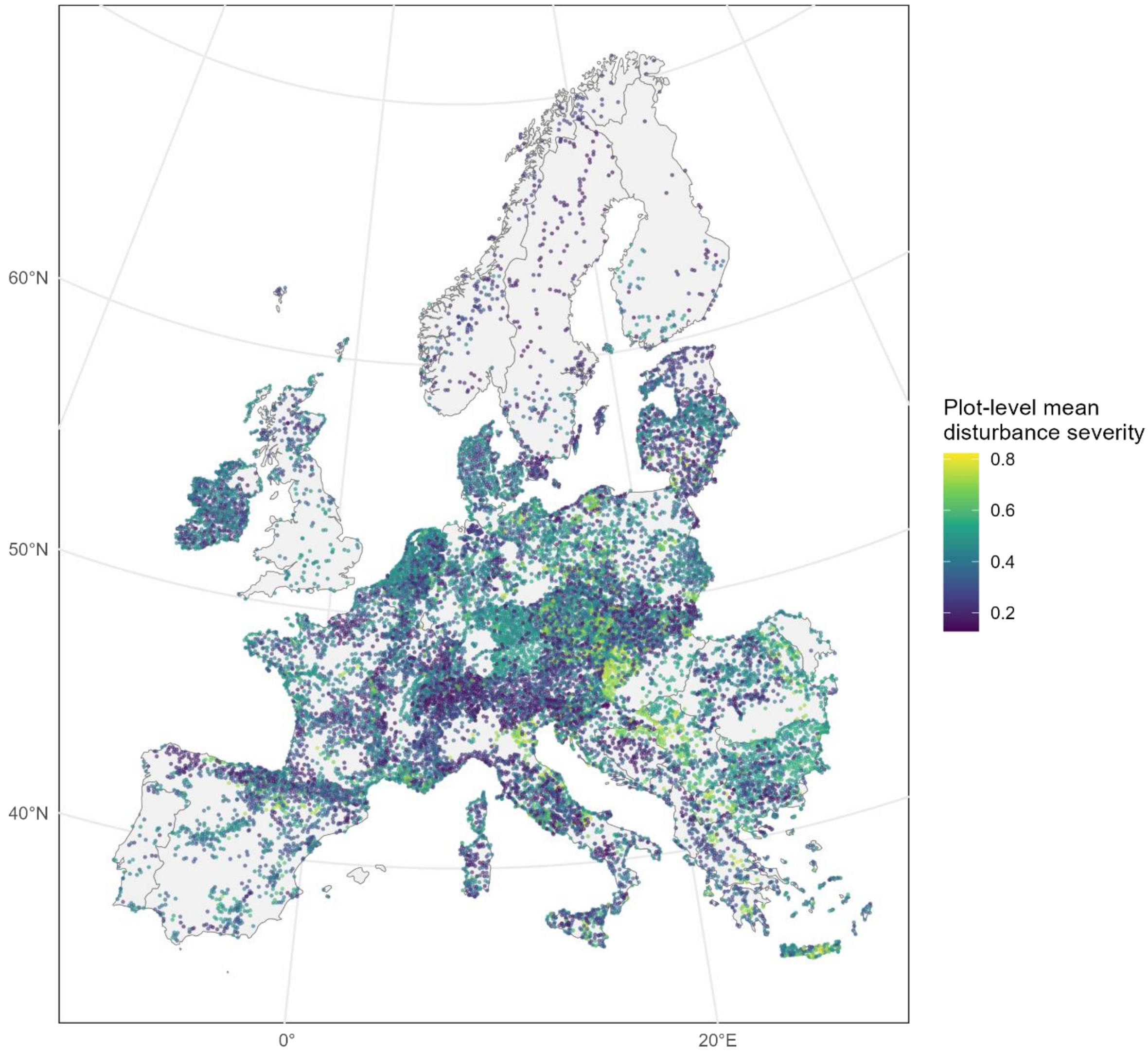

Supplementary Figure 9. Spatial distribution of absence plots used in this study, coloured by the plot-level mean herb-layer disturbance severity indicator value. Plot-level values were calculated as the unweighted mean of species' indicator values within each plot. Absence plots (n = 17,449) were shown because they represent the set of plots consistently used across species.

To provide an independent assessment of whether the disturbance and light indicators reflected ecologically meaningful forest structural conditions rather than solely internally cyclical compositional dynamics, we compared plot-level indicator values against remote sensing derived European tree extent and tree height products (0.00025° by 0.00025°) (Turubanova et al. 2023), which can be downloaded from https://glad.umd.edu/users/Potapov/Europe_TCH/Tree_Height/. Annual tree extent and tree height maps from 2001–2005 were averaged to produce composite structural layers, from which the mean and standard deviation of tree extent and tree height were extracted within circular buffers of 100, 500, and 1000 m around vegetation plot coordinates surveyed during the same period. Linear models relating remotely sensed structural metrics to plot-derived disturbance severity, herb-layer disturbance severity, and light availability showed that overall disturbance severity was generally only weakly associated with remotely sensed forest structure (Supplementary Table 3). In contrast, herb-layer disturbance severity and light availability showed comparatively stronger relationships with remotely sensed metrics, with

adjusted R^2 values generally declining with increasing buffer radius, indicating that these indicators primarily captured local-scale rather than broader landscape-scale vegetation conditions. Herb-layer disturbance severity was more consistently associated with tree extent metrics, whereas light availability was more consistently associated with tree height metrics, although the strength of relationships varied among spatial scales.

Supplementary Table 3. Modelled relationships between plot-derived disturbance and light indicators and remotely sensed forest structural characteristics across spatial buffer sizes. Linear models relating plot-level disturbance severity, herb-layer disturbance severity, and light availability indicators to remotely sensed tree extent and tree height metrics. Mean and standard deviation of remotely sensed tree extent and tree height were extracted within 100, 500, and 1000 m buffers surrounding vegetation plot coordinates. Reported values include model coefficients and their standard error, associated p-values, and model adjusted R^2. Bolded terms and reported values indicate significance within each model.

Indicator (Response)	Buffer scale (m)	Adjusted R^2	Remote sensing product (Term)	Estimate	Std Error	statistic	P-value
Disturbance Severity	100	0.05	(Intercept)	0.563	0.003	207.26	0.000
			Mean Tree Extent	0.017	0.017	1.00	0.318
			SD in Tree Extent	-0.019	0.028	-0.68	0.497
			**Mean Tree Height**	**0.003**	**0.001**	**2.69**	**0.007**
			SD in Tree Height	-0.002	0.003	-0.79	0.428
			**Mean Tree Extent : SD in Tree Extent**	**0.110**	**0.044**	**2.47**	**0.014**
			**Mean Tree Height : SD in Tree Height**	**0.000**	**0.000**	**-2.24**	**0.025**
	500	0.02	(Intercept)	0.560	0.004	138.14	0.000
			Mean Tree Extent	-0.013	0.046	-0.28	0.778
			SD in Tree Extent	0.036	0.032	1.12	0.264
			Mean Tree Height	0.005	0.003	1.87	0.062
			SD in Tree Height	-0.001	0.002	-0.62	0.533
			Mean Tree Extent : SD in Tree Extent	0.041	0.078	0.53	0.597
			Mean Tree Height : SD in Tree Height	0.000	0.000	-1.81	0.070
	1000	0.01	(Intercept)	0.559	0.005	114.67	0.000
			Mean Tree Extent	0.007	0.065	0.11	0.915
			SD in Tree Extent	0.030	0.035	0.85	0.398
			Mean Tree Height	0.004	0.004	1.11	0.266
			SD in Tree Height	0.000	0.002	0.07	0.941
			Mean Tree Extent : SD in Tree Extent	0.015	0.099	0.15	0.881
			Mean Tree Height : SD in Tree Height	-0.001	0.000	-1.68	0.092
Herb-layer Disturbance Severity	100	0.30	(Intercept)	0.462	0.004	126.87	0.000
			**Mean Tree Extent**	**-0.194**	**0.023**	**-8.33**	**0.000**
			**SD in Tree Extent**	**-0.141**	**0.037**	**-3.79**	**0.000**
			Mean Tree Height	-0.002	0.001	-1.37	0.170
			SD in Tree Height	0.005	0.003	1.49	0.137
			**Mean Tree Extent : SD in Tree Extent**	**0.106**	**0.060**	**1.78**	**0.075**
			Mean Tree Height : SD in Tree Height	0.000	0.000	0.41	0.685
	500	0.25	(Intercept)	0.476	0.006	85.53	0.000
			**Mean Tree Extent**	**-0.172**	**0.063**	**-2.74**	**0.006**
			**SD in Tree Extent**	**-0.300**	**0.044**	**-6.84**	**0.000**
			Mean Tree Height	-0.005	0.004	-1.23	0.217

			**SD in Tree Height**	**0.019**	**0.003**	**6.24**	**0.000**
			Mean Tree Extent : SD in Tree Extent	0.062	0.107	0.58	0.561
			Mean Tree Height : SD in Tree Height	0.000	0.000	-0.82	0.410
	1000	0.20	(Intercept)	0.478	0.007	69.47	0.000
			**Mean Tree Extent**	**-0.235**	**0.092**	**-2.54**	**0.011**
			**SD in Tree Extent**	**-0.416**	**0.050**	**-8.35**	**0.000**
			Mean Tree Height	-0.001	0.005	-0.14	0.891
			**SD in Tree Height**	**0.025**	**0.003**	**8.02**	**0.000**
			Mean Tree Extent : SD in Tree Extent	0.265	0.141	1.89	0.059
			**Mean Tree Height : SD in Tree Height**	**-0.001**	**0.000**	**-2.07**	**0.038**
Light availability	100	0.33	(Intercept)	7.452	0.021	355.70	0.000
			Mean Tree Extent	-0.130	0.134	-0.97	0.332
			SD in Tree Extent	0.213	0.214	1.00	0.319
			**Mean Tree Height**	**-0.069**	**0.008**	**-8.67**	**0.000**
			**SD in Tree Height**	**-0.077**	**0.019**	**-3.99**	**0.000**
			Mean Tree Extent : SD in Tree Extent	-0.116	0.343	-0.34	0.735
			**Mean Tree Height : SD in Tree Height**	**0.005**	**0.002**	**3.28**	**0.001**
	500	0.26	(Intercept)	7.566	0.032	234.05	0.000
			Mean Tree Extent	-0.365	0.365	-1.00	0.317
			SD in Tree Extent	-0.248	0.255	-0.97	0.332
			**Mean Tree Height**	**-0.070**	**0.022**	**-3.25**	**0.001**
			**SD in Tree Height**	**-0.067**	**0.017**	**-3.89**	**0.000**
			**Mean Tree Extent : SD in Tree Extent**	**1.255**	**0.621**	**2.02**	**0.043**
			Mean Tree Height : SD in Tree Height	0.003	0.002	1.54	0.124
	1000	0.21	(Intercept)	7.618	0.040	191.32	0.000
			**Mean Tree Extent**	**-1.744**	**0.534**	**-3.27**	**0.001**
			SD in Tree Extent	-0.542	0.289	-1.88	0.060
			Mean Tree Height	0.005	0.031	0.15	0.882
			**SD in Tree Height**	**-0.057**	**0.018**	**-3.21**	**0.001**
			**Mean Tree Extent : SD in Tree Extent**	**3.509**	**0.813**	**4.32**	**0.000**
			Mean Tree Height : SD in Tree Height	-0.003	0.003	-1.08	0.279

Supplementary Section 2. Species distribution models

We developed SDMs that explicitly accounted for disequilibrium dynamics to quantify the severity of range disequilibrium across species. Disequilibrium severity was measured as the change in model performance (ΔAUC) between disequilibrium-informed and standard static-climate SDMs, where higher improvements in predictive accuracy indicate stronger disequilibrium signals. AUC values were logit-transformed. The greater the increase in model performance, the greater the severity of disequilibrium is present.

To test for climate-change disequilibrium and lagged climate responses, vegetation plots were divided into two independent temporal bins: an earlier training period (1981–2003) and a later testing period (2004–2018). This design ensured temporal independence between model calibration and evaluation, thereby preventing temporal pseudo-replication and providing a biologically meaningful forward projection across a period of documented climatic change. A rough 20-year period was chosen for each temporal bin of data, which roughly translates to the common time period used for averaging climate values in other popular climate dataset, such as WorldClim (Fick and Hijmans 2017) or CHELSA climate data (Karger et al. 2017). These shorter, internally consistent bins also reduce temporal heterogeneity within each period (i.e., uneven sampling across years) while maintaining sufficient data coverage and spatial representativeness in both bins.

This temporal splitting framework also provides a conservative test for climate-change disequilibrium, as models are evaluated across a period where observed climatic change is significant but not extreme. This mirrors how SDMs are commonly applied to future climate projections, enabling evaluation of the consequences of assuming static equilibria during ongoing environmental change. We acknowledge that the temporal bins are necessarily broad and that species' equilibrium states likely adjust gradually rather than discretely between periods (Supplementary Fig. 10). Consequently, our framework represents a conservative test of climate-change and disturbance-driven disequilibrium, capturing detectable shifts within the observed period while recognising that finer temporal dynamics may remain unresolved. This design therefore yields robust, lower-frequency estimates of disequilibrium that reflect the dominant, measurable components of ongoing ecological change.

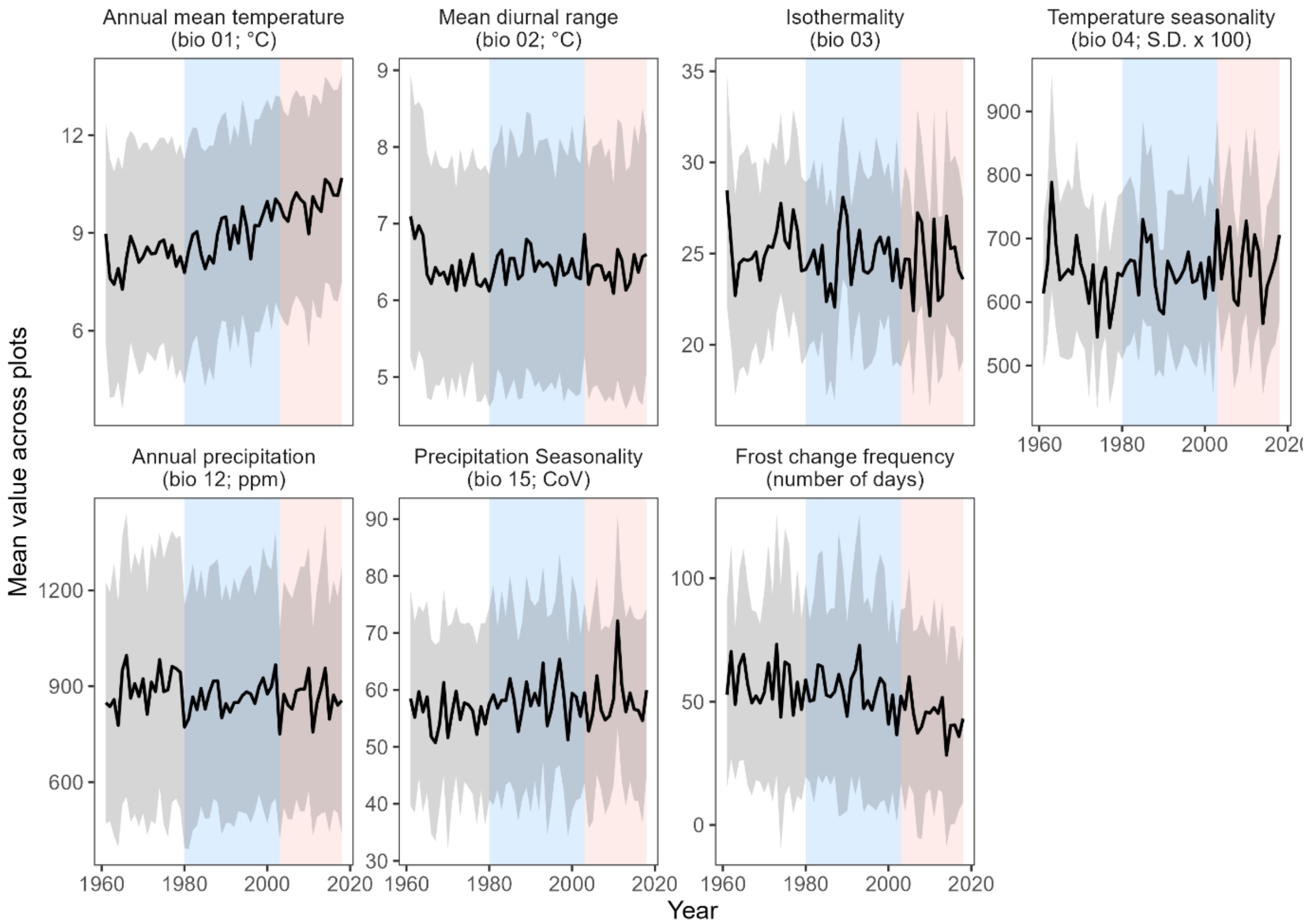

Supplementary Figure 10. Temporal change in the seven bioclimatic variables used as predictors for this study's SDMs. Solid lines represent the mean value for a given year while the shaded ribbons represent the standard deviation. Values were extracted from across absence plots as they represented the set of plots consistently used across the study's species. The blue shaded period (1981-2003) indicates the training period while the red shaded period (2004-2018) indicates the testing and validation period; note, climate variables used as predictors in dynamic SDMs do not necessarily align temporally with plots from a given period. We note a gradual increase in annual mean temperature (bio 01) during the training period (blue shade) that suggests potential ongoing climate change impact on plots recorded during this time period.

Species presence data were spatially thinned with a 10 km buffer to reduce autocorrelation, i.e., presences occurring within 10 km of another were removed, using the 'spThin' package in R (Aiello-Lammens et al. 2015). Thinning was applied 10 times (replicates), where the replicate with the greatest number of remaining presences, and then the most recent average year of sampling, was chosen. In this way, presence data was maximized while more recent sampling records were prioritized. The same was applied to each temporal bin. Absence data comprised plots without the target species and located within 300 km of its presence plots, a conservative threshold relative to common background sampling radii (typically up to 500 km). This distance helps minimise the inclusion of absences that are purely dispersal-limited—particularly those arising from long-term (>10,000 years) postglacial disequilibrium (Svenning and Skov 2004)—while retaining broad climatic and biogeographic representativeness across the continent. Absences were binned and thinned identically to presence plots.

We used 5-year moving averages of climate variables to represent recent climatic conditions influencing species distributions. This duration was chosen as a biologically and statistically pragmatic compromise. A 5-year window is sufficiently long to smooth interannual anomalies

(e.g., extreme single-year events) while still capturing short- to medium-term climatic variability relevant to plant distributional responses. For long-lived plant species, climatic influences on establishment and survival often integrate over several years, but longer decadal windows (e.g., 11 years) risk obscuring transient or lagged responses, especially for shorter-lived or disturbance-adapted species. Additionally, shorter windows increase the sensitivity of lag tests to detect climate–distribution relationships within coarse presence–absence data, which lack population or demographic information. Given these constraints, the 5-year window provided a pragmatic trade-off between biological realism, temporal resolution, and statistical detectability.

For analyses requiring binary classification of suitability predictions (e.g., to map spatial patterns of over- and underpredictions), we examined how threshold choice influences interpretation. Thresholding continuous suitability values inevitably introduces biases because it compresses model-specific calibration and ranking information into binary outcomes. Such thresholds therefore affect not only overall model accuracy but also how improvements between models manifest—as resolved presences or resolved absences. The focus of our study and analysis of spatial patterns was to identify where the disequilibrium-informed SDM resolved an error made by the baseline, static climate-only SDM (i.e., where disequilibrium occurred). In this context, the way thresholds trade off sensitivity and specificity directly determines which types of errors are considered "resolved."

We considered two widely used thresholds: the 10th-percentile minimum training presence (10mtp) and the maximum sum of sensitivity and specificity (maxSSS) (Supplementary Fig. 11). The 10mtp threshold is presence-driven—it fixes an initially high sensitivity off the training data and is therefore more likely to highlight reductions in commission errors (overpredictions), particularly where the disequilibrium-informed SDM lowers predicted suitability for climatically suitable plot that were unoccupied due to disturbance-related processes. In contrast, maxSSS seeks the point where the sum of sensitivity and specificity is maximised—that is, where the gain in one metric is no longer outweighed by the loss in the other. This optimisation inherently favours a balance between omission and commission errors. Hence, we adopted the 10mtp threshold for the main analyses because model overprediction was the expected dominant error type, particularly for disturbance-driven disequilibrium where we expect absences in climatically suitable but disturbance-wise unsuitable plots. Indeed, overpredicted plots remained more prevalent than underpredicted plots even when maxSSS was used (19% overprediction vs. 14% underprediction on average) (Supplementary Fig. 11). For climate-change-related disequilibrium, both over- and underprediction can occur, yet overall error rates were small and spatial signals weak, so we placed less emphasis on interpreting those patterns. To ensure robustness, we repeated the spatial analyses using the maxSSS threshold and also mapped both over- and underpredictions; the general spatial patterns were consistent between thresholds (Supplementary Figs. 12 and 13).

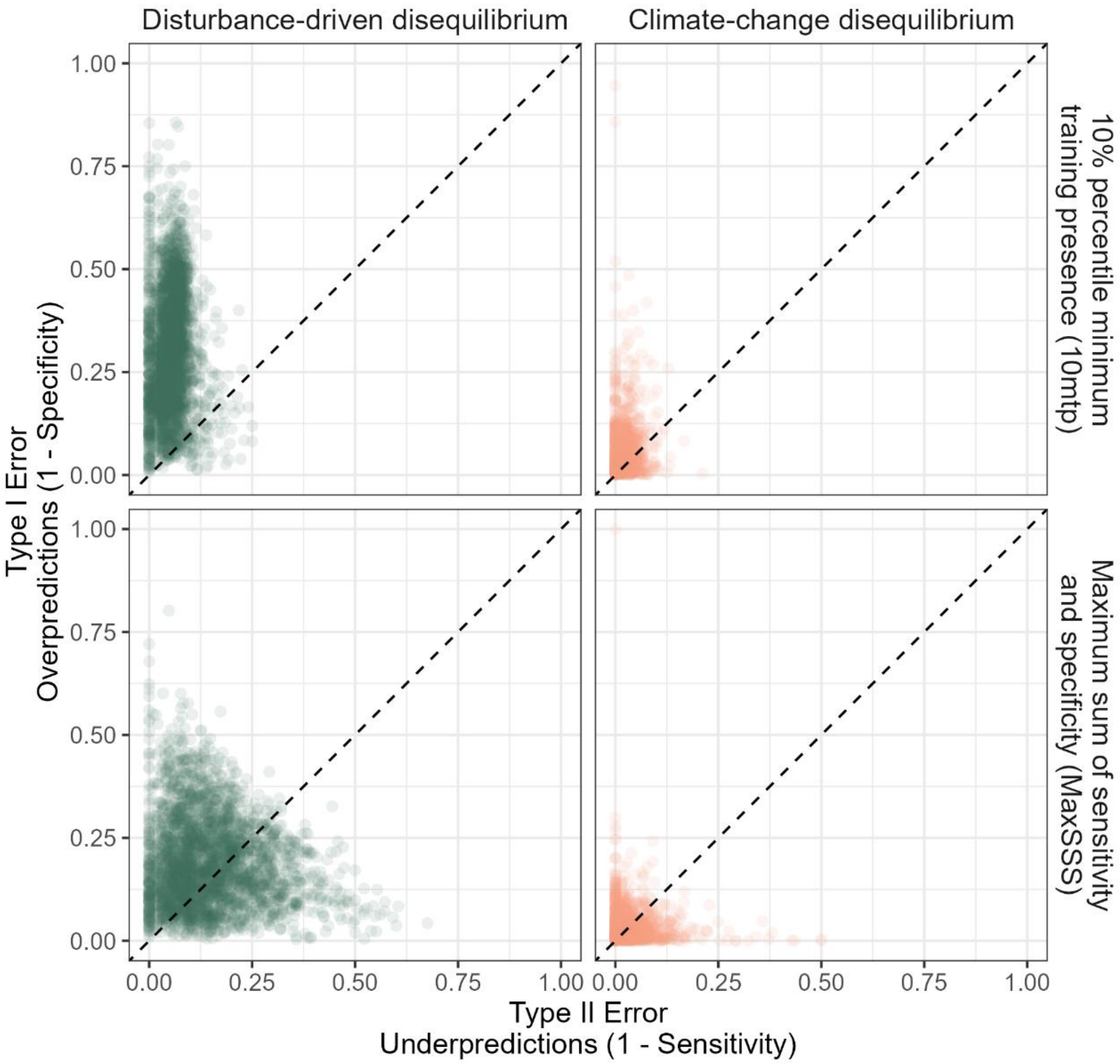

Supplementary Figure 11. Distribution of error rates across this study's 3,047 plant species: overpredictions (Type I error; 1 – Specificity) on the y-axis and underpredictions (Type II error; 1 – Sensitivity) on the x-axis. Distributions are shown separately for disturbance-driven and climate-change under two commonly used binary classification thresholds: the minimum 10th percentile minimum training presence (10mtp) and the maximum sum of sensitivity and specificity (MaxSSS) thresholds. Overpredictions represent plots where standard SDMs predicted presences, but where models incorporating either dynamic climate variables (climate-change disequilibrium) or disturbance-related predictor (disturbance-driven disequilibrium) correctly predicted absences, and vice versa for underpredictions. The 10mtp threshold, used in our main analyses, excludes marginally suitable sites (10th percentile) when predicting presences, and is a threshold insensitive to the predictions of suitability across absences. In contrast, the MaxSSS threshold minimizes total classification error but somewhat assumes equal error costs. This is problematic for disturbance disequilibrium, where absences often reflect omitted habitat conditions (e.g., light or disturbance regimes) not included in standard models, and likely have predicted suitability near-equal to those predicted for true presences. Consequently, by maximising sensitivity and specificity, MaxSSS balances false positives and negatives, masking the true extent and dominant error type pertaining to disturbance disequilibrium.

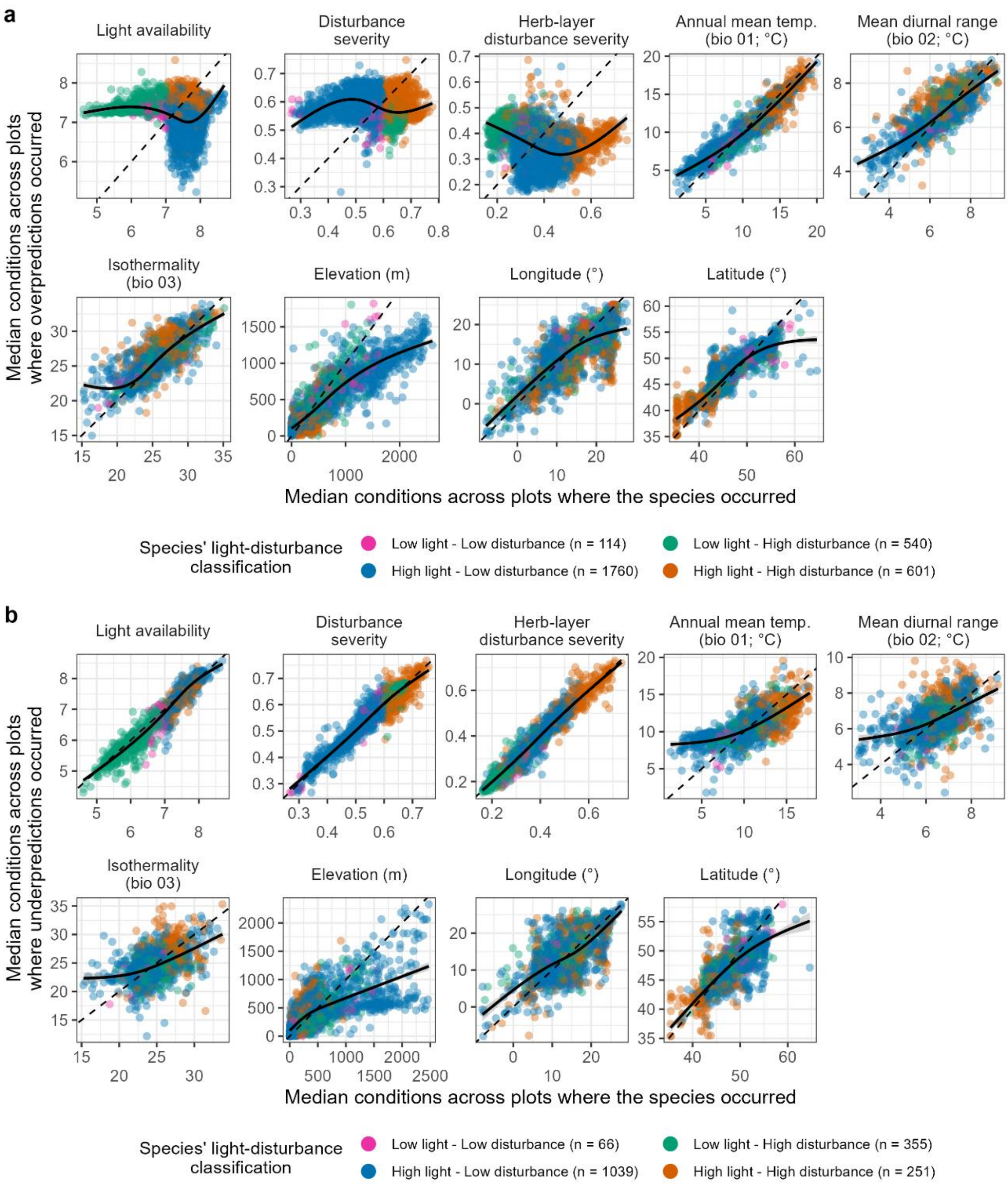

Supplementary Figure 12. Distribution of species-specific disturbance disequilibrium across 10 environmental gradients for (a) overpredictions and (b) underpredictions based on the maxSSS threshold. **(a)** Overpredictions are plots where standard SDMs predicted presences but disturbance-informed SDMs correctly predicted absences—indicating likely exclusion due to unsuitable disturbance conditions despite climatic suitability. **(b)** Underpredictions are plots where standard SDMs predicted absences but disturbance-informed SDMs correctly predicted presences—suggesting truncated climatic niches that became detectable when disturbance conditions were included. For each species, the median value of a given environmental variable across over-/underpredicted plots (y-axis) is plotted against the median across occupied plots (x-axis). The dashed 1:1 line indicates no difference in conditions; points above or below show whether over-/underpredictions occur in environments with higher or lower values, respectively. Generalized additive model (GAM) curves show across-species trends. Species were heuristically classified based on thresholds in light availability (7) and disturbance severity (0.6), determined where GAM lines crossed the 1:1 dashed line for analyses of overpredictions (a). Analyses included species with significant disturbance disequilibrium and at least five overpredicted (n = 3,015) or underpredicted (n = 1,711) plots. Because climate-change and disturbance-driven disequilibrium varied depending on whether the other was accounted for, significance estimates and plot predictions were taken from models that included the other effect when it was significant.

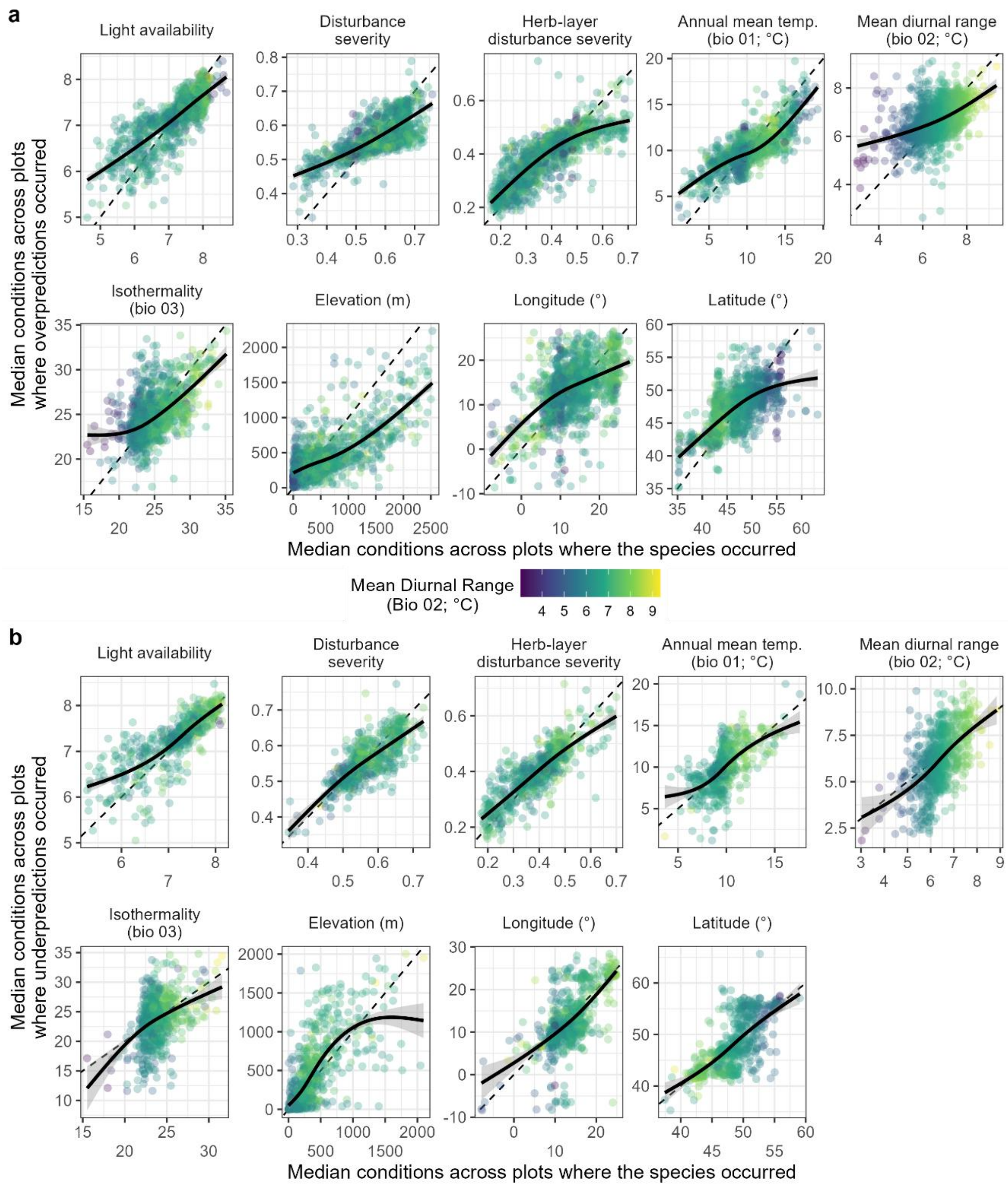

Supplementary Figure 13. Distribution of species-specific climate-change disequilibrium across 10 environmental gradients for (a) overpredictions and (b) underpredictions based on the maxSSS threshold. **(a)** Overpredictions are plots where standard SDMs predicted presences but dynamic SDMs correctly predicted absences—indicating where static variables lead to overestimations of suitability. **(b)** Underpredictions are plots where standard SDMs predicted absences but dynamic SDMs correctly predicted presences—indicating where excluding climate dynamics led to a truncation of the estimated climate niche. For each species, the median value of a given environmental variable across over-/underpredicted plots (y-axis) is plotted against the median across occupied plots (x-axis). The dashed 1:1 line indicates no difference in conditions; points above or below show whether over-/underpredictions occur in environments with higher or lower values, respectively. Generalized additive model (GAM) curves show across-species trends. Species were coloured based on the most important predictor of climate-change disequilibrium when analysing severity-attribute associations: temperature annual range. Analyses included species with significant climate-change disequilibrium and at least five overpredicted (*n* = 1,540) or underpredicted (*n* = 685) plots. Because climate-change and disturbance-driven disequilibrium varied depending on whether the other was accounted for, significance estimates and plot predictions were taken from models that included the other effect when it was significant.

Niche truncation, where data or models fail to capture the full environmental niche of a species and are forced to extrapolate beyond the observed environmental space, is a known limitation of SDMs. In Europe, truncation may arise from incomplete sampling of the species' climatic range, dispersal barriers linked to past biogeographic history, or anthropogenic constraints that prevent species from occupying otherwise suitable environments. While these processes can in principle contribute to apparent disequilibrium, many also overlap with it—disturbance-driven mismatches, land-use legacies, and habitat fragmentation all represent ecological forms of niche truncation, where parts of a species' potential niche and range are effectively excluded by non-climatic processes (Pang et al. 2022). In our framework, such patterns are treated as potential signals of disturbance-driven disequilibrium rather than artefacts, since they describe the realized restriction of species' niches under altered disturbance regimes. Broader biogeographically related niche truncation is less likely to influence our results because the EVA dataset encompasses most of the environmental and bioclimatic space occupied by European flora, including both Atlantic and continental extremes, and we evaluated model performance using only plots within the 300 km buffer limit. Moreover, models were not projected beyond the recent period (2004–2018), limiting temporal novelty and exposure to unobserved climatic conditions. Thus, while some degree of niche truncation may persist it is unlikely to bias our main results and instead represents an integral component of the disequilibrium processes we aimed to quantify.

Supplementary Section 3. Severity and attributes

To quantify the severity of distributional disequilibrium at the species level, we ran 10 replicates for each modelling protocol and performed species-specific pairwise t-tests comparing the performance of standard SDMs against dynamic SDMs, disturbance-informed SDMs, and dynamic, disturbance-informed SDMs (i.e., Δ logit-AUC). Note, logit-transformation was applied to AUC scores first before calculating their differences. Ecologically, higher Δ logit-AUC values indicate species for which ignoring disturbance or climate-change dynamics results in model underperformance—a signature of stronger disequilibrium between predicted and realized distributions. Conversely, near-zero or negative values suggest either quasi-equilibrium (equilibrium-like model and data behavior within the context of this study) or no consistent improvement from accounting for dynamic processes. While cases of negative values were rare for disturbance-informed SDMs, negative values were more common for dynamic SDMs. Negative values, however, do not indicate "negative disequilibrium", but that static climate conditions averaged over about two decades better predict species distributions than our tested form of dynamic climate variables. A negative value also does not necessarily mean that climate-change disequilibrium was not present, just that our tested form of dynamic SDMs did not detect it.

To explore potential species-level drivers of disequilibrium severity (Δ logit-AUC), we considered several species attributes, including those described in the main text. Several were excluded a prior to the pre-modelling correlation test because (a) they were not relevant to our hypothesis of where disequilibrium severity is likely greatest (e.g., stem conduit density and salinity indicator values) or (b) were found to exhibit high correlation with another variable early on (e.g., the number of presence plots, in total, within the training data bin, and within the testing data bin). Of relevance to the main text was estimated range size, which was highly correlated with the total number of presence plots (r = 0.86). Range size was estimated for each species using an alpha-hull approach applied to all unique geographic coordinates of vegetation plot occurrences (post data thinning but for both training and testing data). We implemented the *getDynamicAlphaHull* function from the 'rangeBuilder' package (v2.1) (Davis Rabosky et al. 2016) in R to construct species ranges, using a 90% inclusion fraction, a 10 km buffer, and a dynamic alpha parameter capped at 10. Hulls were clipped to terrestrial coastlines. Estimated range size was used when randomly selecting for narrow-ranged and widespread species for illustrating their climate-window performance (main text Fig. 4).

In total, five types of species attributes were considered.

1) **Data characteristics**—describe attributes of the available occurrence data for each species. These included the total number of presence plots and the median year of sampling for both training and testing data. Relationships with these attributes may indicate data-related biases, such as changes in sampling effort or representation over time.
2) **Geographical variables**—characterize the general distributional centroid of each species in geographic space, including median latitude, longitude, and elevation across all presence plots. These variables help capture broad-scale spatial tendencies that may relate to macroecological gradients or regional habitat types.
3) **Climate variables**—describe the median macroclimatic conditions where each species was observed. These were derived from the UERRA climate dataset and included annual mean temperature, mean diurnal range, isothermality, and temperature annual range—capturing

different dimensions of temperature variability and climatic regime. Note, precipitation variables were highly correlated with several geographical variables and excluded during the pre-modelling filtering step.

4) **Disturbance-related indicators**—reflect the median habitat conditions in which species occurred, based on community-derived indicator values of light availability, disturbance severity, and herb-layer disturbance severity. These values represent the realized vegetation structure and composition shaped by disturbance regimes (e.g., grazing, fire, clear-cutting), rather than direct measurements of disturbance events themselves. They serve as proxies for the ecological filtering effects of disturbance on species distributions.
5) **Plant functional traits**—include a suite of ecologically meaningful traits related to plant size, growth rate, tissue structure, and nutrient economy. These traits were obtained from a gap-filled version of the TRY database and they provide insight into species' life-history strategies and potential ecological tolerances.

Supplementary Table 4. Full list of species attributes considered and those selected after excluding for highly correlated variables. Five types of species attributes were considered. (1) Data characteristics that quantify attributes related to the data available for the species, where significant relationships with these attributes may indicate potential data-related biases. However, the total number of presence plots was highly correlated to estimated range size (r = 0.86), making it difficult to separate the effects of these two attributes. (2) Geographical type variables that describe the general distribution pattern of the species across geographic space. (3) Climate variables that describe the median climate conditions for which the species were found (presence plots). (4) Disturbance-related indicators reflect the general habitat conditions in which species were found, specific to indicators related to disturbance. Note, indicators here reflect the resulting vegetation structure and composition rather than direct measurements of disturbance processes. (5) Plant functional traits are a collection of ecologically relevant traits relating species' life-history strategies and potential ecological tolerances.

**Predictors**	**Attribute type**	**Post-filtering remaining predictors (severity ~ attribute GLM)**
No. of presence plots (total)	Data characteristic	✓
Sampling years (train)	Data characteristic	✓
Sampling years (test)	Data characteristic	✓
Latitude	Geographical	✓
Latitude^2	Geographical	✓
Longitude	Geographical	Considered later
Elevation	Geographical	✓
Annual mean temperature (bio 01)	Climatic	✓
Mean diurnal range (bio 02)	Climatic	✓
Isothermality (bio 03)	Climatic	✓
Temperature seasonality (bio 04)	Climatic	
Max temperature of warmest month (bio 05)	Climatic	
Min temperature of coldest month (bio 06)	Climatic	
Temperature annual range (bio 07)	Climatic	
Mean temperature of warmest quarter (bio 10)	Climatic	
Mean temperature of coldest quarter (bio 11)	Climatic	
Annual precipitation (bio 12)	Climatic	
Precipitation of wettest month (bio 13)	Climatic	
Precipitation of driest month (bio 14)	Climatic	
Precipitation seasonality (bio 15)	Climatic	
Precipitation of wettest quarter (bio 16)	Climatic	
Precipitation of driest quarter (bio 17)	Climatic	
Growing degree days (>0°C)	Climatic	
Growing degree days (>5°C)	Climatic	
Growing degree days (>10°C)	Climatic	
Tree growth season length	Climatic	
Snow cover days	Climatic	
Frost change frequency	Climatic	
Light availability	Disturbance-related indicator	✓

Light availability^2	Disturbance-related indicator	✓
Disturbance severity	Disturbance-related indicator	✓
Disturbance severity^2	Disturbance-related indicator	✓
Disturbance frequency	Disturbance-related indicator	
Herb-layer disturbance severity	Disturbance-related indicator	✓
Herb-layer disturbance severity^2	Disturbance-related indicator	✓
Herb-layer disturbance frequency	Disturbance-related indicator	
Plant height	Plant functional trait	✓
Seed mass	Plant functional trait	✓
Seed length	Plant functional trait	
Seed number per reproduction unit	Plant functional trait	✓
Dispersal unit length	Plant functional trait	
Stem specific density	Plant functional trait	✓
Leaf dry matter content	Plant functional trait	✓
Specific leaf area	Plant functional trait	✓
Leaf area	Plant functional trait	✓
Leaf fresh mass	Plant functional trait	
Leaf (N)	Plant functional trait	✓
Leaf (N/P) ratio	Plant functional trait	✓

After excluding highly correlated species attributes ($r \geq 0.7$), 23 attributes remained, including the quadratic terms for latitude, light availability, disturbance severity, and herb-layer disturbance severity. All 23 attributes were initially used as predictors in the generalized linear regression model, with disequilibrium severity (Δ logit-AUC) as the response variable and species as the random effect. However, even after pre-filtering for highly correlated predictors, model variance inflation factors (VIFs) were high. We therefore excluded the predictor with the highest VIF sequentially. However, for the main model, we decided to retain disturbance severity (VIF = 9.6) and light availability (VIF = 8.6). Despite their VIFs exceeding the stricter threshold of 5, those variables were retained as they were necessary for interpreting their quadratic terms. Additionally, VIFs for those two variables had not exceeded the less threshold of 10 and were thus within acceptable boundaries. However, as a precaution, we explored models for which light availability and disturbance severity were excluded. We also considered longitude, to potentially contextualise results related to bioclimatic variables (i.e., if it was related to continentality). Including longitude resulted in a high VIF for bio 03 or isothermality (VIF = 13.8); the VIF for longitude was reasonably low albeit above the strict threshold of 5 (VIF = 6.7). Excluding isothermality reduced VIF values to levels near the final model and for longitude (VIF = 1.7).

Supplementary Table 5. Full list of post-filtered predictors and their variance inflation factor (VIF) for various model variants. Values were rounded to one decimal place. Missing values indicate that the predictor was not included in this model variant (grey box). The column detailing the final set of predictors presented in the main text is bolded.

Predictor	Full model	Final model	Variant model (with longitude)	Variant model (with longitude excl. Isothermality)	Variant model (excl. disturbance severity)	Variant model (excl. disturbance severity and light)
**No. of presence plots (total)**	1.3	**1.3**	1.3	1.3	1.3	1.3
**Sampling years (train)**	1.6	**1.6**	1.8	1.8	1.6	1.5
**Sampling years (test)**	1.6	**1.6**	1.6	1.6	1.6	1.5
**Latitude**	13.5	**4.5**	6.7	3.6	3.0	2.7

**Latitude^2**	1.7	**1.7**	2.0	1.7	1.5	1.4
**Longitude**			6.7	1.7		
**Elevation**	19.4	**2.8**	2.8	2.7	2.6	2.5
**Annual mean temperature**	31.1					
**Mean diurnal range**	3.5	**2.6**	6.6	1.9	2.6	2.6
**Isothermality**	4.4	**3.5**	13.8		3.5	3.5
**Light availability**	10.6	**8.6**	9.0	8.8	7.2	
**Light availability^2**	2.6	**2.4**	2.5	2.4	2.4	2.0
**Disturbance severity**	10.8	**9.6**	9.7	9.6		
**Disturbance severity^2**	3.8	**3.5**	3.5	3.5	1.8	1.6
**Herb-layer disturbance severity**	6.4	**6.3**	6.3	6.3	6.1	3.0
**Herb-layer disturbance severity^2**	5.3	**5.2**	5.2	5.2	3.0	2.0
**Plant height**	2.2	**2.2**	2.2	2.2	2.2	2.1
**Seed mass**	2.6	**2.6**	2.6	2.6	2.6	2.6
**Seed number per reproduction unit**	2.0	**2.0**	2.0	2.0	2.0	2.0
**Stem specific density**	1.2	**1.2**	1.2	1.2	1.2	1.2
**Leaf dry matter content**	1.6	**1.6**	1.6	1.6	1.6	1.6
**Specific leaf area**	1.8	**1.8**	1.8	1.8	1.8	1.7
**Leaf area**	1.6	**1.6**	1.6	1.6	1.6	1.5
**Leaf (N)**	1.5	**1.5**	1.5	1.5	1.5	1.5
**Leaf (N/P) ratio**	1.2	**1.2**	1.2	1.2	1.2	1.2

When calculating disequilibrium severity (Δ logit-AUC) or identifying the spatial and environmental patterns where disequilibrium occurred (i.e., where predictions failed), we explicitly accounted for potential overlap between disturbance and climate-change disequilibrium. This step was critical to avoid confounding effects, ensuring that each type of disequilibrium was isolated and measured in a context where the other had already been addressed.

Specifically, when estimating the severity of climate-change disequilibrium, we did not always compare dynamic SDMs directly to standard static-climate-only SDMs. Instead, for species where disturbance disequilibrium was significant (n = 3,007 of 3,047), we used the disturbance-informed SDM as the baseline model. The comparison then tested the added benefit of incorporating dynamic climate variables on top of disturbance predictors—i.e., between disturbance-informed and dynamic-disturbance-informed SDMs. This isolates the effect of temporal climatic mismatch (lagged responses), controlling for any model performance improvement attributable to habitat filtering from disturbance effects.

A reciprocal procedure was applied when assessing disturbance disequilibrium. For the subset of species with significant climate-change disequilibrium (n = 1,586 of 3,047), we compared the dynamic-disturbance-informed SDM (which included both climate lags and disturbance predictors) against the dynamic SDM (accounting for lagged climate responses). For species

without significant climate-change disequilibrium, comparisons defaulted to the standard SDM.

This model-nesting approach (e.g., disturbance → disturbance + climate change; or climate → climate + disturbance) ensures that each form of disequilibrium was evaluated incrementally, using the most appropriate baseline for each species where appropriate. We did this for two reasons: (1) it avoided overestimating disequilibrium severity due to model misspecification or omission of a dominant filtering process, and (2) it allowed for a cleaner ecological interpretation—i.e., that the quantified disequilibrium truly reflects the respective process (lagged climate response or habitat filtering from disturbance), not a composite effect of both (i.e., one disequilibrium being attributed to another). We considered this approach mainly because early results suggested a marked decrease in effect size when including dynamic climate variables for models that had already incorporated disturbance-related predictors—which we demonstrate in the main text. This design therefore strengthened inference by ensuring that (a) Δ logit-AUC captures the marginal contribution of either climate or disturbance, (b) analyses of over- and underpredictions reflect process-specific mismatches in predicted vs. realized distributions, and (c) spatial and environmental patterns in disequilibrium are not misattributed to the wrong underlying driver.

References

Aiello-Lammens, M. E., Boria, R. A., Radosavljevic, A., Vilela, B. and Anderson, R. P. 2015. spThin: an R package for spatial thinning of species occurrence records for use in ecological niche models. - Ecography 38: 541–545.

Conrad, O., Bechtel, B., Bock, M., Dietrich, H., Fischer, E., Gerlitz, L., Wehberg, J., Wichmann, V. and Böhner, J. 2015. System for Automated Geoscientific Analyses (SAGA) v. 2.1.4. - Geosci. Model Dev. 8: 1991–2007.

Copernicus Climate Change Service 2019. UERRA regional reanalysis for Europe on single levels from 1961 to 2019.

Davis Rabosky, A. R., Cox, C. L., Rabosky, D. L., Title, P. O., Holmes, I. A., Feldman, A. and McGuire, J. A. 2016. Coral snakes predict the evolution of mimicry across New World snakes. - Nat Commun 7: 11484.

Fick, S. E. and Hijmans, R. J. 2017. WorldClim 2: new 1-km spatial resolution climate surfaces for global land areas. - International Journal of Climatology 37: 4302–4315.

Grime, J. P. 1979. Plant strategies and vegetation processes. - John Wiley & Sons, Limited.

Hijmans, R. J., Cameron, S. E., Parra, J. L., Jones, P. G. and Jarvis, A. 2005. Very high resolution interpolated climate surfaces for global land areas. - Int. J. Climatol. 25: 1965–1978.

Hijmans, R. J., Bivand, R., van Etten, J., Forner, K., Ooms, J. and Pebesma, E. 2022. Package ‘terra.’ in press.

Karger, D. N., Conrad, O., Böhner, J., Kawohl, T., Kreft, H., Soria-Auza, R. W., Zimmermann, N. E., Linder, H. P. and Kessler, M. 2017. Climatologies at high resolution for the earth's land surface areas. - Sci Data 4: 170122.

Karger, D. N., Kessler, M., Conrad, O., Weigelt, P., Kreft, H., König, C. and Zimmermann, N. E. 2019. Why tree lines are lower on islands—Climatic and biogeographic effects hold the answer. - Global Ecology and Biogeography 28: 839–850.

Midolo, G., Herben, T., Axmanová, I., Marcenò, C., Pätsch, R., Bruelheide, H., Karger, D. N., Aćić, S., Bergamini, A., Bergmeier, E., Biurrun, I., Bonari, G., Čarni, A., Chiarucci, A., De Sanctis, M., Demina, O., Dengler, J., Dziuba, T., Fanelli, G., Garbolino, E., Giusso Del Galdo, G., Goral, F., Güler, B., Hinojos-Mendoza, G., Jansen, F., Jiménez-Alfaro, B., Lengyel, A., Lenoir, J., Pérez-Haase, A., Pielech, R., Prokhorov, V., Rašomavičius, V., Ruprecht, E., Rūsiņa, S., Šilc, U., Škvorc, Ž., Stančić, Z., Tatarenko, I. and Chytrý, M. 2023. Disturbance indicator values for European plants. - Glob. Ecol. Biogeogr. 32: 24–34.

Pang, S. E. H., Zeng, Y., De Alban, J. D. T. and Webb, E. L. 2022. Occurrence–habitat mismatching and niche truncation when modelling distributions affected by anthropogenic range contractions (B Leroy, Ed.). - Divers Distrib 28: 1327–1343.

Paulsen, J. and Körner, C. 2014. A climate-based model to predict potential treeline position around the globe. - Alp Botany 124: 1–12.

Pawley, S. 2019. Rsagacmd: Linking R with the Open-Source "SAGA-GIS" Software.: 0.4.3.

R Core Team 2013. R: A language and environment for statistical computing. - R Foundation for Statistical Computing, Vienna, Austria 55: 275–286.

Svenning, J. and Skov, F. 2004. Limited filling of the potential range in European tree species. - Ecology Letters 7: 565–573.

Tichý, L., Axmanová, I., Dengler, J., Guarino, R., Chytrý, M., et al. 2023. Ellenberg-type indicator values for European vascular plant species. - J Veg Sci in press.

Turubanova, S., Potapov, P., Hansen, M. C., Li, X., Tyukavina, A., Pickens, A. H., Hernandez-Serna, A., Arranz, A. P., Guerra-Hernandez, J., Senf, C., Häme, T., Valbuena, R., Eklundh, L., Brovkina, O., Navrátilová, B., Novotný, J., Harris, N. and Stolle, F. 2023. Tree canopy extent and height change in Europe, 2001–2021, quantified using Landsat data archive. - Remote Sensing of Environment 298: 113797.

White, P. S. and Jentsch, A. 2001. The Search for Generality in Studies of Disturbance and Ecosystem Dynamics. - In: Esser, K. et al. (eds), Progress in Botany: Genetics Physiology Systematics Ecology. Springer, pp. 399–450.
