# Supplementary material for: Disturbance regimes drive widespread plant range disequilibrium in Europe alongside climate change
Source: Nat Ecol Evol. 2026 Jul 7;10(8):1475–87. doi: 10.1038/s41559-026-03119-w (PMC13441915; doi:10.1038/s41559-026-03119-w)
Supplement: Supplementary file 2 [file 41559_2026_3119_MOESM2_ESM.pdf]

nature portfolio

Corresponding author(s): Sean E. H. Pang, Robert Buitenwerf, Jens-Christian Svenning

Last updated by author(s): May 14, 2026

Reporting Summary

Nature Portfolio wishes to improve the reproducibility of the work that we publish. This form provides structure for consistency and transparency in reporting. For further information on Nature Portfolio policies, see our Editorial Policies and the Editorial Policy Checklist.

Statistics

For all statistical analyses, confirm that the following items are present in the figure legend, table legend, main text, or Methods section.

n/a	Confirmed	
☐	☒	The exact sample size (*n*) for each experimental group/condition, given as a discrete number and unit of measurement
☐	☒	A statement on whether measurements were taken from distinct samples or whether the same sample was measured repeatedly
☐	☒	The statistical test(s) used AND whether they are one- or two-sided<br>*Only common tests should be described solely by name; describe more complex techniques in the Methods section.*
☐	☒	A description of all covariates tested
☐	☒	A description of any assumptions or corrections, such as tests of normality and adjustment for multiple comparisons
☐	☒	A full description of the statistical parameters including central tendency (e.g. means) or other basic estimates (e.g. regression coefficient) AND variation (e.g. standard deviation) or associated estimates of uncertainty (e.g. confidence intervals)
☐	☒	For null hypothesis testing, the test statistic (e.g. *F*, *t*, *r*) with confidence intervals, effect sizes, degrees of freedom and *P* value noted<br>*Give P values as exact values whenever suitable.*
☒	☐	For Bayesian analysis, information on the choice of priors and Markov chain Monte Carlo settings
☒	☐	For hierarchical and complex designs, identification of the appropriate level for tests and full reporting of outcomes
☒	☐	Estimates of effect sizes (e.g. Cohen's *d*, Pearson's *r*), indicating how they were calculated

Our web collection on statistics for biologists contains articles on many of the points above.

Software and code

Policy information about availability of computer code

Data collection	No software was used for data collection. Data were all obtained from existing repositories, data archives, or were open source datasets.
Data analysis	SAGA GIS (9.1.2) software<br>R version 4.5.2<br>'terra' (1.8-86) R package<br>'Rsagacmd' (0.4.3) R package<br>'spThin' (0.2.0) R package<br>'rangeBuilder' (2.1) R package<br>'mgcv' (1.9.0) R package<br>'lme4' (1.1-37) R package<br>Custom code for developing annual climate variables (available on request)<br>Custom code for fitting temporally dynamic SDMs and disturbance-informed SDMs (available on GitHub; see Code Availability)

For manuscripts utilizing custom algorithms or software that are central to the research but not yet described in published literature, software must be made available to editors and reviewers. We strongly encourage code deposition in a community repository (e.g. GitHub). See the Nature Portfolio guidelines for submitting code & software for further information.

Data

Policy information about availability of data

All manuscripts must include a data availability statement. This statement should provide the following information, where applicable:

- Accession codes, unique identifiers, or web links for publicly available datasets
- A description of any restrictions on data availability
- For clinical datasets or third party data, please ensure that the statement adheres to our policy

The vegetation plot data from the European Vegetation Archive (EVA), including plots from the ReSurveyEurope initiative that is comprised also of the LOTVS, GLORIA, and forestREplot databases, belong to the owners or contributors of each vegetation dataset. The lead author has data use rights for the purpose of Project 189 "Rewilding". Access to the vegetation data can be requested at http://euroveg.org/eva-database-obtaining-data. Climate data is openly available from the Climate Data Store at https://cds.climate.copernicus.eu/datasets/reanalysis-uerra-europe-single-levels?tab=download. Species indicator values are openly available accessible from their respective articles. The gap-filled dataset on plant functional traits was requested from the TRY Plant Trait Database together with the EVA data request under Project 189 "Rewilding". Elevation data is openly available from EuroDEM 2023 at www.mapsforeurope.org/datasets/euro-dem.

Research involving human participants, their data, or biological material

Policy information about studies with human participants or human data. See also policy information about sex, gender (identity/presentation), and sexual orientation and race, ethnicity and racism.

Reporting on sex and gender	NA
Reporting on race, ethnicity, or other socially relevant groupings	NA
Population characteristics	NA
Recruitment	NA
Ethics oversight	NA

Note that full information on the approval of the study protocol must also be provided in the manuscript.

Field-specific reporting

Please select the one below that is the best fit for your research. If you are not sure, read the appropriate sections before making your selection.

☐ Life sciences ☐ Behavioural & social sciences ☒ Ecological, evolutionary & environmental sciences

For a reference copy of the document with all sections, see nature.com/documents/nr-reporting-summary-flat.pdf

Ecological, evolutionary & environmental sciences study design

All studies must disclose on these points even when the disclosure is negative.

Study description	Quantifying and analysing the detectable severity of climate-change and disturbance-driven disequilibrium for vascular plant species in Europe
Research sample	Vascular plant species presence-absence from vegetation plots in Europe
Sampling strategy	No sampling procedure was done for this study. All available vegetation plot data from the European Vegetation Archive that were within Europe (as delineated in our methods) and within our study period (detailed below) were used. Data exclusions are detailed below.
Data collection	No data collection was done for this study.
Timing and spatial scale	We selected for vegetation plot data within our study period (1981 - 2018). We selected for plots within Europe, but excluding Ukraine, Russia, Belarus, and Turkey.
Data exclusions	We excluded vegetation plots with location uncertainty exceeding 5 km, noted as experimentally manipulated, or lacked a date of recording. We excluded observations (within plots) with taxonomic identification above the species level (including section, series, and subgenus), hybrids, uncertain identities (cf.), and cultivated individuals.
Reproducibility	Multiple model performance metrics and statistical tests of pairwise significance were used to confirm the main finding. Code used to develop the four modelling protocols were made available.

Randomization	For each modelling protocol, 10 replicates were performed per species, each using a randomly selected 90% of the training data.
Blinding	NA

Did the study involve field work? ☐ Yes ☒ No

Reporting for specific materials, systems and methods

We require information from authors about some types of materials, experimental systems and methods used in many studies. Here, indicate whether each material, system or method listed is relevant to your study. If you are not sure if a list item applies to your research, read the appropriate section before selecting a response.

Materials & experimental systems

n/a	Involved in the study	
☒	☐	Antibodies
☒	☐	Eukaryotic cell lines
☒	☐	Palaeontology and archaeology
☒	☐	Animals and other organisms
☒	☐	Clinical data
☒	☐	Dual use research of concern
☒	☐	Plants

Methods

n/a	Involved in the study	
☒	☐	ChIP-seq
☒	☐	Flow cytometry
☒	☐	MRI-based neuroimaging

Plants

Seed stocks	NA
Novel plant genotypes	NA
Authentication	NA
