# Supplementary material for: Disturbance regimes drive widespread plant range disequilibrium in Europe alongside climate change
Source: Nat Ecol Evol. 2026 Jul 7;10(8):1475–87. doi: 10.1038/s41559-026-03119-w (PMC13441915; doi:10.1038/s41559-026-03119-w)
Supplement: Supplementary file 3 [file 41559_2026_3119_MOESM3_ESM.pdf]

nature portfolio

Peer Review File

Disturbance regimes drive widespread plant range disequilibrium in Europe alongside climate change

Corresponding Author: Dr Sean E. H. Pang

Version 0:

Decision Letter:

1st July 2025

*Please ensure you delete the link to your author homepage in this e-mail if you wish to forward it to your co-authors.

Dear Dr Pang,

Your Article entitled "Plant range disequilibrium in Europe is shaped more by disturbance than climate change" has now been seen by three reviewers. As you will see from their reports below, they find your work of considerable potential interest but have raised major concerns. In light of their feedback, we would like to see a major revision of the manuscript.

We hope you will find the reviewers' comments useful as you decide how to proceed. Please do not hesitate to contact us if there are specific requests from the reviewers that you believe are technically impossible or unlikely to yield a meaningful outcome. However, please bear in mind that we will be reluctant to continue the review process in the absence of additional analyses to address the reviewers' technical concerns.

If revising your manuscript:

* Please highlight all changes in the manuscript text file and provide it in Microsoft Word format.

* Include a “Response to reviewers” document detailing, point-by-point, how you addressed each referee comment. If no action was taken to address a point, you must provide a compelling argument. This response will be sent back to the referees along with the revised manuscript.

* If you have not done so already we suggest that you begin to revise your manuscript so that it conforms to our Article format instructions at http://www.nature.com/natecolevol/info/final-submission. Refer also to any guidelines provided in this letter.

* Include a reporting summary (https://www.nature.com/documents/nr-reporting-summary.pdf) and an editorial policy checklist (https://www.nature.com/documents/nr-editorial-policy-checklist.pdf). These will be available to referees to aid in their evaluation if the manuscript goes back for peer review. These checklists should be opened and edited in Adobe Reader or similar software, not in the browser.

We would prefer to receive the revised manuscript within 6 months. If you cannot send it within this time, please let us know. We will be happy to consider your revision so long as nothing similar has been accepted for publication at Nature Ecology & Evolution or published elsewhere.

Once ready, please use the link below to submit a revised paper:

Link Redacted

Note: This URL links to your confidential home page and associated information about manuscripts you may have submitted, or that you are reviewing for us. If you wish to forward this email to co-authors, please delete the link to your homepage.

Nature Ecology & Evolution is committed to improving transparency in authorship. As part of our efforts in this direction, we are now requesting that all authors identified as ‘corresponding author’ on published papers create and link their Open Researcher and Contributor Identifier (ORCID) with their account on the Manuscript Tracking System (MTS), prior to acceptance. This applies to primary research papers only. ORCID helps the scientific community achieve unambiguous

attribution of all scholarly contributions. You can create and link your ORCID from the home page of the MTS by clicking on 'Modify my Springer Nature account'. For more information please visit please visit www.springernature.com/orcid.

Please do not hesitate to contact me if you have any questions or would like to discuss the required revisions further.

Thank you for the opportunity to review your work.

[redacted]

Reviewers' comments:

Reviewer #1 (Remarks to the Author):

This is an interesting paper and the analyses appear robust and the hypotheses are novel. I can follow the concept of climate disequilibrium but disturbance disequilibrium, as discussed here, seems to just equate with environmental filtering along a disturbance gradient. I can understand this idea better in terms of colonization credit and extinction debt but to demonstrate these disequilibria requires information on past disturbance to enable contrast with the present and specifically to determine whether the current species composition is better predicted by past disturbance regimes. In their manuscript the contrast in fit between models trained on present versus past is therefore evident for climate but not so for disturbance Their treatment of disturbance seems more akin to a test of models with versus without static disturbance variables. If so this defaults to addressing a question whose answer is already well known; that species are strongly filtered by disturbance. I therefore understand what they mean by disturbance disequilibrium but it isn't obvious that this is what was tested hence the claim in the title of the paper is not proven to my mind at least. The climate lag analyses are very interesting as is the modification of these lags via disturbance and plant traits and so perhaps the best approach would be to recast the analysis and the title of the paper along the lines of "plant range disequilibrium due to climate change is dependent on disturbance".

I only have one detailed comment: The shape of the temporal lag versus CC plots in Fig 4a is interesting. However, my understanding from the Methods section is that the plot data ranged from 1981 to 2003. So could these patterns be artefacts of the varying temporal distribution of occurrences interacting or not with the spatial location of those occurrences. For example, if climate change has only been significant from say 1990 to 2003 but not very marked from 1960 to 1981 then a species sampled to a greater extent in 1981 would be less likely to show a lag than if it were sampled in the more recent period. Moreover, if in the latter case the species was sampled from near to its historic range edge in the later period then lagged effects might be even more likely to apply because. How would such temporal and spatial biases in sampling of individual species be likely to result in artefactual patterns? In this respect wouldn't it have been better to use data from the whole period up to 2018 but randomise representatively across space and time with random and representative selection of a validation dataset? If these artefacts are possible then one consequence could be that climate lags and their risks are not well quantified undermining the author's conclusion that "climate-change disequilibrium is less severe now (line 415)".

Reviewer #2 (Remarks to the Author):

The authors investigated why species distributions often fail to match their expected climatic niche, resulting in range disequilibrium. They quantified disequilibrium by comparing a "standard" SDM incorporating static climate-only variables with models that more explicitly account for disequilibrium due to climate change or due to disturbance (or both). The SDMs utilize an impressive amount of data from a large number of species across Europe. The major finding is that disturbance disequilibrium is far more ubiquitous and pronounced than is climate change disequilibrium. The authors conclude that disturbance, not only climate change, is a main driver of disequilibrium in these vascular plant species, and that accounting for both disturbance and climate change is essential for predicting biodiversity changes.
I found the manuscript to be generally well-written, with a clearly-stated purpose and sufficient background to understand its motivation and rationale. I think that the question is certainly topical and would be of general interest to a wide range of readers, and I generally agree with the main conclusion that it is important to consider drivers, such as disturbance, beyond just climate change when investigating range disequilibrium. I do have several major concerns, mostly about the methods, that potentially affect the main results and conclusions of the manuscript. I also think that the discussion, while thorough and generally well-written, is rather long for a journal like Nature E&E and could be made more concise. I highlight several key points of concern that, at a minimum, need to be made clearer in the manuscript. More drastically, however, I suggest that the authors need to make several changes to their SDM approach to better disentangle the effects of climate change and disturbance on range disequilibrium. Below, I summarize my main concerns and then give other comments. I hope that the authors find these comments useful for improving the manuscript.

Major concerns:
1. Model-inherent errors are not explicitly considered, so all errors are effectively assumed to be "ecological errors" (i.e., disequilibrium)

There appears to be no attempt to quantify or correct model-inherent errors (false presences and false absences) although certainly some proportion of the mismatches between SDM predictions and data must be attributed to model-inherent errors. As a result, it appears to me that all errors are effectively categorized as ecological errors that indicate disequilibrium, which could artificially alter their estimates of disequilibrium. Worse, it is not clear whether different SDMs (e.g., climate-change vs. disturbance) would be more or less susceptible to model-inherent errors, which could systematically bias their results. Furthermore, by not quantifying model-inherent errors, it seems to me that SDMs for all species are effectively included in the downstream analyses, regardless of whether the SDMs perform well or not. If I am mistaken, then at a minimum, I think that the manuscript should be revised for clarity. Otherwise, I am not even sure whether model-inherent errors would systematically inflate or deflate their disequilibrium estimates because model errors could, in principle, artificially produce a mismatch (e.g., an incorrect projection of a presence where an absence is observed) or a match (e.g., an incorrect projection of a presence where an absence is observed).
This last point highlights a fundamental challenge that the authors have to face: how to disentangle model-inherent and ecological errors when the same data has seemingly been used for both tasks. My suggestion is to use split-sampling to train and test the SDMs using independent subsets of the same time period (e.g., 1981-2003) to quantify model-inherent errors, then project the SDMs to the latter period (2004-2018) to quantify disequilibrium, and finally correct the disequilibrium estimates using the information about model-inherent errors from the first step. Model-inherent errors could also be used to exclude species with poorly-performing SDMs from the downstream analyses, which would help to address my concern that current disequilibrium estimates may be biased by effectively including all species in the analyses, regardless of SDM performance. For example, it is fairly common to use a TSS score of >0.6 (or even >0.7) to exclude species with poorly-performing SDMs. I suggest that the authors can look at Rumpf et al. (2019) for a broadly similar correction of model-inherent errors (with a different methodological approach). If my concern is valid, the authors need to carefully rethink how ecological errors can be disentangled from model-inherent errors.

2. Quantifying disequilibrium by comparing predictions of static climate-only model with dynamic climate model and disturbance model

The authors apparently quantified disequilibrium severity via the difference in performance between the disequilibrium SDMs and the standard static climate-only SDM, using Cohen's D from pairwise t-tests between different SDM types (lines 159-162). They then evaluated spatial patterns of disequilibrium by identifying plots where the standard SDM was incorrect, but the climate-change or disturbance model was correct (lines 175-177). I sort of understand the rationale that using the static SDM as a baseline reference model allows the authors to cleverly quantify how much explanatory power is gained by accounting for climate change or disturbance, but I am still not convinced that these definitions are the most appropriate for quantifying disequilibrium. In both cases, it seems to me that the static SDM is effectively used as a baseline upon which to compare the climate-change and disturbance SDMs. I find this approach a little unsatisfactory because the static SDM arguably underrepresents the current best practices in SDMs. The static SDM may therefore provide a weak baseline that both disequilibrium models can relatively easily outperform, which may in turn inflate the apparent contributions of the climate-change or disturbance SDMs (or both) to reducing disequilibrium. Moreover, I am not convinced that plots where the standard SDM is incorrect but the disequilibrium SDMs are correct are definitive evidence of disequilibrium. What about plots where the disequilibrium SDMs are incorrect (and perhaps the static SDM is even correct)? I guess these plots are just ignored, perhaps discounted as model errors. Rather than quantifying disequilibrium as differences between more-advanced SDMs and a somewhat arbitrary baseline SDM, I would like to see disequilibrium quantified more explicitly via actual differences between SDM projections and observational data.
I think that the static SDM is not needed at all and that the authors should compare the climate-change and disturbance SDMs directly against each other and then see if there is any gain in explanatory power by incorporating both factors. An obvious issue is what to do about the climate variables in the disturbance SDM. One solution is to simply use a disturbance-only SDM (one could even argue that if disturbance is a major driver of disequilibrium, then an SDM incorporating only disturbance might be the best performing model). Alternatively (but perhaps less desirably), the authors could compare their climate-change SDM with their SDM incorporating both factors and, by subtraction, infer the effects of disturbance. Either way, I am not convinced that the static SDM is particularly useful and it may even obscure interesting results gained by directly comparing disequilibrium SDMs with observed data.

3. Using randomly permutated values for disturbance-related predictors in disturbance-uninformed SDMs

The authors attempt to control for the number of predictor variables across different SDM types by using the same disturbance-related predictors in disturbance-uninformed SDMs, but with values that were randomly permutated for both training and testing datasets (lines 599-601). I think this choice is problematic and may artificially explain why disturbance SDMs strongly outperformed climate-change SDMs (Fig. 1). By incorporating randomly permuted variables in the climate SDMs (presumably, both static and dynamic), I think that the authors inadvertently may have artificially penalized the climate-only SDMs by effectively forcing them to fit noise, which could degrade their performance and lead to misleading comparisons. I do not believe that this is common practice in SDMs. I would accept (perhaps acknowledge) the unequal number of predictors and just compare model performance directly. Essentially, the authors are evaluating the hypothesis that the 'best' disturbance SDM will outperform the 'best' climate-change SDM, which does not require the SDMs to be 'equal'. Alternatively, if the authors really want to penalize complexity, they could use AIC or cross-validation-based metrics, but this seems extraneous to me.

4. Violating the quasi-equilibrium assumption of SDMs

The authors calibrated their SDMs using climate data from 1981 to 2003, but the climate, of course, was rapidly changing during this period due to climate change. This is problematic because SDMs inherently assume that species are in quasi-

equilibrium with the environment, which is unlikely during this period of accelerating climate change. Presumably, this period was used because their climate data goes back to 1961, thus ensuring that "all plots had at least 20 years of preceding climate data" (lines 494-495). The authors then test their SDMs using independent data from 2004 to 2018, but this approach implicitly assumes that the climate remains largely unchanged between these two time periods (i.e., that the species is still in quasi-equilibrium with the climate). This assumption is almost assuredly false because of accelerating climate change. The authors then use AUC to evaluate SDM performance. But why would AUC in a future time period be the definitive metric for SDM performance? My concerns are that the authors have inadvertently violated a major SDM assumption and then assumed that better predictive performance indicates more realistic climate tracking, when occurrence data may not actually reflect climate suitability if species are in disequilibrium.
I see two potential solutions for these issues, if they are tractable. The best approach, I think, would be to calibrate the climate SDMs using earlier data that are perhaps less likely to violate the quasi-equilibrium assumption than are the rapidly changing 1980s and 1990s. I understand that climate data may be unavailable earlier and there are likely fewer observation data from earlier with which to calibrate the SDMs, but other climate data (e.g., CHELSA) go back further in time, so this option could be possible, in principle. If this option is not viable, then I suggest that the authors be transparent about the quasi-equilibrium assumption. Either way, I think that it would be best to train and test the SDMs using independent subsets of the same time period (see concern 1). By projecting SDMs into the more recent time period, the authors would predict species' expected ranges if they were to track climate change, which when compared to observation data, would be consistent with the concept of disequilibrium.

5. Understanding how disturbance is incorporated within the SDMs

In my opinion, a key novel contribution of this study is using a SDM approach to investigate the role of disturbance in driving range disequilibrium; however, how disturbance is actually incorporated into the SDMs remains largely a black box to me. I found it difficult to assess their approach because much of the methods relating to disturbance were rather opaque. My understanding is that the authors used three disturbance-related predictors that they could quantify in their dataset (how generalizable then are these predictors beyond this dataset?). I do not fully understand the two disturbance severity indicators, but apparently, they quantify the proportion of biomass lost during disturbance events at either the whole- or herbaceous- community level (lines 528-532). So, for every plot, was the biomass loss estimated as the change in biomass between the two time periods? Or only plots with a "disturbance event" (and, if so, how is a disturbance event defined)? The authors quantified these variables at the plot-level, after excluding focal species to avoid circularity (Lines 539-541). I think that I understand why these variables were not simply calculated at the species-level because then disturbance severity would be zero anywhere a focal species is absent. That said, quantifying disturbance in this way is potentially problematic. Sites with large reductions predominately in the focal species would be defined as low disturbance (i.e., little community biomass loss). Conversely, sites with little reduction in focal biomass, but large reductions in other species' biomass would be defined as high disturbance. Perhaps this issue could be resolved by simply including focal species in community-derived indicator values (ignoring circularity issue). Or perhaps I have missed something crucial here. The key point is that without more detailed explanations of the disturbance variables and how they are incorporated into the SDMs, I think that it is difficult to evaluate their approach and clearly understand the manuscript. At a minimum, the authors missed an opportunity to highlight a novel contribution of their study.

6. Addressing potential spatial/temporal bias and niche truncation issues

The dataset that the authors use is truly impressive, but of course, it does not fully sample the environment across space and time. I think the authors, at a minimum, need to acknowledge potential spatial and temporal bias and discuss how this might affect their results. I think that spatial bias is particularly worrisome because we have no idea whether, for example, warmer or more disturbed sites happen to be observed more frequently in recent years, which could bias estimates of the effects of climate change or disturbance. This issue may be compounded by the fact that sites are apparently rarely re-sampled, so there is an entirely different set of sites between the two time periods. I do not see an easy, immediate solution here. Perhaps the authors could statistically compare the climate and disturbance regimes to evaluate if there are significant differences between the two periods (seems likely for climate variables due to climate change). Or, more ambitiously, perhaps the authors could model a larger number of sites to assess how representative the observed sites are relative to the larger dataset (this would presumably only work for the climate SDMs). This is a tricky issue, but I suggest that the authors think carefully about how to evaluate potential biases in their data and at least briefly discuss these issues in the manuscript.
The other potential issue that I see with the SDMs is niche truncation, where the SDMs essentially fail to capture the full environmental niche and are therefore forced to extrapolate to novel environments. Niche truncation could certainly be an issue with the climate variables because climate change may expose species to novel climates in which a truncated SDM may incorrectly extrapolate suitability. It could also be an issue with disturbance predictors if only a subset of the 'disturbance regime' is capture by the SDM, which is forced to extrapolate to novel disturbance regimes in the future. There are a growing number of studies dealing with niche truncation, but I would generally suggest caution when extrapolating beyond observed environmental space, and encourage the authors to try to diagnose niche truncation and then remove species with truncated niches from further analyses. Again, at a bare minimum, this issue should at least be acknowledged and briefly discussed in the manuscript.

7. Justifying SDM decisions and report SDM protocols

Generally, I suggest that the authors justify their decisions in building the SDMs whenever possible and ideally report their SDM methods in a more standard protocol. I attempted to highlight lines in the manuscript where I felt that more justification was needed in the "other comments" section below. Some of my major questions are: why are only GAMs used? Why not use ensemble models? Why are species with poorly-performing SDMs not excluded from downstream analyses? In terms of

SDM protocols, there is a growing effort in the field to standardize and document SDM protocols in order to make SDM studies more transparent and reproducible. I advocate for the ODMAP protocol (Zurell et al. 2020), but more generally, I suggest that the authors use some more standard protocol to report their SDMs.

References:
Rumpf et al. 2019. DOI: 10.1038/s41467-019-12343-x
Zurell et al. 2020. DOI: 10.1111/ecog.04960

Other comments:

• Line 146: When I first read about the lag intervals, my concern is that 18 years is a long time to assume that occurrence data accurately reflects climate suitability, even for long-lived plant species. In other words, especially given rapidly accelerating climate change in the decades used in this study, how do we know that presences really indicate that the climate 18 years ago was suitable or that absences indicate that the climate 18 years ago was unsuitable? The potential issue is disequilibrium due to the changing climate. Could even a long-lived plant have colonized a formerly unsuitable habitat or gone extinct from a formerly suitable habitat over the intervening 18 years? I am not entirely convinced that long lags are more useful for investigating climate disequilibrium, even if this is a somewhat standard procedure in SDMs for capturing legacy effects.

• Line 150: I thought that light values were species-specific, but disturbance severely was community-derived, but this line seems to imply that all are community-level metrics.

• Line 158: Very little detail is given in the main text about the SDMs themselves. What algorithms were used? Were ensemble models used? These details become apparent in the Methods, but a little more information here might be useful.

• Line 159: I was initially confused why pairwise t-tests were used to compare models when there are more than two models. It later became clear that the authors were comparing each disequilibrium model to the standard model using pairwise t-tests, but the wording here was initially a little confusing.

• Line 160: The authors use TSS in addition to AUC in the Supplementary Information, but this is not obviously referenced in the manuscript. This line might be a good place to show that the main results hold when another performance metric is used.

• Lines 161-162: Cohen's D gives the magnitude of the effect size difference, but we also might care about the direction of the effect in this case. Presumably, all disequilibrium SDMs outperformed the standard SDM, so Cohen's D reflects an increase in model performance.

• Line 163: "Various species attributes" is somewhat vague. Why do these attributes matter biologically? Why were they selected over many others that presumably could have been used? Also, would it not be more appropriate to scale the disequilibrium estimates by the total presences to control for the fact that more widely-distributed (or sampled) species may just have more chances to exhibit disequilibrium?

• Line 175: The comparison is "between plots where species were present and plots … where disequilibrium occurred." Presumably, plots "where species were present" actually means plots where the species was predicted and observed to be present. As written, it seems like there might an issue if plots where the species was present but was predicted to be absent were included in both subsets being compared.

• Lines 191-192: It might be useful to always include the group being compared to when making a comparison. Here, for example, "the number of species with significantly improved AUC was higher for models that already accounted for disturbance." Higher than what? This was done throughout the manuscript. Sometime it is trivial to know what the comparison was between, but sometime it was not immediately obvious. I suggest making the reader's life easier and just including the full comparison every time.

• Line 194-195: Similar to above: "with similar average gain in performance." Similar to what? Again, this sentence might be trivial, but I would be as specific as possible with these statements.

• Line 200: Were 3,037 species included in each type of SDM shown in Figure 1. I would just explicitly report the sample size here.

• Line 216: What exactly is temperature annual range?

• Lines 219-227: I would reference the appropriate figure panels in this paragraph.

• Lines 225: "In the full model." This could be confusing if one conflates the full statistical model with the "full" SDM incorporating both climate and disturbance.

• Line 228: "When light availability and disturbance severity were excluded due to multicollinearity (Methods), additional associations emerged…" With multicollinearity, I am not sure how relevant the analyses are that include these two variables. Perhaps it would be better to only present results with these variables removed from the analyses.

• Line 228-236: Going back to my comment about more biological justification for the variables used (comment on Line 163), I found it a little difficult to keep the main hypotheses in mind while reading this paragraph without a stronger justification for why, biologically, all of these variables are included in the analyses here.

• Figure 2: Why do some variables include a nonlinear term and others do not? Also, there is a potential red-green colorblindness issue here with the labels.

• Line 251: “Primarily reflected overpredictions” is vague. How many overpredictions versus underpredictions were there? This point is important because it effectively justifies why only overpredictions are presented in the main text.

• “Lags in plant range responses” subsection and Figure 4: I struggled with the placement of this section in the manuscript. At a minimum, I think that there needs to be a strong rationale for these analyses. Are they testing a specific biologically-motivated hypothesis or are they needed methodologically to build the best-performing climate-change SDM to identify climate-driven disequilibrium. As is, I am not sure whether this section should come earlier in the manuscript to help understand the climate change SDMs, stay where it is, or be moved to the Supplementary Information to “streamline” the paper. As is, my opinion is that this section should be moved to the Supplementary Information to focus the paper concisely around the topic of climate- and disturbance-driven disequilibrium.

• Lines 294-309: I found it difficult to distill the key findings from this paragraph. It sort of seems like somewhat idiosyncratic results (some species show this pattern, but other species show that pattern) with perhaps little general insight or inference. This paragraph contributed to my above comment questioning the rationale for this subsection. It is also the results from only 12 species (which, to be fair, is acknowledged and justified by the authors), but I nonetheless struggled with the main findings of this paragraph.

• Lines 306-308: I am a little confused about the finding that “best-performing windows were predominately older … (Fig. 4b)”. Figure 4b seems to show that, for most species, the static climate model was best performing (which, if true, begs the question why use the lagged models at all?).

• Lines 310-317; Figure 4c: I do not fully understand the rationale for this analysis. What is the hypothesis being tested here? That these traits correlate with the lag length of the best-performing window?

• Lines 318-322: Similar to my comment on Line 228, I would check for multicollinearity and then only report results for the model with variables removed.

• Lines 354-379: This paragraph is very detailed, but I would reference the relevant figure panels throughout the paragraph to help readers quickly find the result being discussed.

• Lines 490-491: Why were plots in these countries excluded?

• Line 496: Why were plots without coordinate uncertainty information retained? Would it not be more cautious to exclude them?

• Lines 497-499: Were any attempts made to harmonize the taxonomic names? There are R packages (e.g., WorldFlora) for doing this, for example.

• Lines 508-510: Why was this climate dataset used? The spatial resolution is fairly coarse (which the authors acknowledge).

• Lines 510-514: These lines are at the crux of my concern about the quasi-equilibrium assumption (concern 4). I would think that data before “major anthropogenic climate shifts began around the 1960” would be insanely valuable because species are most likely in quasi-equilibrium with the climate during this period. Using these data would thus give the best estimates of species’ full realized climate niches. As I argue in concern 4, I strongly advocate for training and testing the SDMs during as early a time period as possible to avoid strongly violating the quasi-equilibrium assumption of SDMs.

• Lines 520-524: I would briefly explain some these variables for general readers (e.g., mean diurnal range, isothermality, temperature/precipitation seasonality, frost change frequency). How were variables included in the climate SDMs selected? Also, annual mean temperature is often criticized as not particularly informative biologically (annual precipitation may suffer similarly). Finally, while the number of variables included in an SDM is, to a certain extent, a personal choice, I think that 8 variables is a lot and that the authors risk overfitting the SDMs. Personally, I would select 2-4 predictors, justified in some manner (PCA loadings, etc.).

• Lines 562-564: Presumably, spatial thinning was done for both the testing and training datasets, right?

• Line 565: Why where the thresholds of 35 training and 10 testing presences selected? One fairly common heuristic is 10 presences per predictor variable, which would yield a much higher threshold of 80 presences for the current SDMs. This heuristic is only one option, so lower thresholds could be reasonable if justified.

• Line 566: Why was 300km uses as a distance threshold? Also, I understand the rationale to impose this limit to avoid absences due to dispersal limitations, but it is also yet one more somewhat arbitrary threshold imposed upon these SDMs. If

SDMs are calibrated under quasi-equilibrium (e.g., with earlier climate data), then I suppose that one could argue that dispersal-limited disequilibrium is less likely, and thus preclude this threshold.

• Line 573: 5-year moving windows seem reasonable, but it there justification for using say 11-year windows that might better capture decadal variation at the demographic scale of these long-lived plants? Again, I suggest that the authors justify modeling decisions if at all possible.

• Line 592: Why were models trained on a 70% subset of the data from the training bin when, as far as I can tell, the remaining 30% of the data was not used at all?

• Line 593: Why were only GAMs used? Were standard parameter values used in the GAMs?

• Lines 614-627: The rational for not binarizing projections based on the threshold that maximized the TSS score has clearly been thought out, but I do not fully follow the logic here. I do not completely see why using the TSS threshold would "misallocate these sites equally between predicted presences and absences – artificially minimizing the apparent disequilibrium signal (or its leaning towards overpredictions) by balancing false positives and negatives"? I would think that there is equal likelihood of the SDMs – including the disturbance SDM – making false positives or false negatives, so the TSS score would be appropriate. Here, my intuition is that I have missed something, but it highlights an area where the authors may consider revisions to improve clarity.

• Line 653: Does the spatial resolution of the elevation data match that of the climate data?

• Lines 661-664: Why where these variables suspected to have unimodal relationships and not other variables?

• Lines 680-683: Does this mean that re-sampled sites are excluded from the dataset or simply that they could not use only re-sampled sites because there are far too few of them, making it impossible to quantify extinction debts and colonization credits? I think that these are important points that should be made far earlier in the Methods, perhaps in the "Plot and environmental data" subsection.

• Line 694: What exactly is meant by significant disequilibrium?

• Line 701: There are 1,434 species with significant climate-change disequilibrium, but Figure 4c shows way more species than this in the "null" category alone. Also, is 1,434 essentially the sum of the "recent" and "older" categories in Figure 4c?

Reviewer #3 (Remarks to the Author):

Review of Pang et al., submitted to Nat Ecol Evo (2025)

The manuscript of Pang et al. addresses an important issue, examining distribution disequilibrium in vascular plant species across Europe. The manuscript efficiently details the context and relevance of range disequilibrium, and neatly contrasts and disentangles disequilibrium driven by climate change and disturbances in this system. The research uses several well-established modeling methods and existing datasets together in a relatively novel way to quantify, and describe patterns in, disequilibrium driven by climate change and/or disturbances. These methods are described clearly and are appropriate for the data and manuscript aims. The scale of the analyses is across the whole of Europe, and the sample sizes used are large and are clearly reported. I particularly appreciate that the authors have already thoroughly addressed many of the methodological issues that could be problematic in a study of this nature.

The manuscript is well-written, communicating most of the ideas and findings clearly. Overall, I have mostly relatively minor comments, with only a few points that I think maybe need some more careful consideration (listed below in detail). Broadly, due to the very concise nature of the manuscript (given the remarkable breadth and depth of analyses conducted), there are a few statements that require (preferably) some more explanation or (at least) some re-wording to help readers (detailed below).

I believe this manuscript would be of interest to a broad audience, and that it has the potential to have a significant impact on the general thinking about modelling species distributions and the use of SDMs for conservation planning.

Issues for the authors to consider:
1) Is the nature and rate of change in climate consistent across both the training and testing periods and across the study region? I realize a detailed analysis of this is likely well beyond the scope of this paper, but I think a statement about this would be useful for assisting with interpretation. For example, could the multiple peaks in some species lagged responses potentially be reflecting strongly variable inter-annual climatic conditions (i.e. two peaks = two temporally-separated time periods where conditions were similarly favourable for the establishment of a species)? And could difference in inter-annual variability or climate trends between the training and testing period affect the findings?
2) Would it be possible to complement the results based on changes in AUC with quantifying the (total or proportional) change in the species' range size associated with the inclusion (or exclusion) of climatic lags and/or disturbance indicators? A higher AUC indicates a better fit to reality, but matching that change in AUC with how large a difference it would make to

the species distribution range could be useful for interpreting the implications of the findings.
3) The colours used in Fig. 3 are difficult to distinguish, especially with many overlapping points; increasing the size of the symbols in the legend would be useful, but the authors may want to explore other colour schemes or plotting arrangements to improve the differentiation of the four groupings.
4) I would encourage the authors to consider visualizing a selection of the results from Fig. 2 as a scatterplot, since in the Discussion it is emphasized that "disequilibrium instead peaked at intermediate values across all three indicators" (line 356). I think that visualizing this finding would be useful for readers (and could be a good complement to Fig. 3).
5) Is there any spatial pattern in the proportion of species that show significant disequilibrium? As the analyses reported under the "Spatial distribution of disequilibrium" section are limited only to a subset of the initial species (c. 80% and 20% for disturbance and climate change disequilibrium), it could be informative to test for spatial patterns in the proportion of species exhibiting disequilibrium.
6) Some more speculation about the expansion of these ideas beyond the European context could benefit the manuscript; the authors do touch on this broadly, but some explicit hypotheses about which regions, taxa or disturbance types might show similar or different patterns would likely be of interest to readers.
7) It would be interesting to see a visualization of the distribution (or density) of vegetation plots, and of the different disturbance indicators (i.e. a map in the supplementary materials).

Minor points:
1) Line 136: Providing citations to point readers to previous studies that have addressed this question (e.g. outside of temperate ecosystems) would be useful here.
2) Line 165 - 167: The motivation for this expectation is not clear from the preceding text, and should likely be strengthened (especially since the text also states "it remains unresolved whether climate change or disturbance regimes more strongly drive species-level distributional mismatches" (line 137 – 138). See also line 354.
3) Line 169: Similar to the previous comment, the motivation for why species associated with open habitats would show the strongest disequilibrium is not clear.
4) Line 215: The concept of "temporal shifts in observable species-habitat associations" is raised here, but not addressed subsequently in the manuscript. Could the authors expand on this idea?
5) Line 265: Does this spatial concentration just reflect the distribution of adequately sampled species? (see also comment #7 above)
6) Line 343: "the dominant force shaping plant distributions" is a bit ambiguous here; rewording to reflect that disturbance disequilibrium is more influential than climate change disequilibrium would help avoid confusion.
7) Lines 362: some further examination of the idea of "Species with trait and habitat associations around these thresholds…" would be useful for readers and could provide the stimulus for readers to examine this in their own systems; for example, what trait values (or habitat associations) do the data suggest occur around the disturbance levels associated with maximum disequilibrium?
8) Line 408: To improve clarity, the authors might consider adding some context when citing Salguero-Gómez et al. (2016) here, as that paper does not deal with climate change disequilibrium (which could be assumed from the current format), but instead supports that taller species are typically longer-lived.
- Similarly, the authors should consider rewording lines 415-416, as the current wording mixes the findings from this study (that "climate-change disequilibrium…. is biologically relevant") with findings from previous studies (that "climate-change disequilibrium… is … likely to intensify"). Clarifying this in lines 415-416 also help to provide the motivation in line 480 that the "signal of climate change disequilibrium" is "growing".
9) Lines 403-404, 409 – 410 and 411-412: In all cases, very interesting ideas are suggested by the authors, but no further explanation or detail is provided for a reader to assess the ideas. For example, what disturbance-adapted traits are likely to lead to longer lags?
10) Line 508: As the manuscript talks about macro- and microclimate, it would be useful here to clarify what "near-surface" refers to.
11) Line 526: For readers unfamiliar with this dataset, consider explaining what it meant by "species aggregate values".
12) Line 550: Consider adding another reference or rewording this sentence slightly, since reference 68 does not deal with how soil moisture or nutrients are more strongly related to macro- than micro-climatic conditions.
13) Line 694: Consider explicitly stating that this is based on significance as assessed by the pairwise t-tests.

Version 1:

Decision Letter:

19th January 2026

*Please ensure you delete the link to your author homepage in this e-mail if you wish to forward it to your co-authors.

Dear Sean,

Thank you for your patience while your manuscript entitled "Plant range disequilibrium in Europe is shaped more by disturbance than climate change" was under review. We have now received comments from two of the original reviewers (your responses to the missing reviewer were also checked). As you will see, the reviewers found that many of their earlier concerns have been resolved, but Reviewer 2 still has some important criticisms along with recommendations on how to address them. Therefore, we would like to see further revisions along with a response to the points raised.

We are committed to providing a fair and constructive peer-review process. Do not hesitate to contact me if there are specific

requests from the reviewers that you believe are technically impossible or unlikely to yield a meaningful outcome.

When revising your manuscript:

* Please provide the manuscript in Microsoft Word format and highlight all changes in the same file or a copy.

* Include a “Response to reviewers” document detailing, point-by-point, how you addressed each reviewer comment. If no action was taken to address a point, you must provide a compelling argument. This response will be sent back to the reviewers along with the revised manuscript.

* If you have not done so already please begin to revise your manuscript so that it conforms to our Article format instructions at http://www.nature.com/natecolevol/info/final-submission. Refer also to any guidelines provided in this letter.

* Include a revised version of any required reporting checklist. It will be available to referees (and, potentially, statisticians) to aid in their evaluation if the manuscript goes back for peer review. A revised checklist is essential for re-review of the paper.

* Extended Data Figures - please ensure that any supplementary figures and tables that are crucial to the manuscript’s conclusions are converted into Extended Data figures and tables to increase visibility of these data. Extended Data figures and tables are online-only (present in the online PDF and full-text HTML versions of the paper), peer-reviewed display items that provide essential background to the article but are not included in the main article due to space constraints. A maximum of ten Extended Data display items (figures and tables) is permitted.

We would prefer to receive your revised manuscript within about two months, but if you need a substantially longer timeframe please don't hesitate to inform us. Please use the link below to submit your revised manuscript and related files:

Link Redacted

Note: This URL links to your confidential home page and associated information about manuscripts you may have submitted, or that you are reviewing for us. If you wish to forward this email to co-authors, please delete the link to your homepage.

Nature Ecology & Evolution is committed to improving transparency in authorship. As part of our efforts in this direction, we are now requesting that all authors identified as ‘corresponding author’ on published papers create and link their Open Researcher and Contributor Identifier (ORCID) with their account on the Manuscript Tracking System (MTS), prior to acceptance. ORCID helps the scientific community achieve unambiguous attribution of all scholarly contributions. You can create and link your ORCID from the home page of the MTS by clicking on ‘Modify my Springer Nature account’. For more information please visit please visit www.springernature.com/orcid.

Please do not hesitate to contact me if you have any questions. I look forward to the revised manuscript.

[redacted]

****

Reviewers' comments:

Reviewer #1 (Remarks to the Author):

I have now been through the revised MS and the authors’ response and consider that they have fully addressed my initial concerns. I was also asked to consider their response to reviewer 3 and in particular comments R68, 69 and 72. The two new figures they have provided to address 68 and 69 are impressive, apposite and very informative while their text additions to address 72 are also satisfactory . I note the large number of detailed points raised by all reviewers and so congratulate the authors on their detailed and thorough response to all of them.

Reviewer #2 (Remarks to the Author):

I thank the authors for their careful revisions and very detailed responses to reviewer comments. I think that the manuscript is more clear, transparent, and better justified as a result. I reiterate that I found this to be an interesting paper of general interest to a wide readership. The previous revisions have cleared up many of the issues identified by both reviewers, but I still have several main concerns.

My central concern is that the authors have laid out this “disturbance versus climate change” narrative in the manuscript (even in the title), which frames disturbance and climate change as alternative hypotheses dueling to explain range disequilibrium. I think that the general topic and their results presented here are far more nuanced than this, and that this disturbance versus climate change narrative is actually somewhat of a disservice to their study. After all, climate change is shifting species’ suitable habitats, but disturbance may be limiting how well species can track these shifts. So, the extent to which range disequilibrium is driven by disturbance versus climate change seems beside the point to me. Rather, the key

question is how disturbance influences climate-driven range disequilibrium (i.e., mismatches between species ranges and their suitable climates). My main suggestion is to shift the paper away from this disturbance versus climate change narrative and reframe it around the central premise that disturbance is a fundamental, but largely unexplored, mechanism explaining range disequilibrium.

The main evidence for this disturbance is more important than climate change narrative is the huge boost in AUC when incorporating disturbance. This result suggests that disturbance variables are crucial but missing environmental predictors that help to far better describe species' realized niches than do climate variables alone. So, no wonder that the disturbance-informed models outperform the climate-dynamic models; they can "see" habitat structures in the environment that climate-dynamic models cannot, and so they likely characterize much more robust realized niches. This result is certainly a key insight, but the underlying reason for it actually makes the disturbance versus climate change narrative less compelling, in my opinion. It also sets up this sort of odd juxtaposition that disturbance is not a dynamic process in the model like climate change. I think that this fundamental issue underlies many of the comments from both reviewers in response to the earlier version of the manuscript. I would reframe the paper to make the compelling argument that disturbance is a key missing mechanism driving range disequilibrium as evidenced by the huge AUC improvements with disturbance variables (regardless of how climate is incorporated into the model).

My second main concern is that climate change effects on range disequilibrium may be systematically underestimated because the model is limited to selecting a single, best-performing climate window from a relatively short climate record. This is not merely a biological interpretation issue, but a structural limitation of the modelling framework, which constrains how much climate-driven disequilibrium can be detected. The most common pattern observed in the climate window was bimodality (lines 314-315), which to me suggests that different demographic processes are likely affected at different times. For example, establishment of an alpine plant might be most strongly tied to the climate of 18+ years ago, but survival is most strongly tied to recent or current climate. Despite this biological signal, the model is forced to pick a single optimal window, eliminating any effects of other potentially important process. Limiting the model to only a single optimal window may great restrict the effects of climate change on disequilibrium, potentially explaining the relatively low gains in AUC between the climate-dynamic and climate-static models. The other potentially major issue here is that most species performed best under the static climate, and otherwise, the best-performing window was most frequently the extreme 18-year window. To me, these results suggest that perhaps, for many of these species, the model struggles to identify any strong climate signal in the record or, if it does, that the lags may often be far longer than the available climate data. This latter result suggests that climate change-driven disequilibrium is almost by definition a minimum estimate. I would, at a minimum, acknowledge that this approach is likely very conservative because the demography of many of these species may be vastly longer than the climate window being analyzed. More ambitiously, would it be possible to incorporate this bimodality more explicitly in the model; for example, by incorporating an optimal lag for each climate variable? To be fair, the authors discuss these bimodal window responses, varying lags, and potential future directions in the discussion. By reframing the disturbance versus climate change narrative, however, these important points and caveats might look less like limitations on the climate-dynamic model in a seemingly increasingly unfair AUC comparison, and more like worthy future research directions inspired by this manuscript.

Other comments:
I do not fully understand why light and disturbance regime indicators are calculated at the community-level when all the analyses are performed at the species level?

Version 2:

Decision Letter:

20th April 2026

Dear Sean,

Thank you for your patience while your revised manuscript "Disturbance regimes drive widespread plant range disequilibrium in Europe alongside climate change" (NATECOLEVOL-25051539B) was under review. As you will see from the report appended below, Reviewer 2 remains unconvinced about the interpretation and the strength of the conclusions. However, we requested Reviewer 1's opinion on the matter and this referee concurred with us that enough was done to address Reviewer 2's concerns. You will find these additional comments embedded in the report below.

On the basis of this feedback, we will be happy in principle to publish your manuscript in Nature Ecology & Evolution, pending minor revisions to satisfy the reviewers' final requests (ideally including Reviewer 2's last call for validation, which Reviewer 1 agreed would be nice to do if feasible) and to comply with our editorial and formatting guidelines.

We are now performing detailed checks on your paper and will send you a checklist detailing our editorial and formatting requirements in about a week. Please do not upload the final materials and make any revisions until you receive this additional information from us.

Thank you again for your interest in Nature Ecology & Evolution. Please do not hesitate to contact me if you have any questions.

[redacted]

****

Reviewer #2 (Remarks to the Author):

I thank the authors again for their thoughtful revisions and responses to reviewer comments. I think that the manuscript is ambitious and impressive in terms of its data and scope. I think that the previous revisions have addressed many of my concerns and have considerably strengthened the manuscript, but I still have some concerns.

My central concern is that the disturbance indicator values may better reflect ecological associations or habitat structure than the actual mechanisms driving disturbance and that, despite the authors' efforts, there may be a circularity issue with using community-dependent indicator values to predict occurrence (i.e., community membership). The authors incorporate disturbance using community-averaged indicator values for light and disturbance severity. I am not convinced, however, that these indicator values are not essentially proxies for ecological associations with other species in a plot or descriptions of habitat structure (e.g., open versus closed canopies), rather than direct measurements of disturbance processes, like grazing or fire invoked in the manuscript. [Reviewer #1: I am unsure exactly what point the reviewer is making here. Habitat structure and successional stage are driven by disturbance or lack thereof. For example grazing can maintain patch dynamics in woodland and grassland mosaics and fire can also set back and reinitiate succession]. To be fair, the authors acknowledge that "our disturbance-related predictors captured resulting vegetation structure and composition rather than direct measurements of disturbance process" (lines 474-476). This limitation, however, is presented more as a future direction, and I think that the authors need to show more conclusively that they are, in fact, incorporating disturbance drivers and not habitat structure or other ecological associations. [Reviewer #1: In my view the indicator values estimate the equilibrium conditions associated with different species' realised niches. If the reviewer is in fact arguing that there is a space for time substitution here i.e. the indicator values unjustifiably assumes equilibrium, then that is sort of a fair point but then testing the assumption of equilibrium in response to climate and disturbance is tackled head on as the theme of the paper]

Using these indicator values is certainly convenient and the authors point to their utility in ecology, but my concern is that reported gains in model performance simply reflect that the models are much better at predicting occurrence when they can effectively use proxies for community membership as predictor variables. Indeed, species can co-occur for a variety of historical, ecological, or evolutionary reasons unrelated to disturbance, so improvements in model performance with these predictor variables may not actually reflect the role of disturbance at all [Reviewer #1: This is a fair point but can be covered by caveats attaching to the use of indicator values – see Diekmann 2003 for a comprehensive review]. This methodology also creates a potential circularity issue even with the focal species removed because the model may simply predict that a focal species occurs at a given location because the habitat is deemed suitable there because it contains a community of plants typical of that focal species [Reviewer #1: This last sentence surely sums up why indicator values are so useful because they rely on the common response of co-existing plants to indicate the nature of the conditions that favour the focal species. The issue is then dealing with the fact that the species present respond to multiple causal factors and so it is true that indications can be potentially misleading. However those deriving and using the indicator values do try to control for potential confounding between such factors. In fact substituting these values for actual measurements of the environment is often unlikely to solve this problem. Consider substrate fertility whose measured values can be correlated with a complex of similarly collinear variables including pH, carbon content and soil moisture]. The authors must work to demonstrate that this potential circularity issue does not affect their results.

All other issues aside, these indicator values may still severely limit the applicability of this study because, as I understand it, one can only project this model where there is existing data (i.e., with which to calculate the indicator values in the first place), so it is impossible to forecast the model or project it to sites without preexisting data [Reviewer #1: This isn't true though because the indicator values have a history of being subject to prediction by measurements of the environmental conditions they seek to estimate e.g. Smart et al 2010; and see equations 1-4 in De Vries et al 2010. This provides the means whereby indicator values can indeed be predicted into new sites and conditions for example by predicting C/N and pH from biogeochemical models. Similar calibrations could be carried out for disturbance indicator values for example by calibration against canopy height and bare ground. Such calibration exercises also provide valuable information on the extent to which indicator values are indeed uniquely explained by the drivers they purport to indicate versus other confounders]. The authors acknowledge many of these limitations in the discussion and even suggest that future studies could use "emerging global remote-sensing data on vegetation structure, fire, and land-use intensity" (lines 499-500). To strengthen the claim that these indicator values represent external disturbances, however, I suggest that the authors provide more justification that these indicator values at least correlate with independent data related to disturbance, such as remote-sensing data on tree-cover or fire frequency, for example, even for a subset of their data [Reviewer #1: This would be nice to see if the authors could indeed do that. I think it is quite possible.]."

Other comments:

While I appreciate the revisions that the authors have done to reframe the manuscript, I still think that the manuscript reads more like a modelling exercise emphasizing the gains of employing more advance climate-dynamic and disturbance-informed models over a basic static climate-only model. To me, this narrative somewhat undercuts or at least obscures the novelty of some of their key findings. I would reframe the results even further to emphasize the novel contributions of their study in disentangling climate-driven versus disturbance-driven range disequilibrium.

Finally, I would also further highlight the bimodality result, even if it is not possible to directly incorporate it within this model

(which I now appreciate the computational challenge from their previous response). For example, this bimodal pattern might reflect not only different important biological processes (e.g., germination versus survival) as discussed, but perhaps geographical mechanisms as well. For example, perhaps species' leading range edges respond more strongly to more-recent climate, while the trailing range edges respond more strongly to lagged climates, resulting in the bimodal pattern. Also, why was a 5-year moving-window used? Does the bimodal pattern appear if different moving-windows are used?

Open Access This Peer Review File is licensed under a Creative Commons Attribution 4.0 International License, which permits use, sharing, adaptation, distribution and reproduction in any medium or format, as long as you give appropriate credit to the original author(s) and the source, provide a link to the Creative Commons license, and indicate if changes were made.
In cases where reviewers are anonymous, credit should be given to 'Anonymous Referee' and the source.
The images or other third party material in this Peer Review File are included in the article's Creative Commons license, unless indicated otherwise in a credit line to the material. If material is not included in the article's Creative Commons license and your intended use is not permitted by statutory regulation or exceeds the permitted use, you will need to obtain permission directly from the copyright holder.
To view a copy of this license, visit https://creativecommons.org/licenses/by/4.0/
